# Transcriptome-Wide Studies of RNA-Targeted Small Molecules Provide a Simple and Selective r(CUG)^exp^ Degrader in Myotonic Dystrophy

**DOI:** 10.1021/acscentsci.2c01223

**Published:** 2023-06-26

**Authors:** Quentin M. R. Gibaut, Jessica A. Bush, Yuquan Tong, Jared T. Baisden, Amirhossein Taghavi, Hailey Olafson, Xiyuan Yao, Jessica L. Childs-Disney, Eric T. Wang, Matthew D. Disney

**Affiliations:** † The Department of Chemistry, UF Scripps Biomedical Research and The Scripps Research Institute, Jupiter, Florida 33458, United States; ‡ Center for NeuroGenetics, University of Florida, Gainesville, Florida 32610, United States; § Department of Molecular Genetics & Microbiology, College of Medicine, University of Florida, Gainesville, Florida 32610, United States

## Abstract

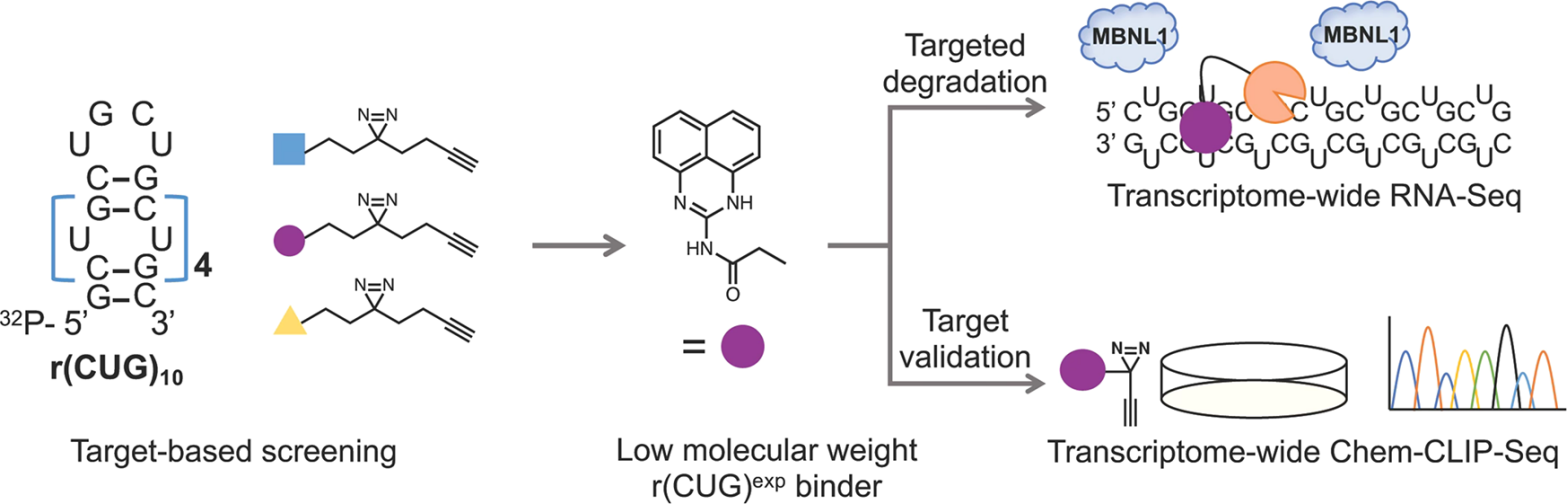

Myotonic dystrophy type 1 (DM1) is caused by a highly structured RNA repeat expansion, r(CUG)^exp^, harbored in the 3′ untranslated region (3′ UTR) of dystrophia myotonica protein kinase (*DMPK*) mRNA and drives disease through a gain-of-function mechanism. A panel of low-molecular-weight fragments capable of reacting with RNA upon UV irradiation was studied for cross-linking to r(CUG)^exp^*in vitro*, affording perimidin-2-amine diazirine (**1**) that bound to r(CUG)^exp^. The interactions between the small molecule and RNA were further studied by nuclear magnetic resonance (NMR) spectroscopy and molecular modeling. Binding of **1** in DM1 myotubes was profiled transcriptome-wide, identifying 12 transcripts including DMPK that were bound by **1**. Augmenting the functionality of **1** with cleaving capability created a chimeric degrader that specifically targets r(CUG)^exp^ for elimination. The degrader broadly improved DM1-associated defects as assessed by RNA-seq, while having limited effects on healthy myotubes. This study (i) provides a platform to investigate molecular recognition of ligands directly in disease-affected cells; (ii) illustrates that RNA degraders can be more specific than the binders from which they are derived; and (iii) suggests that repeating transcripts can be selectively degraded due to the presence of multiple ligand binding sites.

## Abstract 2

Myotonic dystrophy type 1 (DM1) is caused by a r(CUG) repeat expansion. We describe the development of a pipeline to identify small molecules that binds and eliminate the DM1-causing r(CUG)^exp^ in cells.

## Introduction

While only 1–2% of the human genome is translated into protein, ∼75% is transcribed into RNA.^1^ As RNA functions in biological processes and its deregulation triggers various pathological mechanisms, it is an important target for lead medicines or chemical probes.^2−4^ In many cases, RNA function depends on its folding and three-dimensional structure, which can be targeted by small molecule ligands to affect downstream biological pathways.^5,6^

One class of disease-causing RNAs is repeat expansions, which are known to cause >40 neurological and neuromuscular disorders.^7−11^ Myotonic dystrophy type 1 (DM1), the most common type of adult onset muscular dystrophy, is characterized by multisystemic symptoms including muscle weakness and myotonia.^12−14^ The molecular entity operating in DM1 is an r(CUG) repeat expansion [r(CUG)^exp^] harbored in the 3′ untranslated region (3′ UTR) of the dystrophia myotonia protein kinase (*DMPK*) mRNA.^15^ Pathogenicity is triggered by a conformational change in the RNA structure when repeat length exceeds a certain threshold (>50 repeating units), forming hairpins with repeating 1 × 1 nucleotide U/U internal loops. Expanded repeats have a pathological gain-of-function mechanism, sequestering various RNA binding protein (RBPs) including the pre-mRNA alternative splicing regulator muscleblind-like 1 (MBNL1).^16−19^ Sequestration of MBNL1 prevents its normal function, leading to defects in the alternative splicing of pre-mRNA substrates.^20,21^ Additionally, the repeat expansion and sequestered proteins form nuclear foci that impair the nucleocytoplasmic transport of *DMPK* mRNA.^22^

Here, we developed a streamlined platform for the identification of low-molecular-weight compounds that bind r(CUG)^exp^. This approach is broadly applicable across RNA targets and functions by identifying transcripts that interact selectively with small molecules, accomplished by screening a panel of low-molecular-weight fully functionalized fragments (FFFs). FFFs were first reported for studying the ligandability of proteins^23,24^ and RNA.^25,26^ The FFFs each contain a diazirine cross-linking module and alkyne tag, enabling a method named Chemical Cross-Linking and Isolation by Pull-Down (Chem-CLIP).^27^*In vitro* Chem-CLIP studies afforded a perimidin-2-amine diazirine (**1**) that specifically binds to r(CUG) repeats with nanomolar affinity.

This RNA–small molecule interaction was then further characterized by using a variety of biophysical methods. RNA sequencing (RNA-seq) analysis of targets enriched by Chem-CLIP in both DM1 patient-derived myotubes and WT myotubes from healthy donors identified all transcripts that directly interact with compound **1** in live cells. Indeed, **1** binds few transcripts in patient-derived cells and engages the target *DMPK* mRNA transcriptome-wide. Lastly, **1** was conjugated to a natural product to form a chimeric RNA cleaver that specifically eliminated r(CUG)^exp^ and improved DM1-associated cellular defects.

## Results and Discussion

### Identification of Compound 1 as a Low-Molecular-Weight Compound That Binds r(CUG)^exp^ Using *In Vitro* Chem-CLIP

**Figure 1:**
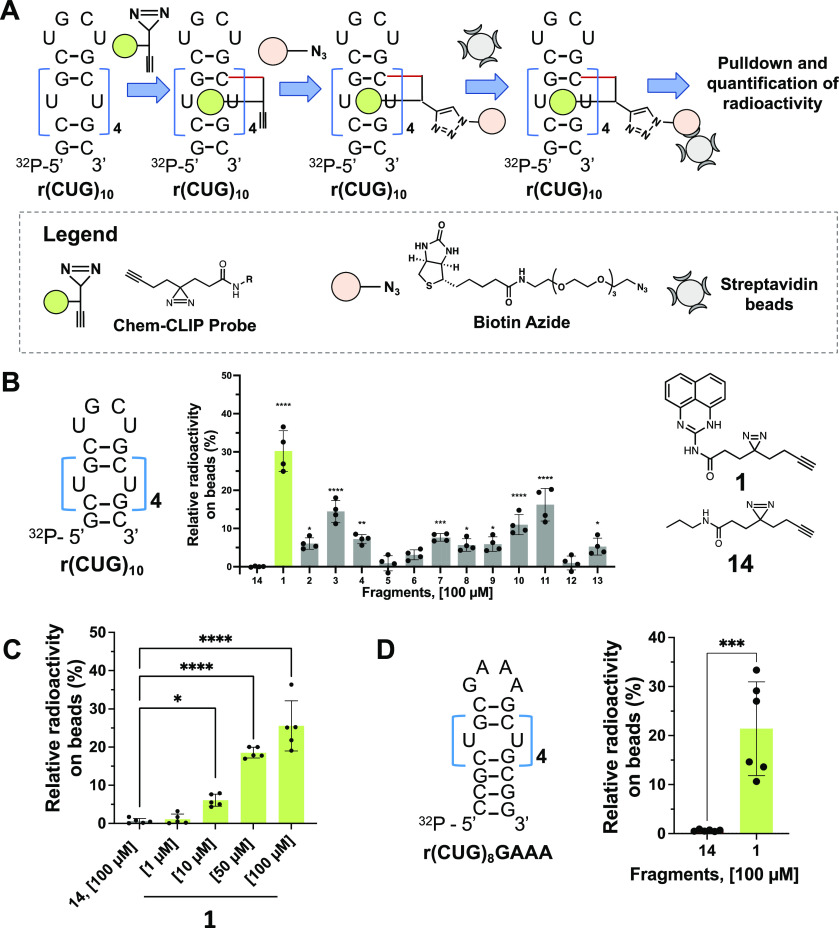
Screening of diazirine small molecule fragments. (A) Schematic of diazirine screening for r(CUG) repeat expansion binding. (B) Left: Structure of the radiolabeled r(CUG)_10_. Middle: *In vitro* Chem-CLIP of the 13 fragments tested at 100 μM showing **1** giving the highest pull-down of ^32^P-r(CUG)_10_ compared to **14** (*n* = 4). Right: Chemical structures of hit **1** and control probe, **14**. (C) Dose response of **1** by *in vitro* Chem-CLIP binding to ^32^P-r(CUG)_10_, (*n* = 5); *, *p* < 0.05; **, *p* < 0.01; ****, *p* < 0.0001; as determined by a one-way ANOVA with multiple comparisons. (D) Left: Structure of the radiolabeled r(CUG)_8_ presenting a GAAA hairpin loop. Right: *In vitro* Chem-CLIP confirming that compound **1** does not interact with the hairpin loop but binds to the U/U loops (*n* = 6); ***, *p* < 0.001; as determined by an unpaired *t* test. All data are reported as the mean ± SD.

A panel of 13 low-molecular-weight fully functionalized fragments (Figure S1A),^26^ were designed and synthesized based on their similarities to known RNA binders as shown by a Uniform Manifold Approximation and Projection (UMAP) analysis^28,29^ and comparison to the Inforna database (504 compounds).^30^ Indeed, the overlapping distribution of their chemical space confirms RNA-binding properties among these 13 fragments (Figure S1B). Each fragment, containing a diazirine cross-linking moiety and an alkyne handle for subsequent purification of the adducts, was screened for binding to r(CUG)_10_*in vitro* by Chem-CLIP.^27,31^ [Note, r(CUG)_10_ is a validated structural model of r(CUG)^exp^.^32−34^] After individually incubating each fragment (100 μM) with radiolabeled r(CUG)_10_, bound compounds were cross-linked to the RNA by photolysis, biotinylated by click chemistry,^35,36^ and pulled down with streptavidin beads (Figure 1A). Of the 13 fragments evaluated, compound **1** gave the highest enrichment (30 ± 5%, *p* < 0.0001) of radiolabeled r(CUG)_10_ compared to the control diazirine **14** lacking the RNA-binding module (Figure 1B). Additionally, **1** was evaluated for dose-dependently pulling down r(CUG)_10_, with a small yet statistically significant pull-down starting at 10 μM (6 ± 2%; *p* < 0.05), as compared to the control diazirine probe **14** (Figure 1C). At the highest dose tested, 100 μM, **1** pulled down 26 ± 7% of r(CUG)_10_ (*p* < 0.0001; Figure 1C). Here, the observed pull-down is a function of binding affinity, residence time, cross-linking efficiency, and the efficiency of the click reaction. While the efficiencies of click reactions with biotin-azide are usually >90%,^37^ upon photolysis of the diazirine, the resulting carbene can also be quenched by water, lowering the generation of covalent bonds with the target.^38^

As noted above, r(CUG) repeats fold into hairpin structures with a periodic array of 1 × 1 nucleotide U/U internal loops. To assess which structure fragment **1** binds, *in vitro* Chem-CLIP was performed using a construct with the same number of internal loops in r(CUG)_10_ but with a GAAA, rather than a UGCU, hairpin loop (Figure 1D). Interestingly, **1** enriched the RNA with the GAAA hairpin similarly to r(CUG)_10_ (studied at a single dose of 100 μM), indicating that **1** engages the 1 × 1 nucleotide U/U internal loops and not the UGCU hairpin (Figure 1D).

**Figure 2:**
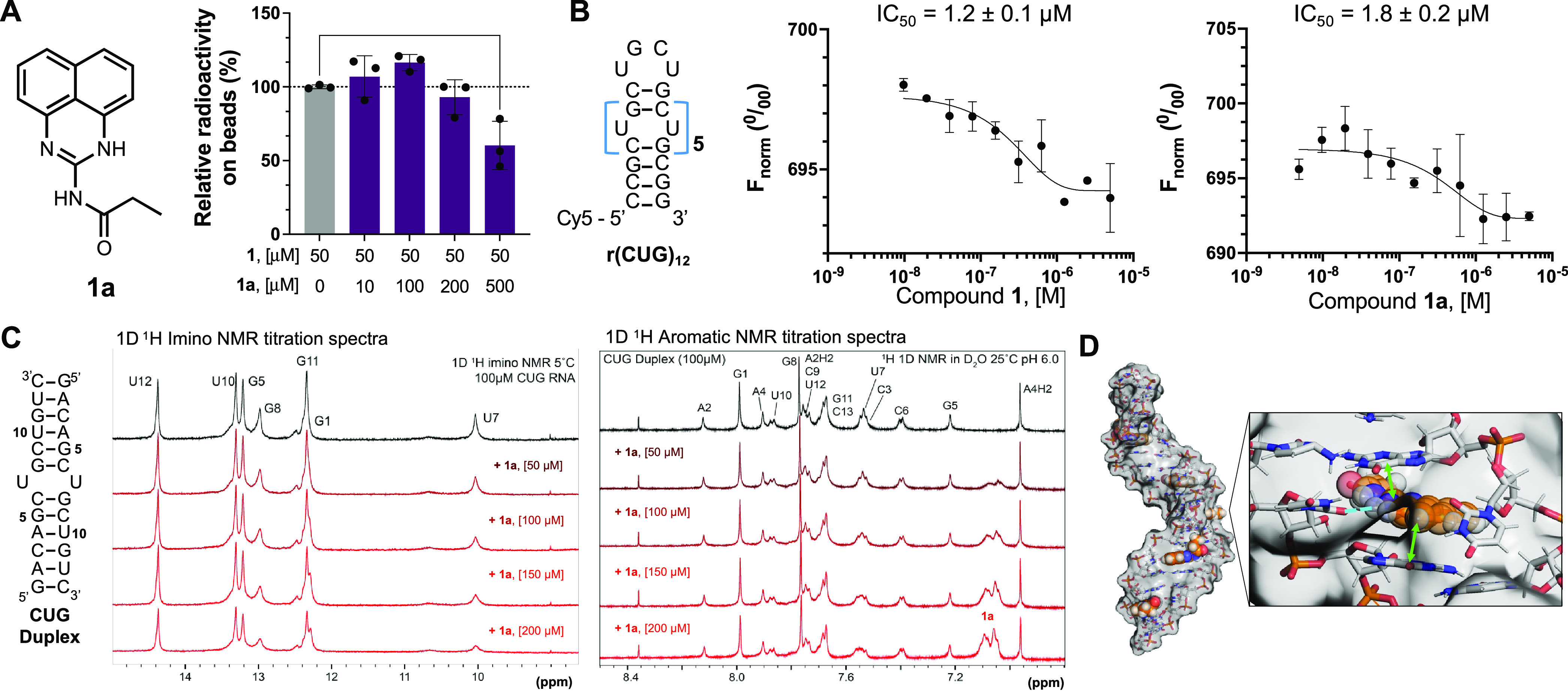
*In vitro* target engagement studies of a monomeric binder **1a**, to r(CUG)^exp^. (A) Left: Chemical structure of parent compound **1a**, lacking diazirine functionalization, replacing it with a propanamide group. Right: *In vitro* competitive Chem-CLIP experiment demonstrating competition between lead fragment **1** and the newly synthesized binder **1a**, (*n* = 3); **, *p* < 0.01; as determined by a one-way ANOVA with multiple comparisons. (B) Left: Structure of the Cy5 labeled r(CUG)_12_ used for MST binding assays. Right: Binding affinity (IC_50_) of compounds **1** and **1a** for Cy5-r(CUG)_12_, (*n* = 2). (C) Left: Duplex model of r(CUG) used for NMR studies. Right: 1D ^1^H imino and 1D ^1^H aromatic NMR titration spectra of the CUG duplex with **1a**. RNA assignments shown in black, with increasing additions of 50 μM compound shown as red spectra. (D) Left: r(CUG)_12_ hairpin model in complex with compound **1a** generated through MD simulations and free energy calculations; Right: compound **1a** in the binding pocket created by flipped out U in the U/U internal loop. Adopted orientation is stabilized by stacking interactions (green arrows) with neighboring bases and hydrogen bond (blue dotted line).

Given that diazirine fragment **1** had the highest enrichment and selectively engaged the 1 × 1 nucleotide U/U internal loops harbored in r(CUG)^exp^, we synthesized a perimidin-2-propionamide (**1a**; Figure 2A), where the propionamide replaces the diazirine cross-linker in **1**. To study if **1** and **1a** recognize the same site of r(CUG)^exp^, namely, the 1 × 1 nucleotide U/U internal loops, a Competitive Chem-CLIP (C-Chem-CLIP) experiment was completed wherein radiolabeled r(CUG)_10_ was coincubated with 50 μM of **1** and varying concentrations of **1a** (0 to 500 μM). Indeed, **1a** competed with the binding and cross-linking of **1**, as evidenced by the dose-dependent reduction of radioactively labeled r(CUG)_10_ pulled down by the fragment (Figure 2A). Thus, both molecules bind the same internal loops of r(CUG)_10_.

### Compounds 1 and 1a Bind to r(CUG) Repeats Selectively

The affinities of **1** and **1a** for Cy5-r(CUG)_12_ were measured by microscale thermophoresis (MST). Compounds **1** and **1a** bind the repeat with similar affinities, with IC_50_ values of 1.2 ± 0.1 μM and 1.8 ± 0.2 μM, respectively (Figure 2B). Specificity was explored by studying an RNA construct in which the 1 × 1 nucleotide U/U internal loops were replaced with base pairs, r(CAG)_7_-(CUG)_5_ (Figure S2A). No saturable binding was observed for **1** or **1a**, indicating specificity for the internal loops (Figure S2A). The stoichiometry of the binding of r(CUG)_12_ by **1a** was also assessed; r(CUG)_12_ folds into five 1 × 1 nucleotide U/U internal loops, each a potential binding site. The stoichiometry of the r(CUG)_12_–**1a** complex was 4.9 ± 0.7:1 (Figure S2B), indicating that each internal loop is bound by **1a**. To verify the binding observed by MST, we also completed a To-Pro-1 dye displacement assay using an RNA duplex that houses a single 5′CUG/3′GUC internal loop. To-Pro-1 binds to the RNA with a *K*_d_ = 31 ± 2 nM using a one-site binding model, affording *K*_d_ values of 1.2 ± 0.2 μM for **1** and 1.4 ± 0.3 μM for **1a** (Figure S2C). Notably, cross-linking was observed *in vitro* with as little as 10 μM of **1**. As noted above, not all binding events may lead to pull-down, influenced by the efficiency of the cross-linking (competition of the RNA and water for diazirine)^38^ and the click reaction. These and other factors help to explain the differences observed in binding measurements (MST and dye displacement) and Chem-CLIP studies.

### NMR Spectroscopy Studies, Docking, and MD Simulation Show Binding of the 1 × 1 Nucleotide U/U Internal Loops by **1a**

The binding of **1a** was further evaluated by NMR spectroscopy using a model RNA duplex containing a single 1 × 1 nucleotide U/U internal loop formed when r(CUG)^exp^ folds [5′-(GACAGCUGCUGUC)_2_-3′] (Figure 2C). In WaterLOGSY experiments,^39,40^ addition of the RNA to **1a** decreased the intensity of the fragment’s resonances at 6.5 ppm, consistent with binding (Figure S3). Furthermore, imino proton spectra (^1^H; H_2_O) of the RNA, which detects base pairing,^41−44^ showed perturbations and broadening of the peak at 10.0 ppm, corresponding to the 1 × 1 nucleotide U/U loop,^45^ upon addition of **1a** (Figure 2C). Spectra of the aromatic region (^1^H; D_2_O) also showed chemical shift perturbations upon addition of compound **1a** specific to the U/U loop, with signals decreasing for a neighboring base (C6H6) or upfield shifting (G8H8) (Figure 2C). Additionally, a new set of aromatic protons appear in the spectra at 7.05 and 7.1 ppm, consistent with repeated additions of **1a** (Figure 2C). Collectively, these studies demonstrate that **1a** binds to the 1 × 1 nucleotide U/U internal loop in r(CUG)^exp^.

To obtain a better understanding of the interactions between **1a** and internal loops present in r(CUG)^exp^, we applied a combination of docking and molecular dynamics (MD) simulations (Figure S4A–D). Various studies have shown that the U/U internal loops are inherently dynamic, forming different numbers of hydrogen bonds.^46−49^ The broadening of the peak corresponding to the U/U loop in the NMR studies described above suggest that the binding of **1a** causes structural changes within the U/U loops such that they no longer maintain stacking interactions with their closing base pairs.^50−52^ Docking and MD simulations show that these stacking interactions are replaced by the stacking of **1a** (Figures 2D and S4E). A stable hydrogen bond between **1a** and one of the uridines also stabilizes the bound state of the ligand (Figures 2D and S4E). Previous studies have shown that formation of one hydrogen bond between U/U mismatches is the most abundant hydrogen bonding structure.^49^ In essence, these hydrogen bonds and stacking interactions not only dictate the adopted pose of **1a** but also help to maintain the RNA’s structural features; that is, the interactions that dictate the structure of the U/U loop in the absence of small molecule are substituted with similar interactions formed by the binding of **1a**.

### The Expanded RNA Repeat Is Directly Engaged by 1 in DM1 Patient-Derived Myotubes

**Figure 3:**
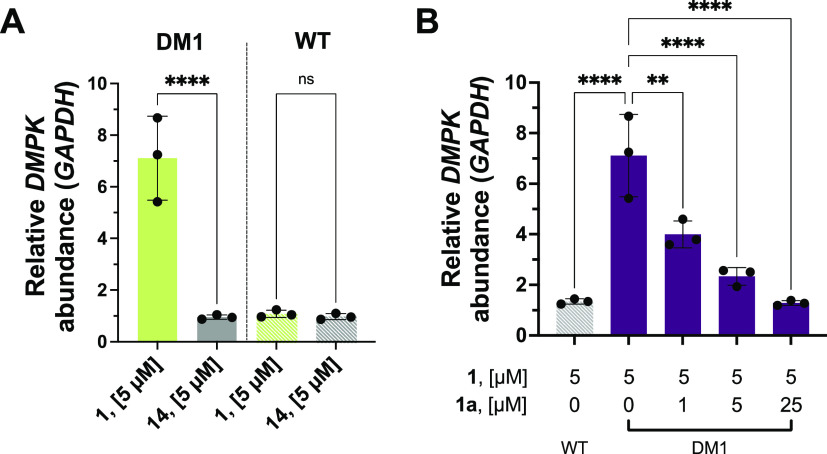
Target engagement of **1** and **1a** in DM1 patient-derived muscle cells. (A) Chem-CLIP pull-down of **1** in differentiated patient-derived DM1 and WT myotubes from healthy donors. Compound **1** significantly enriches the r(CUG)^exp^-containing *DMPK* gene selectively in DM1 cells (*n* = 3); ****, *p* < 0.0001; as determined by an unpaired *t* test with Welch’s correction. (B) Competitive Chem-CLIP experiment performed in patient-derived myotubes. Compounds **1** and **1a** compete for the same binding site in cells as observed by a decrease in the abundance of *DMPK* gene enriched by compound **1** upon addition of the binding monomer, **1a** (*n* = 3). Note: Enrichment with no competitor is the same on panels A and B to directly compare with Competitive-Chem-CLIP data; **, *p* < 0.01; ****, *p* < 0.0001; as determined by a one-way ANOVA with multiple comparisons. All data are reported as the mean ± SD.

We previously reported Chem-CLIP as a target validation method in cells, that is, incubating the probe with cells and then quantifying the enrichment of the RNA target after pull-down.^27,53,54^ Using the same method, DM1 patient-derived myotubes^55^ were treated with 5 μM of **1** and bound targets were cross-linked by irradiation with UV light and bound RNAs were pulled down with disulfide azide agarose beads. Target engagement was quantified by calculating the enrichment of RNA abundance in the lysate before and after pull-down, as determined by RT-qPCR. Indeed, **1** significantly enriched *DMPK* mRNA (which harbors r(CUG)^exp^) by ∼7-fold (*p* < 0.0001; Figure 3A), validating the target of the fragment in cells. In comparison, the control probe **14**, which lacks the RNA-binding module, showed no significant enrichment of *DMPK* mRNA (Figure 3A). C-Chem-CLIP studies,^56^ performed by pretreating DM1 patient-derived myotubes with **1a** (1–25 μM) for 1 h followed by treatment with 5 μM of **1** (overnight), showed a dose-dependent reduction of the enrichment of *DMPK* mRNA, confirming that **1** and **1a** engage the same binding site in cells (Figure 3B). To confirm that enrichment of *DMPK* mRNA is dependent on the presence of r(CUG)^exp^, myotubes derived from a healthy donor were also treated with 5 μM of **1**, and no significant enrichment of *DMPK* mRNA was observed (Figure 3A). Additionally, we assessed the propensity of compound **1** to bind other biomolecules and found that no significant enrichment of protein or DNA was observed compared to the control compound **14**, suggesting preferential binding for its RNA target in cells (Figure S5).

### Transcriptome-Wide Binding of 1, as Determined by Chem-CLIP-Seq

**Figure 4:**
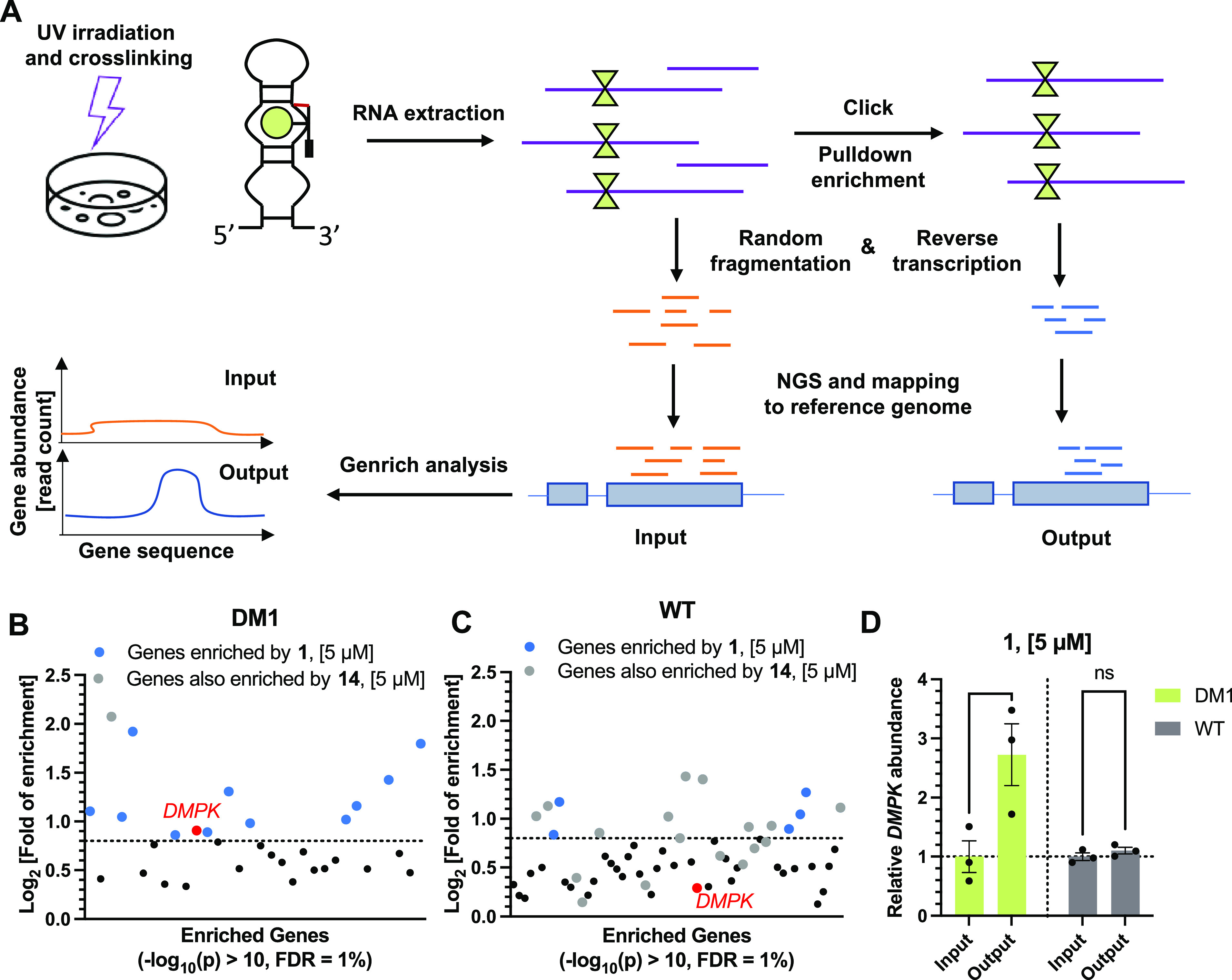
Target engagement by Chem-CLIP-Seq analysis of compound **1** in differentiated myotubes. (A) Scheme of Chem-CLIP-Seq methodology used to identify gene enrichment. (B) Chem-CLIP-Seq analysis in patient-derived DM1 myotubes identifying 12 genes (Log_2_ > 0.8) enriched by **1** transcriptome-wide. (C) Chem-CLIP-Seq analysis in WT myotubes from healthy donors identifying 5 genes (Log_2_ > 0.8) enriched by compound **1** transcriptome wide. (D) Chem-CLIP-Seq analysis showing enrichment of *DMPK* near the r(CUG) repeat in patient-derived DM1 myotubes and WT myotubes from healthy donors treated with 5 μM of compound **1** (*n* = 3); **, *p* < 0.01; as determined by a two-way ANOVA with multiple comparisons. All data are reported as the mean ± SD.

We next studied the transcriptome-wide selectivity of **1** by integrating Chem-CLIP with RNA-seq, or Chem-CLIP-Seq.^26,27,57^ Following treatment of patient-derived myotubes or myotubes from a healthy donor with **1** (5 μM) and cross-linking, RNA was isolated and then pulled down. After eluting the bound RNAs from the beads, the samples were fragmented and subjected to RNA-seq analysis with random primers, thereby identifying all transcript fragments enriched by **1** (Figure 4A). To measure enrichment, RNA that was not subjected to the pull-down steps was also fragmented and analyzed by RNA-seq. The analogous experiments were completed for control diazirine probe **14** (Figure 1B), which lacks an RNA-binding module. All RNA-seq data sets were aligned to the Hg38 reference genome^58^ (which does not contain an RNA repeat expansion). Collectively, this method aims to define cellular target engagement and global selectivity of **1** by comparison of disease-affected and healthy cells (target agnostic), as opposed to enrichment of a specific target (target biased).

First, to assess global selectivity, transcripts significantly enriched (−Log_10_(*p*) > 10) by **1** were identified by using the software package Genrich, a publicly available program used for genomic enrichment assays.^26,59^ Genrich uses a null model with a log-normal distribution to calculate *p* values of enrichment (defined as the ratio of reads after pull-down divided by the reads before pull-down) at each nucleotide position across the entire human genome. The adjacent nucleotides passing a cutoff of −Log_10_(*p*) > 10 were compiled together to afford the regions of enrichment, and these regions were further triaged with additional filters: (i) a minimum area under curve (AUC) of 200; (ii) a length range of 400–1000 nucleotides, the length fragment observed by bioanalyzer analysis after pull-down (Figure S6); (iii) a minimum read count of 10; and (iv) consistent enrichment in all 3 replicates with a minimum Log_2_ fold enrichment of 0.8, thereby removing low-confidence enrichments. It should be noted that occasionally multiple regions of enrichment were identified for the same transcript, in which case we summed the total reads received for that transcript, normalized to total reads, when calculating enrichment.

This sequencing analysis revealed regions from 12 transcripts including *DMPK* as significantly enriched in DM1 patient-derived myotubes by **1** and not by the control probe **14** (Figure 4B and Table S2). The region identified within *DMPK* was ∼1000 nucleotides upstream of r(CUG)^exp^ in the mRNA. Interestingly, none of the other 11 regions include a r(CUG) repeat >5 units in their sequence. A similar analysis in myotubes from a healthy donor identified 5 genes as significantly enriched (Figure 4C and Table S3), which do not overlap with the transcripts bound by **1** in DM1 myotubes. That is, *DMPK* was only enriched in DM1 patient-derived myotubes (log_2_ = 0.91) and not in myotubes from a healthy donor (log_2_ = 0.29), supporting the selectivity of the small molecule for the mutant allele. The 16 other genes bound by **1** (11 in DM1 and 5 in WT myotubes) are not differentially expressed in DM1 and WT myotubes and are therefore not implicated in DM1 pathology. Of note, the 2600 r(CUG) repeats found only in DM1 cells potentially form about 1000 1 × 1 UU internal loops, influencing the target occupancy of **1** transcriptome-wide. Further, the two cell lines are not isogenic, thus confounding a direct comparison. Although fragments are generally thought to be promiscuous, fragment-like **1** (MW = 331) contains distinct physicochemical features such as densely arranged H-bond donors/acceptors that may underlie the somewhat higher selectivity than might be anticipated *prima facie*.

Direct visualization of the RNA-seq track in the r(CUG)^exp^ region of *DMPK* showed an overall increase of the reads in the DM1 myotubes after pull-down (“Output”) while a decrease of the reads after pull-down from WT myotubes was observed, each compared to their samples prior to pull-down (“Input”) (Figure S7A). The same RNA-seq track visualization of control probe **14**-treated cells shows an overall decrease of the number of reads after pull-down (Figure S7B). Notably, the repeating nature and GC-content of r(CUG)^exp^ presents a challenge in RNA sequencing.^60,61^ Along with alignment to a reference genome that does not contain a r(CUG)^exp^ (reads containing solely the repeat are unable to be aligned; the DM1 myotubes studied herein have 2600 repeats), the observed enrichment is likely an underestimate.

While the region of enrichment identified by Genrich did not include the r(CUG)^exp^ sequence, as it did not pass our filters due to the low AUC (area under the curve), pure repeats are often difficult to amplify and clone into RNA-seq libraries. When we quantified enrichment flanking the r(CUG)^exp^ sequence (500 nt window including the r(CUG) repeat region of the Hg38 reference genome), we observed a ∼3-fold enrichment (Figures 4D and S7A). Quantification of the same region in WT myotubes treated with **1** showed no increase in read count of *DMPK*, further supporting the selectivity of the small molecule for the mutant allele (Figures 4D and S7A). In parallel, the control diazirine probe **14** was tested to ensure that target engagement was specific to **1** and not due to the cross-linking moiety itself. No change in *DMPK* abundance after pull-down by **14** was observed in either DM1 or WT myotubes (Figure S7B–C). These results demonstrate global selectivity of **1** and confirmed target engagement of the mutant *DMPK* allele.

### Synthesis of a Degrader, 1b, That Cleaves r(CUG)^exp^*In Vitro*

**Figure 5:**
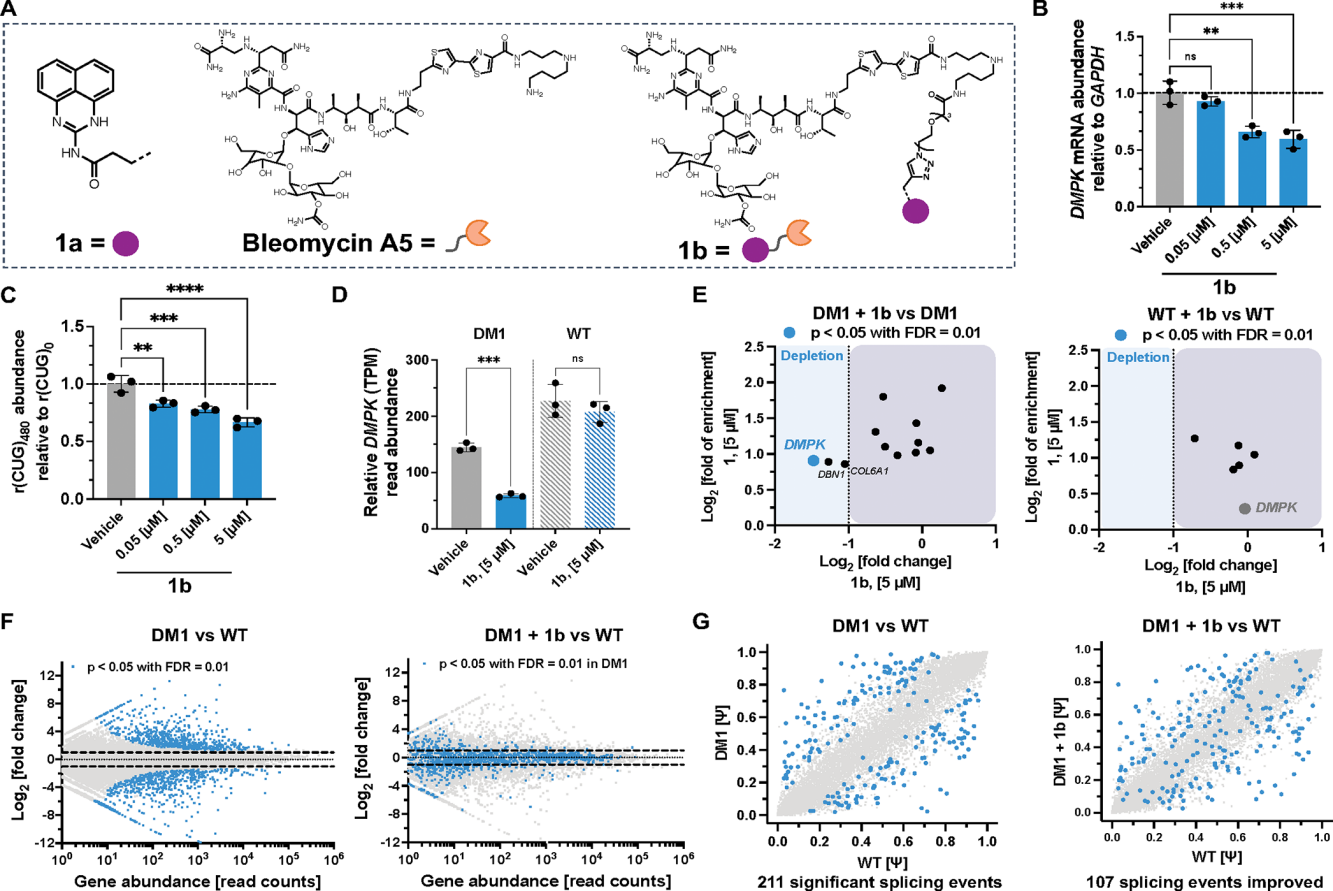
Design and transcriptome-wide assessment of monomeric cleaver, **1b** that cleaves toxic r(CUG)^exp^ in DM1 patient-derived myotubes. (A) Structure of compound **1b**, capable of directed RNA cleavage. (B) Effect of **1b** on *DMPK* abundance, which harbors r(CUG)^exp^, in patient-derived DM1 myotubes as determined by RT-qPCR (*n* = 3). (C) Effect of **1b** in HeLa480 cells stably expressing r(CUG)_480_ and r(CUG)_0_ (*n* = 3). (D) RNA-seq analysis of DMPK abundance as measured by relative transcript reads in patient-derived DM1 myotubes and WT myotubes from healthy donors treated with 5 μM of **1b** (*n* = 3). (E) Left: RNA-Seq analysis of compound **1b** (*X*-axis) and Chem-CLIP-Seq analysis of compound **1** (*Y*-axis) showing selective downregulation of *DMPK* among genes enriched by **1** in patient-derived DM1 myotubes. Right: RNA-Seq analysis of compound **1b** (*X*-axis) and Chem-CLIP-Seq analysis of compound **1** (*Y*-axis) in WT myotubes from healthy donors showing no effects of compound **1b** on any enriched genes including *DMPK* by compound **1** indicating no detectable off-target effects. (F) Left: Gene expression RNA-seq analysis of patient-derived DM1 myotubes compared to WT myotubes from healthy donors both treated with DMSO. Highlighted in blue are significant (*p* < 0.05) genes that are dysregulated in DM1 myotubes (*n* = 3). Right: Gene expression RNA-seq analysis of patient-derived DM1 myotubes once treated with 5 μM of **1b** compared to WT myotubes from healthy donors treated with DMSO. Highlighted in blue are genes that were significantly (*p* < 0.05) dysregulated when DM1 myotubes were treated with DMSO (*n* = 3). (G) Left: Splicing events in patient-derived DM1 vs WT myotubes from healthy donors treated with vehicle and Right: patient-derived DM1 myotubes treated with 5 μM of **1b** vs WT myotubes from healthy donors treated with vehicle. The *X*-axis denotes Ψ in WT myotubes from healthy donors and the *Y*-axis denotes Ψ in patient-derived DM1 myotubes. Blue spots indicate events that are significantly mis-spliced in DM1 cells. Genes that shift toward the diagonal indicate rescue upon treatment with compound **1b**. **, *p* < 0.01; ***, *p* < 0.001; ****, *p* < 0.0001; as determined by a one-way ANOVA with multiple comparisons. All data are reported as the mean ± SD.

We previously demonstrated that functionalization of small molecules with cleavage moieties can direct site-specific cleavage of RNA,^7,62^ increasing compound potency and rescuing molecular defects in patient-derived cells and *in vivo* models.^7^ To this end, **1a** was functionalized with Bleomycin A5, affording **1b** (Figure 5A), as a means to elicit targeted degradation of r(CUG)^exp^ and rescue DM1-associated defects. Bleomycin is a natural product commonly used for the treatment of cancer through DNA cleavage by metal-ion and oxidative mechanisms.^63−65^ Studies from both the Hecht laboratory as well as our own have demonstrated that Bleomycin cleaves RNA.^66−68^ Further, linkage to an RNA-binding small molecule or oligonucleotide affords programmable control over its cellular targets while eliminating undesired DNA cleavage.^22,62,67,69^ Importantly, attachment of an RNA-targeting small molecule to the terminal primary amine of Bleomycin provides selective RNA cleavers^7,62^ by removing a positive charge critical for DNA binding interactions, as elucidated by mechanistic and structural studies.^65,70−72^

Briefly, an alkyne handle was attached to the free amine of the perimidin-2-amine (Supporting Information) which was subsequently clicked to a dual-functionalized PEG_3_ linker (azide and carboxylic acid). This synthetic strategy was employed to avoid cyclization with the aromatic secondary amine and to improve solubility of the intermediate. The free amine of Bleomycin A5 was coupled to the carboxylic acid intermediate to afford **1b** (Figure 5A).

Conjugation of Bleomycin to the r(CUG)^exp^-targeting small molecule minimally affected molecular recognition of the target as **1** (IC_50_ of 1.2 ± 0.1 μM), **1a** (IC_50_ of 1.8 ± 0.2 μM), and **1b** (IC_50_ of 2.1 ± 0.1 μM) all bind similarly to r(CUG)_12_ as determined by MST (Figures 2B and S8A) and no saturable binding of **1b** to the control, fully paired r(CAG)_7_-(CUG)_5_ was observed (Figure S8B). The affinity of **1b** for the RNA duplex harboring a singular 5′CUG/3′GUC binding site was measured using the To-PRO-1 dye displacement (competition) assay described above, giving a *K*_d_ of 3.3 ± 0.6 μM (Figure S8C), 2–3-fold lower affinity than **1** and **1a**. This could be due to various factors such as the addition of the large bleomycin cleavage modality or its distance from the RNA-binding module. [Note, binding affinity measurements were completed in the absence of Fe^2+^, which is required for cleavage.^65^]

We next sought to validate target engagement of **1b***in vitro* using C-Chem-CLIP studies. Radiolabeled r(CUG)_10_ was coincubated with 50 μM of Chem-CLIP probe **1** and varying concentrations of **1b** (0–500 μM) in a solution lacking Fe^2+^. With increasing concentrations of **1b**, a dose-dependent decrease in the percent of r(CUG)_10_ pulled down by **1** was observed (Figure S8D), indicating that **1b** indeed binds the same site within the repeats as **1**.

The *in vitro* cleavage of r(CUG) repeats by **1b** was further evaluated by gel electrophoresis. Dose dependent cleavage of radiolabeled r(CUG)_10_ was observed upon incubation with increasing concentrations of **1b** (0–10 μM; in the presence of Fe^2+^), with 47 ± 14% of the RNA cleaved at 2.5 μM and 76 ± 14% cleaved at 10 μM (Figure S8E). Importantly, no statistically significant cleavage of r(CUG)_10_ was observed with the parent compound **1a** (<7% at 10 μM concentration; Figure S8E). Analysis of the nucleotides at which cleavage by **1b** occurs revealed that the primary sites of cleavage are the 3′ GC base pairs that close the 1 × 1 internal U/U loops (Figure S8E). Collectively, these data confirm that compound **1b** binds the 1 × 1 nucleotide internal U/U loops formed by the expanded r(CUG) repeat, as determined from C-Chem-CLIP studies, and elicits site-specific cleavage of the RNA *in vitro*.

### Compound 1b Targets Pathogenic r(CUG)^exp^ in DM1 Patient-Derived Myotubes and Alleviates DM1-Associated Defects

Next, the ability of **1b** to cleave r(CUG)^exp^ and improve DM1-associated defects in cells was assessed. In DM1 patient-derived myotubes,^55^ 48 h treatment with **1b** resulted in a dose dependent decrease of *DMPK* transcript levels, with 41 ± 8% reduction observed at 5 μM dose, as determined by RT-qPCR (Figure 5B). Importantly, this decrease is specific to **1b** as the parent compound **1a**, which lacks the Bleomycin cleavage module, does not affect *DMPK* transcript levels (Figure S9A). [Note, the qPCR primers used in these studies do not distinguish between mutant and WT alleles. Previous studies have shown that ∼50–70% of *DMPK* transcript in DM1 myotubes harbor r(CUG)^exp^.^73^] Additionally, no toxicity was observed in WT myotubes upon treatment with **1b** (Figure S9B). Importantly, the observed decrease in *DMPK* abundance was selective for r(CUG)^exp^ as *DMPK* levels in WT myotubes, which express r(CUG)_20_, were unaffected by treatment with **1b** (Figure S9C).

As the mutant and WT alleles in DM1 myotubes cannot be differentiated by qPCR primers, selectivity was assessed in HeLa cells that stably express r(CUG)_480_ and r(CUG)_0_ that can be distinguished by RT-qPCR.^74^ Treatment with **1b** dose dependently reduced r(CUG)_480_ but not r(CUG)_0_, confirming the selective degradation of the mutant allele (Figure 5C). As an orthogonal approach to measure cleavage selectivity, the effect of **1b** on the abundance of several known transcripts with short, nonpathogenic r(CUG) repeats expressed in DM1 myotubes was assessed by RT-qPCR. Previous studies have shown that these short repeats either do not form 1 × 1 nucleotide U/U internal loops present in r(CUG)^exp^ or are unstructured.^7^ Indeed, **1b** had no effect on any of the transcripts with short repeats (Figure S9D), further supporting a selective mechanism of action, cleavage of r(CUG)^exp^ by recognition of its 1 × 1 nucleotide U/U internal loops.

To confirm that conjugation of Bleomycin A5’s terminal amine to the small molecule ablates the ability to damage and cleave DNA,^7,62^ we studied whether **1b** induced DNA damage in DM1 myotubes via quantification of γ-H2AX foci, formed in response to DNA double-strand breaks,^75^ by immunostaining. After treatment with 5 μM of **1b** for 48 h, no significant increase of γ-H2AX foci was observed, in contrast to Bleomycin A5, which caused a significant accumulation of γ-H2AX foci per nuclei (Figure S10).

### RNA-seq Analysis Demonstrates That 1b Broadly Improves DM1-Associated Defects in Patient-Derived Myotubes

To study the effects of **1b** comprehensively in patient-derived cells, transcriptome-wide analysis was performed on total RNA harvested from differentiated DM1 and WT myotubes. As expected, a significant decrease in *DMPK* abundance was observed upon treatment of DM1 myotubes with 5 μM of **1b**, as determined by the read count (transcripts per million; TPM) mapped to *DMPK* compared to vehicle-treated controls (59 ± 4 TPM in **1b**-treated myotubes vs 145 ± 7 TPM in vehicle treated; *p* < 0.001; Figure 5D). Importantly, this decrease was specific to disease as WT myotubes showed no change in *DMPK* abundance upon **1b**-treatment, to further support that the degrader is allele selective (Figure 5D).

Further exploring the selectivity of **1b**, we evaluated the effect of **1b** (i) on transcripts pulled down by **1** in Chem-CLIP studies completed in DM1 or WT myotubes; (ii) on transcripts containing short, nonpathogenic r(CUG) repeats; and (iii) on transcripts that encode proteins involved in the DNA damage response pathway. In DM1 myotubes, 12 transcripts were pulled down by **1** (Figure 4B). Of these, three transcripts were downregulated by more than 2-fold upon treatment with **1b**, *DBN1* (*p* = 0.22, Log_2_ = −1.27), *COL6A1* (*p* = 1, Log_2_ = −1.05), and *DMPK* (*p* = 0.00008, Log_2_ = −1.47) where only the depletion of *DMPK* was statistically significant (Figure 5E). The complete RNA-seq data set is publicly available on Mendeley data. None of the transcripts pulled down by **1** in WT myotubes (Figure 4C) were affected (*p* < 0.05, Log_2_ > −1) by **1b**-treament (Figures 5E and S11A). Additionally, RNA-seq analysis of genes containing short nonpathogenic r(CUG) repeats expressed in DM1 myotubes and studied herein by RT-qPCR (Figure S10D) confirmed that there was no significant decrease in their abundance upon treatment with **1b** (Figure S11B). Finally, no changes were observed in the abundance of 22 transcripts that encode proteins involved in the DNA damage pathway upon treatment of WT myotubes with 5 μM of **1b** (Figure S11C),^76^ in agreement with γ-H2AX foci imaging studies (Figure S10).

The expression of r(CUG)^exp^ causes many changes transcriptome-wide in DM1-affected cells. Comparison of untreated DM1 and WT myotubes revealed that 1319 genes are deregulated in DM1 cells (abundance may be increased or decreased with *p* < 0.05; Figure 5F). Upon treatment with **1b** (5 μM), 98% of deregulated genes were shifted toward WT levels, and the abundance in treated DM1 myotubes is no longer statistically different (*p* > 0.05) (Figures 5F and S12A). Furthermore, transcriptome-wide analysis of WT myotubes treated with **1b** did not show statistically significant (*p* < 0.05) changes in the expression of any gene, including *DMPK*, highlighting the selectivity of compound **1b** (Figure S12B).

As aforementioned, the alternative splicing of transcripts controlled by MBNL1 are deregulated in DM1 due to its sequestration and hence inactivation by r(CUG)^exp^.^20,21^ We therefore analyzed the RNA-seq data to assess rescue of MBNL1-regulated splicing events by **1b**. When comparing untreated DM1 and WT myotubes, 211 splicing events were identified as significantly (*p* < 0.05) misregulated (Figure 5G). Upon treatment with 5 μM of **1b**, 107 of the splicing events (51%) are shifted toward WT patterns and are no longer statistically different (*p* > 0.05) (Figure 5G).

Finally, the rescue of splicing defects observed in our transcriptome-wide analysis suggests that some portion of MBNL1 has been freed from sequestration by r(CUG)^exp^ in nuclear foci.^77^ We therefore used confocal microscopy to quantify the number of MBNL1- and r(CUG)^exp^-positive foci in DM1 myotubes, by immunohistochemistry and RNA fluorescence *in situ* hybridization (FISH), respectively, with and without treatment with **1b**. Treatment with compound **1b** reduced the number of r(CUG)^exp^–MBNL1 nuclear foci at all concentrations tested with the 5 μM dose decreasing the average number of RNA foci per nuclei from 4.1 ± 0.2 to 2.6 ± 0.1 compared to vehicle treated samples (*p* < 0.0001; Figure S13).

Collectively, these results show that converting a small molecule into a Bleomycin-conjugated degrader can confer potent and selective cleavage of an RNA target, rescuing downstream disease pathways in patient-derived muscle cells.

## Conclusions

In this report, we describe an orthogonal approach to identify RNA-binding small molecules *in vitro* through screening of a panel of fully functionalized fragments. These studies demonstrate that a fragment-based screening strategy can be employed to identify low-molecular-weight molecules that selectively engage a disease-causing RNA. Furthermore, conjugation of the identified fragment with a Bleomycin warhead afforded targeted degradation of r(CUG)^exp^ by recognition of its 1 × 1 nucleotide U/U internal loops in cells, eliciting rescue of various molecular defects associated with disease pathology including reduction of nuclear RNA foci and rescue of aberrant splicing events in patient-derived DM1 myotubes. Notably, previous data suggests that >40–60% cleavage of the toxic repeat can lead to improvement of mis-splicing events in tissue as well as reduction of myotonia in HSA^LR^ mice,^7,78−80^ suggesting potential therapeutic benefits of **1b***in vivo.* Through RNA sequencing analysis we observe that functionalization of the fragment leads afford bioactivity as well as improves selectivity (Figure 5F). Collectively, these data support that potent and selective RNA-targeting small molecules can be discovered from a simple fragment-based screen and augmented with functionality via cleavage.

Despite the challenging nature of designing and discovering novel and selective RNA binders,^2,4,81^ various strategies have been successfully implemented to afford bioactive molecules, including structure-based and sequence-based design methods.^30,82,83^ For example, a small molecule with a mixed mode of action (dual-targeting DNA and RNA) was designed to bind 1 × 1 nucleotide U/U internal loops via a Janus-Wedge interaction informed by an X-ray crystal structure of short r(CUG) repeats.^84^ A follow-up on this strategy was reported recently, and although the compounds modestly reduced the number of nuclear foci, they had no effect on alternative splicing defects.^85^ A biochemical assay that studied displacement of MBNL1 from r(CUG)^exp^*in vitro* afforded small molecules with activity in cells. Of note, those studies identified small molecules that inhibited the r(CUG)^exp^-MBNL1 interaction by binding both the RNA and the protein.^86^

A previous small molecule reported to selectively target r(CUG)^exp^ employed sequence-based design, modular assembly to target two U/U loop simultaneously, and its conjugation to bleomycin.^7^ This molecule, dubbed Cugamycin (Figure S14), was able to improve DM1-associated defects broadly and specifically in cells and a mouse model with no detectable off-targets.^7^ Herein, **1b**, which targets a single 1 × 1 nucleotide U/U internal loop can target r(CUG)^exp^ and become a specific modulator of DM1 dysfunction when converted into a degrader. The compound reduced *DMPK* transcript abundance to a similar extent as Cugamycin in DM1 patient-derived myotubes (∼45% vs ∼55% at 5 μM dosage) and similarly rescued DM1 pre-mRNA splicing defects, while possessing a significantly lower molecular weight (Figure S14). Therefore, there is potential for compounds to dramatically affect the biology of repeating transcripts by using compounds that target a singular repeating unit in a toxic RNA and improve function. It will be interesting to test if these observations are general to other repeating transcripts that cause diseases via gain of function such as c9ALS/FTD and Huntington’s disease, for example.

The work described here may be used as a benchmark to purposefully affect RNA-mediated pathways as we show that RNA can be efficiently targeted to improve disease-associated defects. There are many ways to modulate RNA function through diverse mode of actions, and while the field of RNA chemical biology expands, there is a need to also expand the strategies to identify lead molecules.

## Data Availability

The results of Chem-CLIP-Seq and RNA-Seq analysis were deposited in Mendeley Data (DOI: 10.17632/k44jpz492s.1).

## Supporting Information Available

Figures S1–S15 and tables S1–S5, experimental methods, and compound characterization (PDF)
