# Supplementary material for: Transcriptome-Wide Studies of RNA-Targeted Small Molecules Provide a Simple and Selective r(CUG)exp Degrader in Myotonic Dystrophy
Source: ACS Cent Sci. 2023 Jun 26;9(7):1342–53. doi: 10.1021/acscentsci.2c01223 (PMC10375898; doi:10.1021/acscentsci.2c01223)
Supplement: Supplementary file 1 — oc2c01223_si_001.pdf [file oc2c01223_si_001.pdf]

Supporting Information for:

Transcriptome-wide studies of RNA-targeted small molecules provide a simple and selective r(CUG)^{exp} degrader in myotonic dystrophy

Quentin M. R. Gibaut[†1], Jessica A. Bush[†1], Yuquan Tong[1], Jared T. Baisden[1], Amirhossein Taghavi[1], Hailey Olafson[2,3], Xiyuan Yao[1], Jessica L. Childs-Disney[1], Eric T. Wang[2,3], and Matthew D. Disney[*1]

[1]The Department of Chemistry, The Scripps Research Institute and UF Scripps Biomedical Research, Jupiter, FL 33458, United States

[2]Center for NeuroGenetics, University of Florida, Gainesville, FL 32610, United States

[3]Department of Molecular Genetics & Microbiology, College of Medicine, University of Florida, Gainesville, FL 32610, United States

[†]Q.M.R.G. and J.A.B. contributed equally to this work

*Author to whom correspondence should be addressed; Email: disney@scripps.edu

Supporting Information Figures

A

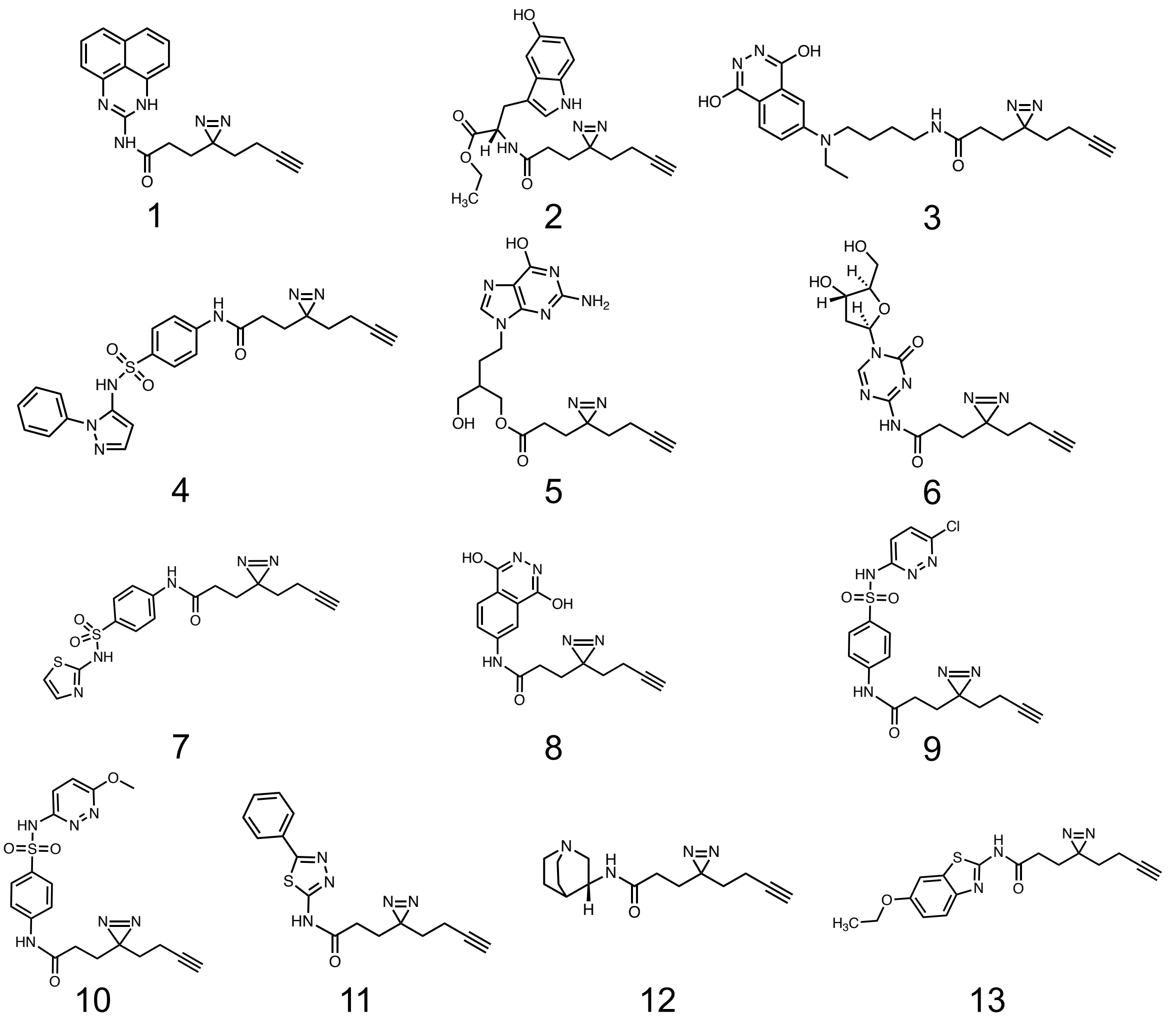

B

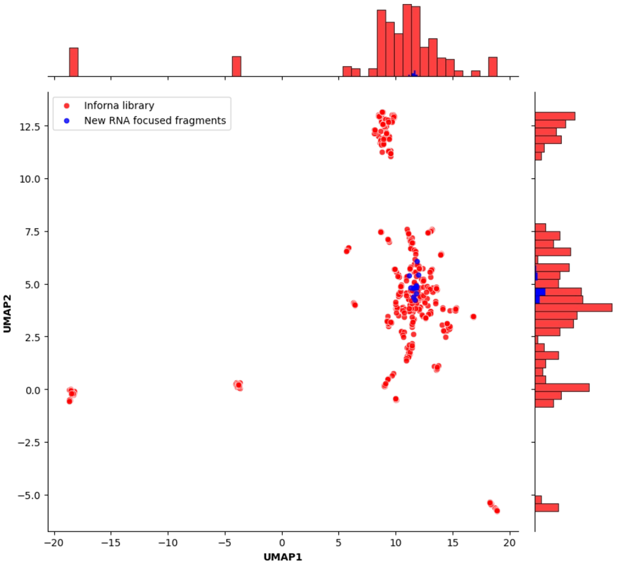

Figure S1. Design and synthesis of 13 RNA-focused fragments. A) Chemical structure of the 13 RNA-focused fragments.[1] B) Uniform Manifold Approximation and Projection (UMAP) showing the chemical space coverage of the binding module of the 13 fragments (**blue**) and the Inforna library (**red**). Morgan fingerprints (2,048-bit binary data, radius 3) were embedded into two-dimensions as UMAP1 and UMAP2.

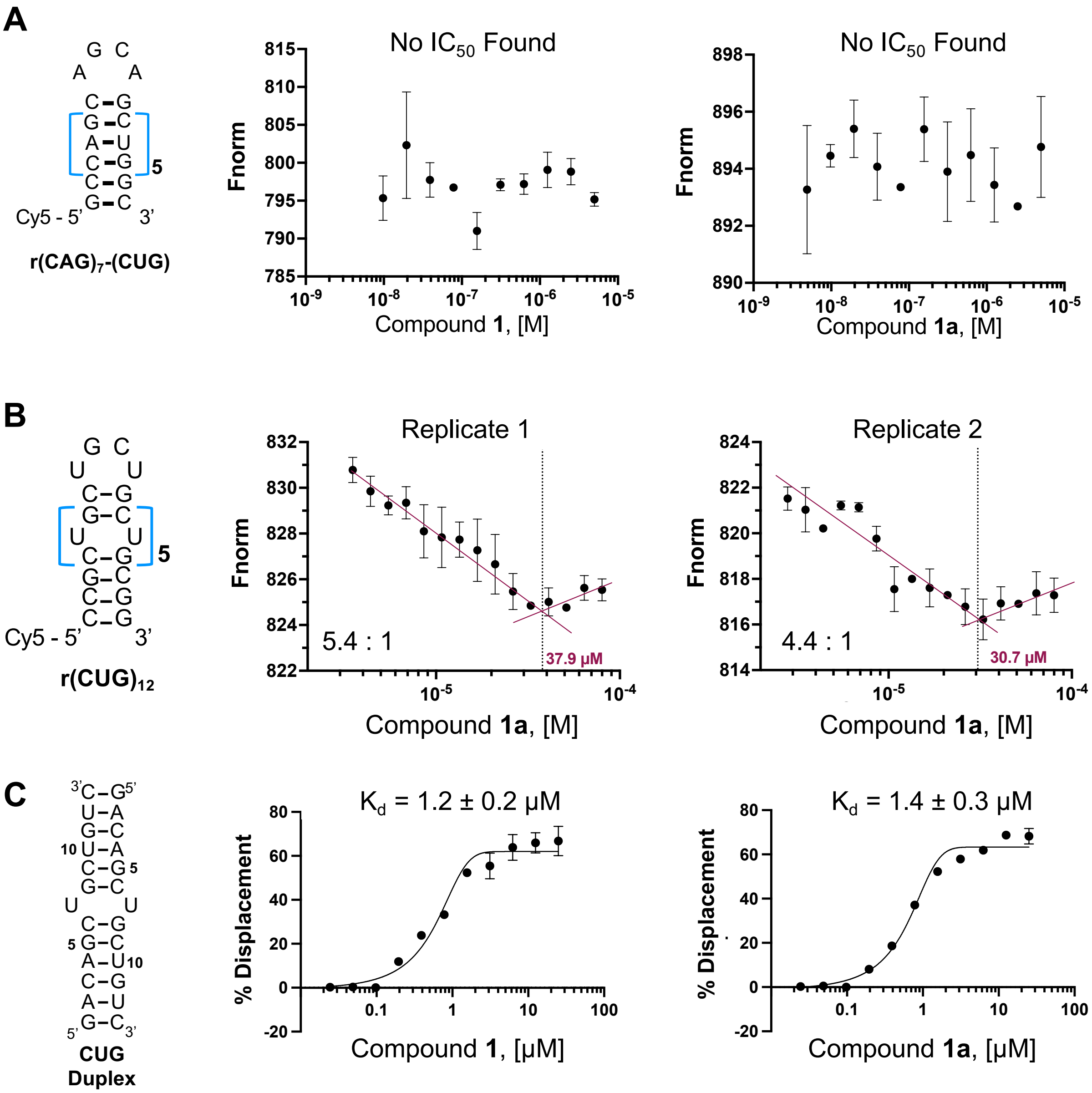

Figure S2. Binding affinities and stichometry of 1 and 1a for a base-paired control RNA, as determined by microscale thermophoresis (MST). A) Structure of the Cy5 labeled base-pair control used for binding measurements, r(CAG)$_7$-(CUG)$_5$ and MST analyses of the binding of **1** and **1a** to the base paired control RNA (n = 2). B) Structure of the 5'-Cy5 labeled RNA construct used to measure stoichiometry of the r(CUG)$_{12}$-**1a** complex by MST. Binding of **1a** displays saturated binding to r(CUG)$_{12}$ at 37.9 µM for replicate 1 and 30.7 µM for replicate 2 in the presence of 5.5 µM of Cy5-r(CUG)$_{12}$, affording an average stoichiometry 4.9 ± 0.7:1 and indicating occupancy of each 1×1 U/U internal loop. C) Structure of r(CUG) duplex used for affinity measurements by To-PRO-1 dye displacement and representative a binding curve for compounds **1** and **1a** used to K_d. Error is reported as SD.

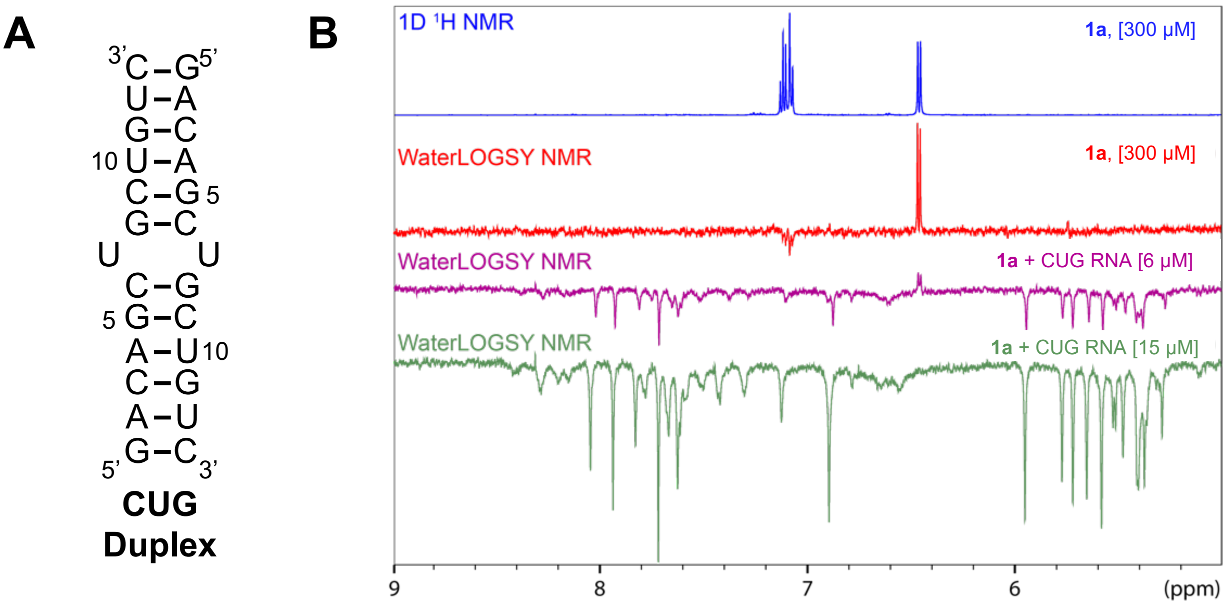

Figure S3. NMR spectral analysis of 1a interacting with r(CUG) repeats. A) Duplex model of the 1×1 U/U internal loop that forms a periodic array of r(CUG) repeats. B) Binding of **1a** to a model the r(CUG) repeat duplex as determined by an NMR WaterLOGSY experiment.

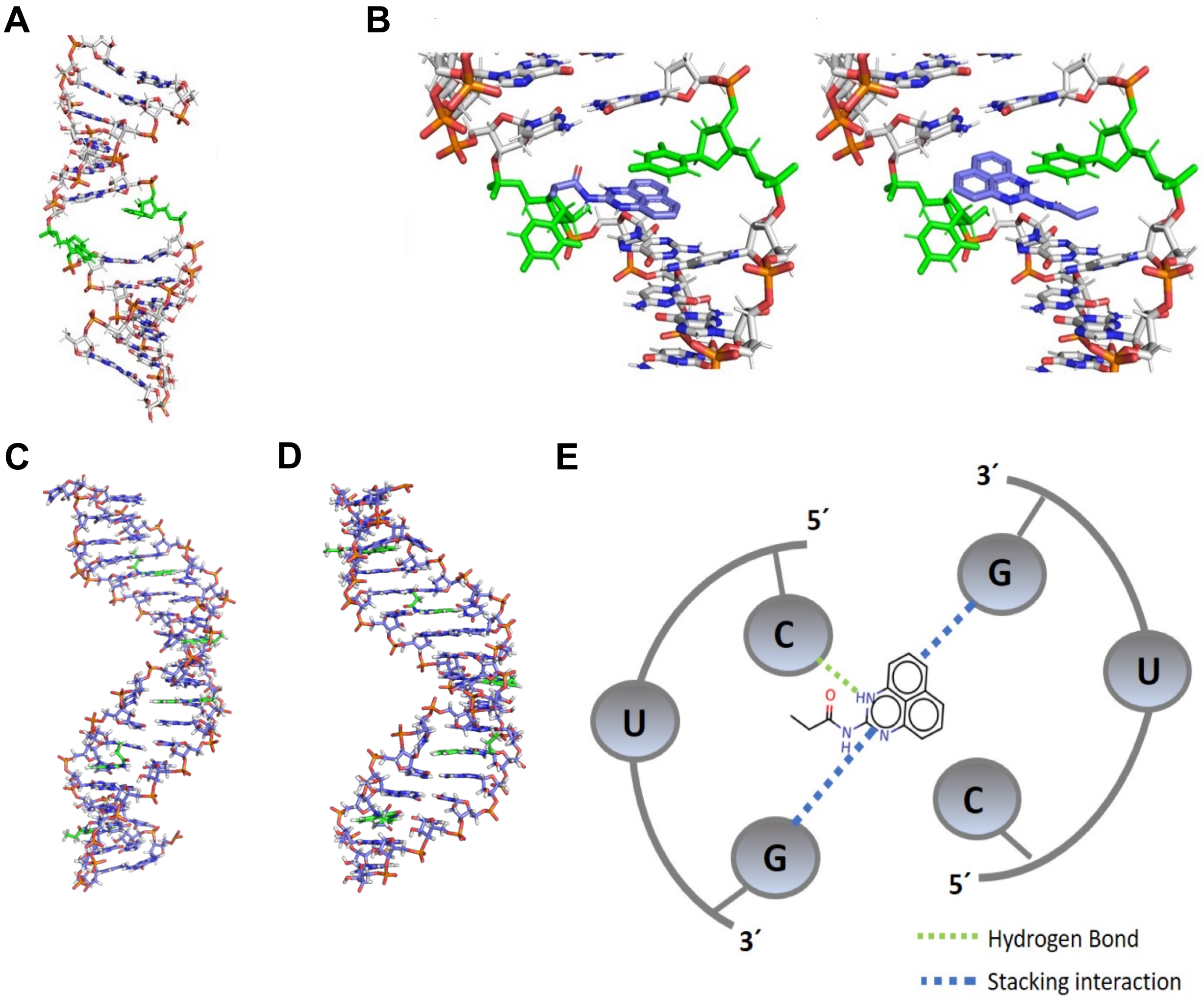

Figure S4. Poses adopted by 1a in the binding pocket of a r(CUG) repeat model. A) r(CUG) repeat model with the 1×1 U/U internal loop highlighted in green. B) Docking of **1a** with the two most populated clusters with binding energies of Left: –6.82 kcal/mol and Right: –6.58 kcal/mol. C) Initial duplex model of r(CUG) in complex with **1a**. D) Hairpin model of r(CUG) in complex with **1a**. E) Hydrogen bond and stacking interactions formed between **1a** and the neighboring base pairs of the U/U internal loop.

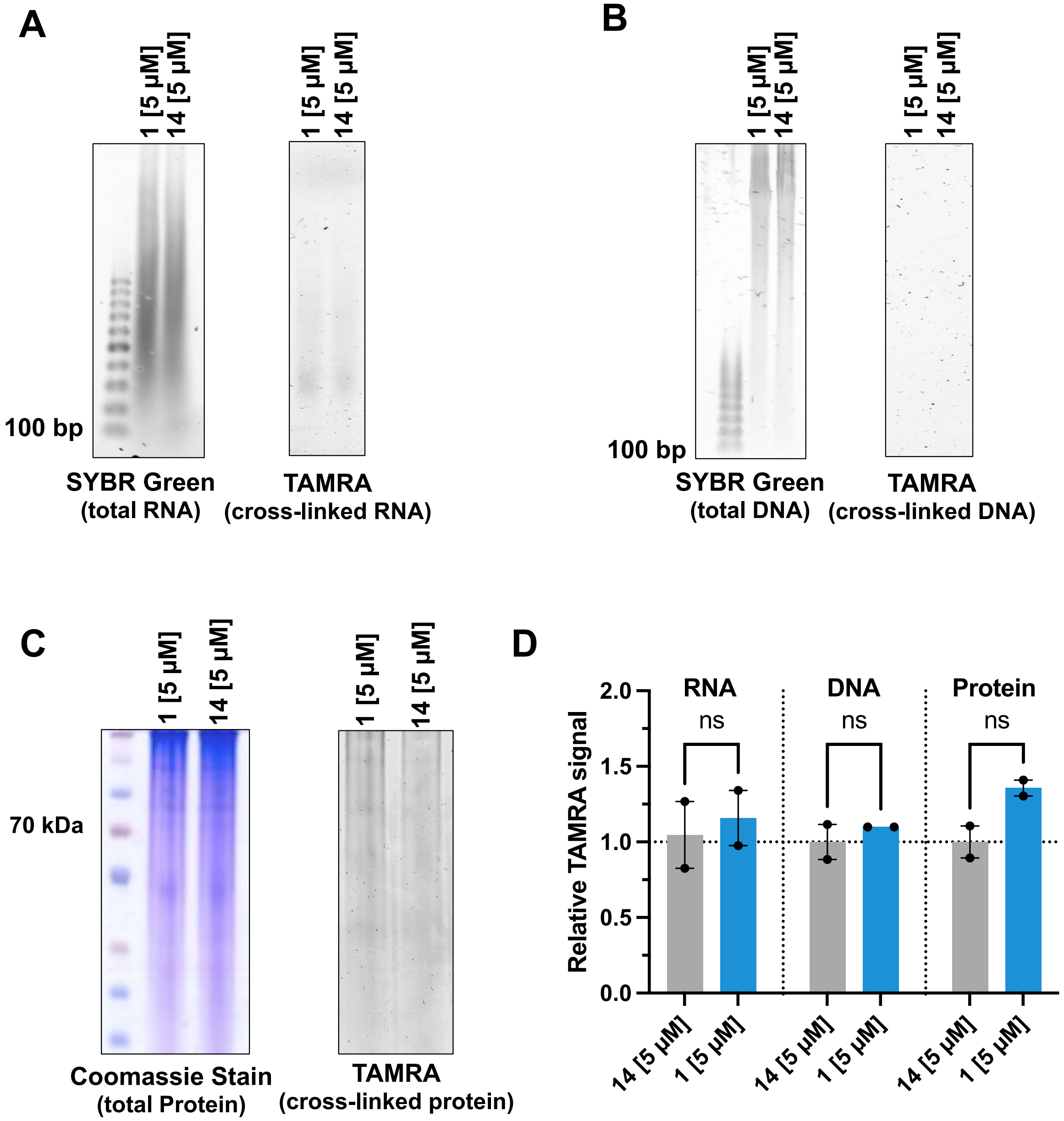

Figure S5. Selectivity assessment towards RNA, DNA, and proteins by compound 1 in DM1 myotubes. DM1 cells were treated with 5 µM of compound **1** or **14** overnight. Cells were UV irradiated and total RNA, DNA and proteins were harvested, followed by click with TAMRA azide and analysis by gel electrophoresis. A) Agarose gel (1%, w/v) displaying crosslinked RNA (TAMRA channel) or total RNA (SYBR Green channel). B) Agarose gel (1.5%, w/v) displaying crosslinked DNA (TAMRA channel) or total DNA (SYBR Green channel). C) SDS-polyacrylamide gel (10%) displaying crosslinked protein (TAMRA channel) or total protein (Coomassie stain). D) Quantification of panel A, B (n = 2) normalized to the compound **14** lane. Statistics determined by a Two-way ANOVA with multiple comparisons. All data are reported as the mean ± SEM.

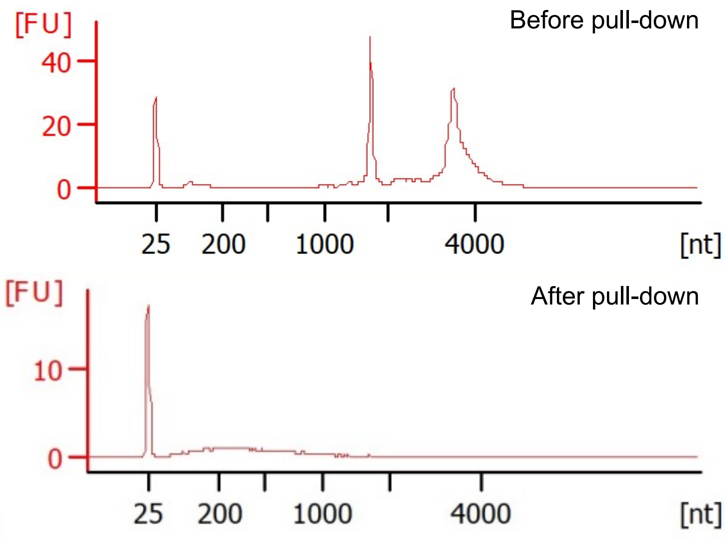

Figure S6. Bioanalyzer profile showing partial fragmentation of the RNA after pull-down. The conditions used during the pull-down induces partial fragmentation of the RNA samples with a maximal RNA-fragment length of ~1000 nucleotides.

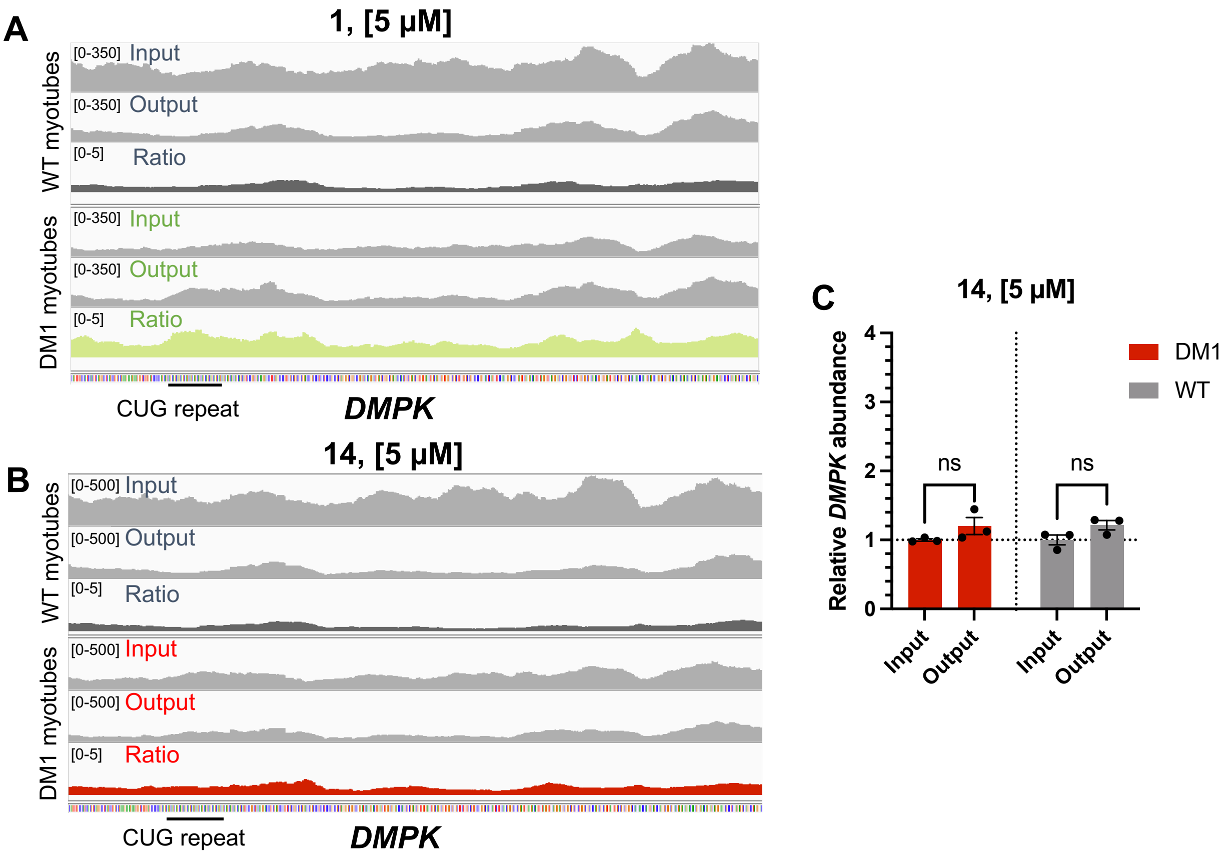

Figure S7. Chem-CLIP-Seq analysis of 1 and control Chem-CLIP probe 14 in differentiated myotubes. A) RNA-seq tracks showing *DMPK* (Chr19:45,770,149-45,770,648) including 21 r(CUG) repeat of the Hg38 reference genome in DM1 and WT myotubes treated with 5 µM of **1**. Input: raw sequencing track before pull-down. In the upper left corner is indicated the scale of the y-axis (reported as Read Count); Output: raw sequencing track after pull-down. In the upper left corner is indicated the scale of the y-axis (reported as Read Count); Ratio: ratio of sequencing reads after vs. before the pull-down. In the upper left corner is indicated the scale of the y-axis (reported as Fold Enrichment). B) RNA-seq tracks showing *DMPK* (Chr19:45,770,149-45,770,648) including 21 r(CUG) repeat of the Hg38 reference genome in DM1 and WT myotubes treated with 5 µM of **14**. Input: raw sequencing track before pull-down. In the upper left corner is indicated the scale of the y-axis (reported as Read Count); Output: raw sequencing track after pull-down. In the upper left corner is indicated the scale of the y-axis (reported as Read Count); Ratio: ratio of sequencing reads after vs. before the pull-down. In the upper left corner is indicated the scale of the y-axis (reported as Fold Enrichment). C) Results of Chem-CLIP-Seq, showing no enrichment of *DMPK* near the r(CUG) repeat region by control Chem-CLIP probe **14** (5 µM) in DM1 or WT myotubes (n = 3). Data are reported as the mean ± SD.

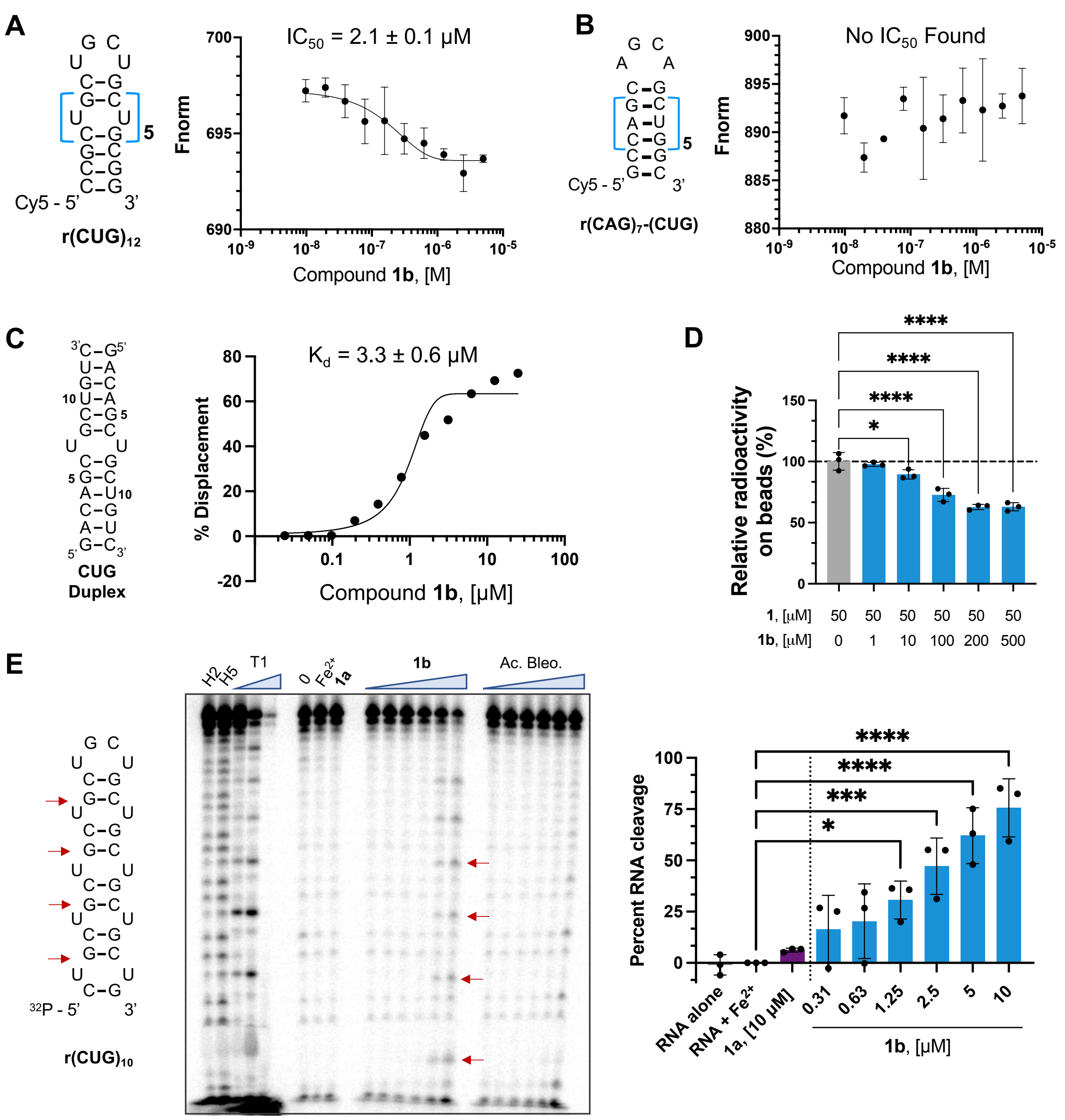

Figure S8. Compound 1b target engagement and cleavage *in vitro*. A) Representative binding curve for **1b** and r(CUG)$_{12}$, as determined by MST to (n = 2). Binding measurements were completed in a buffer lacking Fe^{2+}, required for cleavage. B) Representative binding curve for **1b** and r(CAG)$_7$-(CUG)$_5$, a fully base paired control RNA (n = 2). C) Structure of r(CUG) duplex used for affinity measurements by To-PRO-1 dye displacement and representative a binding curve for **1b** used to K_d. D) Results of an *in vitro* Competitive Chem-CLIP experiment, completed in the absence of Fe^{2+}, between **1** and **1b** (n = 3). E) Left: Representative gel image of the cleavage of ^{32}P-r(CUG)$_{10}$ by **1b**, (0.31-10 μM), acylated bleomycin (0.31-10 μM), or **1a**, (10 μM). H2 and H5 represent the hydrolysis ladders quenched respectively after 2 and 5 min of reaction and showing

cleavage at every base. T1 represents the RNase T1 ladder showing cleavage at every G base. Right: Quantification of gel autoradiograms reported as percent of r(CUG)$_{10}$ cleaved for each treatment group relative to vehicle Fe^{2+}-treated samples (n = 3); *, $p < 0.05$; ***, $p < 0.001$; ****, $p < 0.0001$; as determined by a One-way ANOVA with multiple comparisons. Data are reported as the mean ± SD.

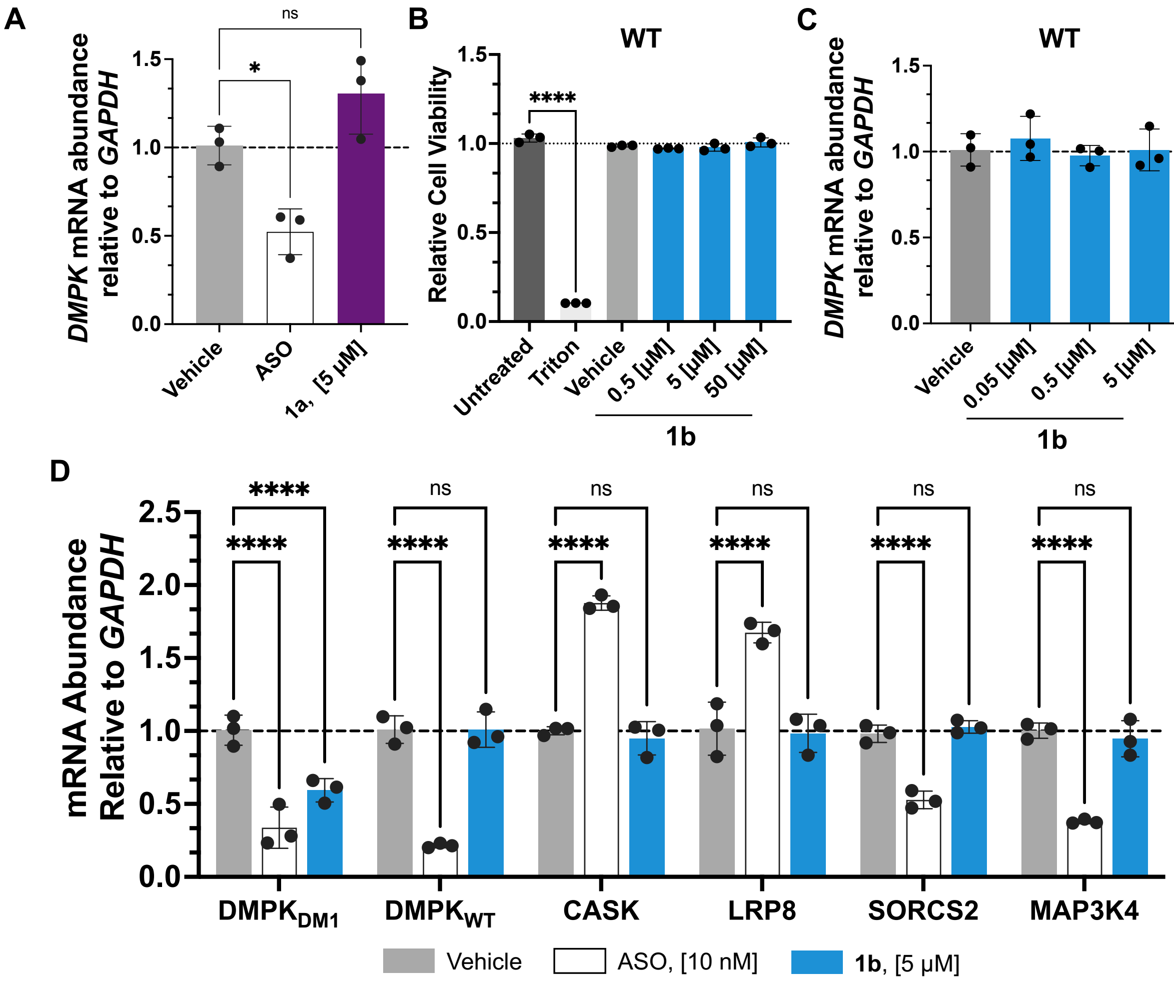

Figure S9. Effect 1a on *DMPK* levels in DM1 myotubes, effect of 1b on *DMPK* levels in WT myotubes and analysis of potential off-targets of 1b in DM1 myotubes. A) Effect of **1a** on *DMPK* abundance, which harbors r(CUG)^{exp}, in DM1 myotubes as determined by RT-qPCR (n = 3). B) Relative cell viability of **1b** in WT myotubes (n = 3). C) Effect of **1b** on *DMPK* abundance in WT myotubes as determined by RT-qPCR (n = 3). D) Effect of **1b** on transcripts containing short, non-pathogenic r(CUG) repeats in DM1 myotubes, as determined by RT-qPCR (n = 3). *, $p < 0.05$; ****, $p < 0.0001$; as determined by a One-way ANOVA with multiple comparisons. Data are reported as the mean ± SD.

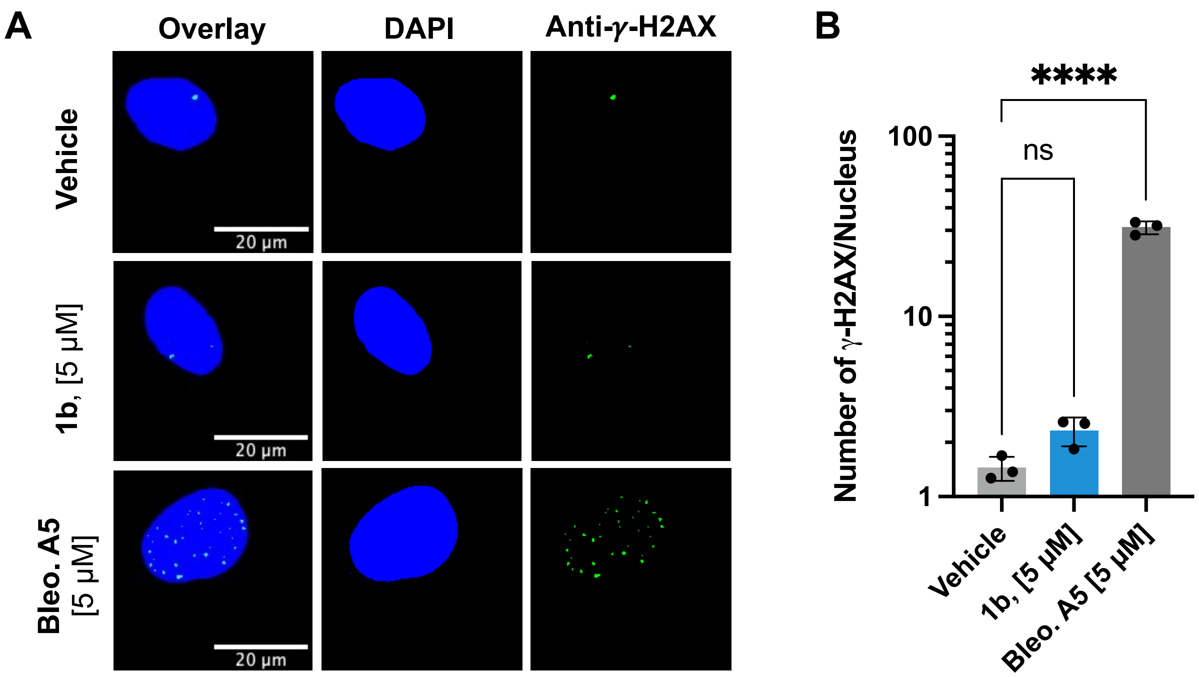

Figure S10. γ-H2AX immunostaining for 1b in DM1 myotubes. A) Images of γ-H2AX immunostaining in **1b**-treated, Bleomycin A5-treated, or vehicle- (0.1% (v/v) DMSO) treated cells. B) Quantification of the number of γ-H2AX foci per nuclei in DM1 myotubes cells treated with vehicle, 5 µM of **1b**, or 5 µM of Bleomycin A5 (n = 3, with 40 nuclei quantified/replicate). ****, $p < 0.0001$; as determined by a One-way ANOVA with multiple comparisons. Data are reported as the mean ± SD.

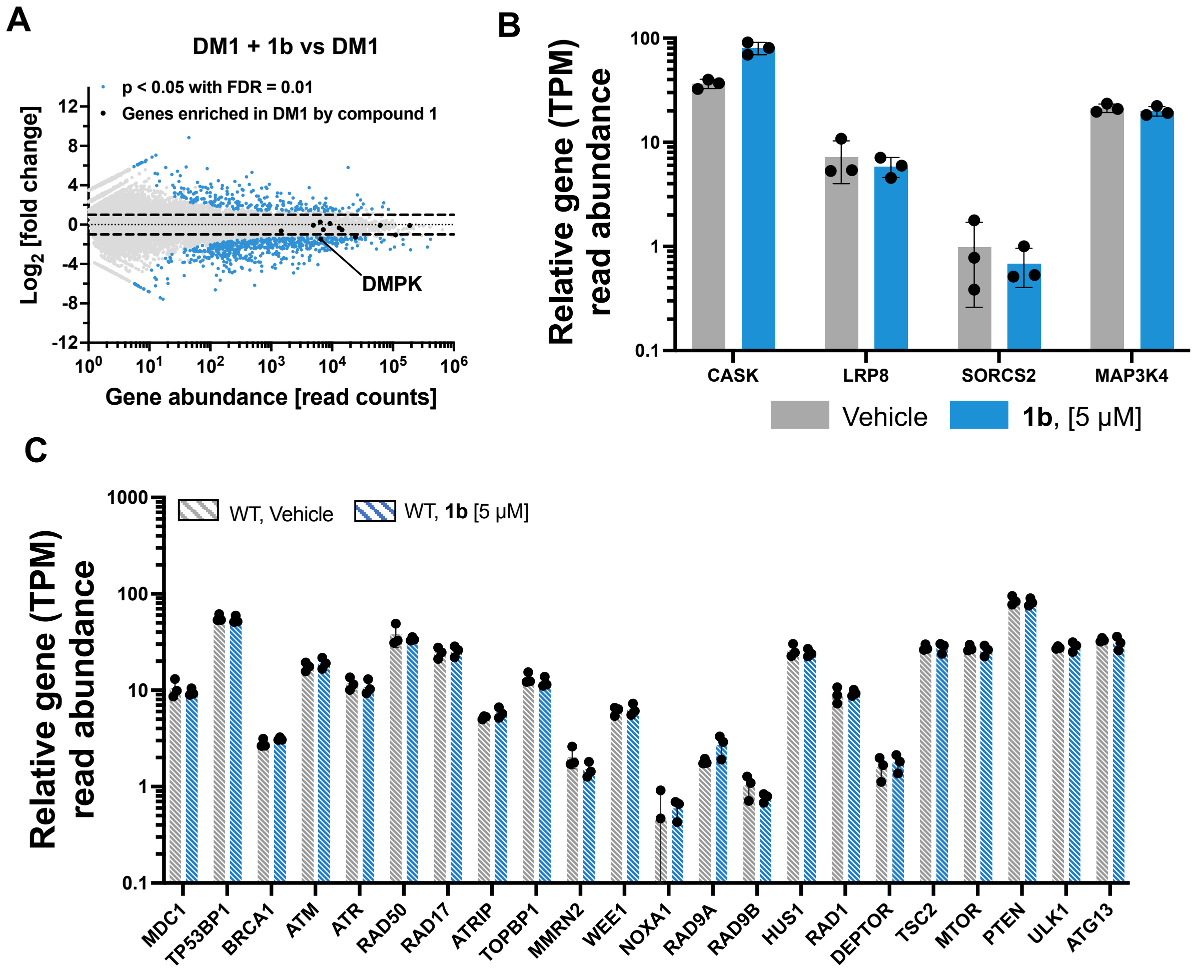

Figure S11. RNA sequencing analysis of potential off-targets and DNA damage pathway in DM1 and healthy myotubes treated with 1b. A) Gene expression RNA-Seq analysis of DM1 myotubes when treated with 5 µM of **1b** compared to treatment with 0.1% (v/v) DMSO (vehicle). Data are plotted as average Log_2 (Fold Change) vs gene abundance (n = 3). B) RNA-seq analysis of transcripts containing short, non-pathogenic r(CUG) repeats in DM1 myotubes treated with either **1b** [5 µM] or 0.1% (v/v) DMSO (vehicle), (n = 3). C) RNA-seq analysis of genes involved in DNA damage response pathways in WT cells treated with either **1b** [5 µM] or 0.1% (v/v) DMSO (vehicle), (n = 3). Data are reported as the mean ± SD.

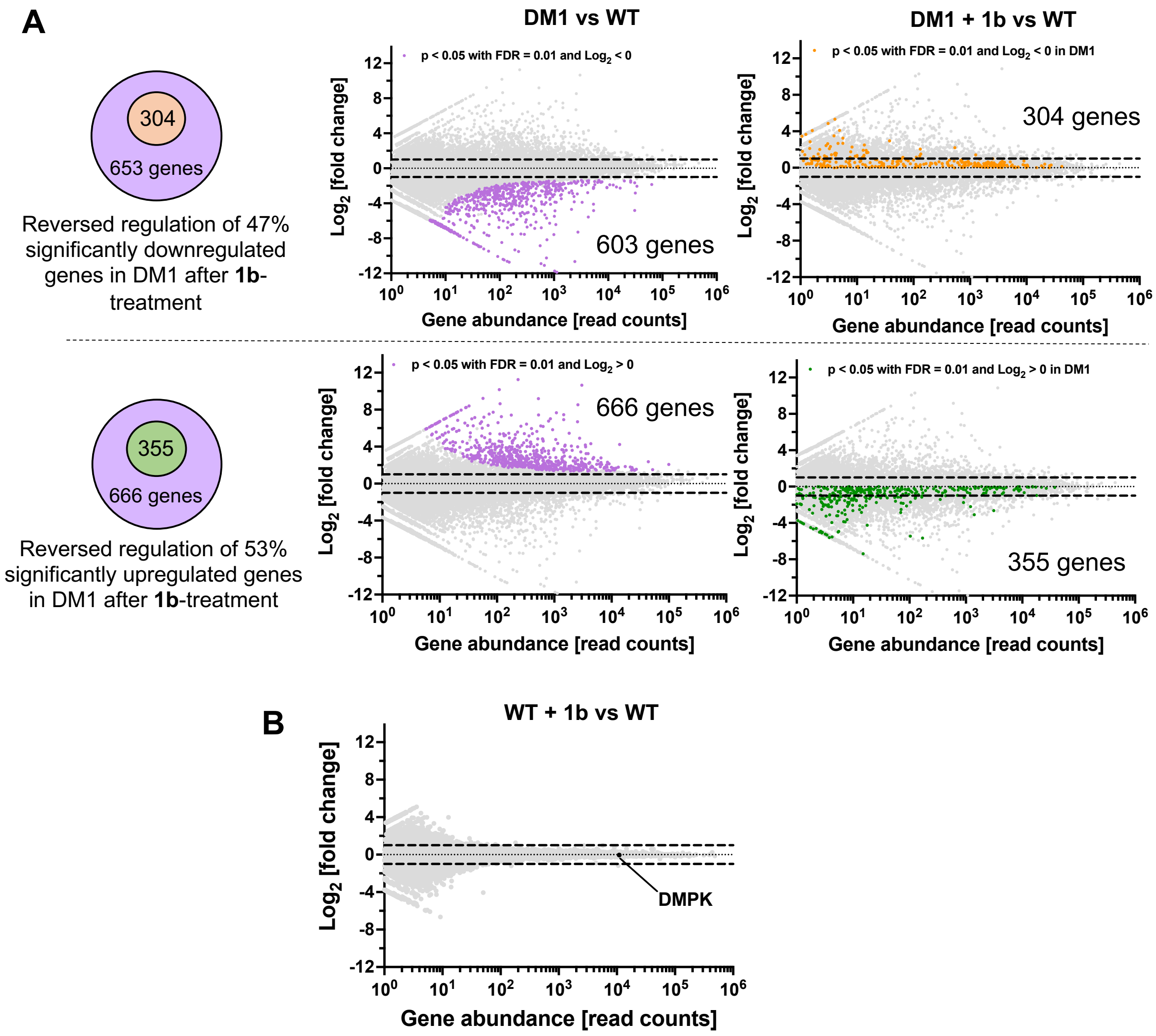

Figure S12. RNA sequencing analysis of DM1 and healthy myotubes treated with 1b. A) Top: Schematic and analysis of the number of genes significantly ($p < 0.05$) downregulated in DM1 myotubes (653 genes; as compared to WT myotubes) whose levels are upregulated (Log2(fold change) > 0), (304 genes) after treatment with 5 µM of **1b** and Bottom: Schematic and analysis of the number of gene significantly upregulated ($p < 0.05$) in DM1 (666 genes; as compared to WT myotubes) whose levels are downregulated (Log2(fold change) < 0), (355 genes) after **1b**-treatment. B) Gene expression RNA-Seq analysis of WT myotubes when treated with 5 µM of **1b** compared to treatment with 0.1% (v/v) DMSO (vehicle). Data are plotted as average Log_2 (Fold Change) vs gene abundance ($n = 3$).

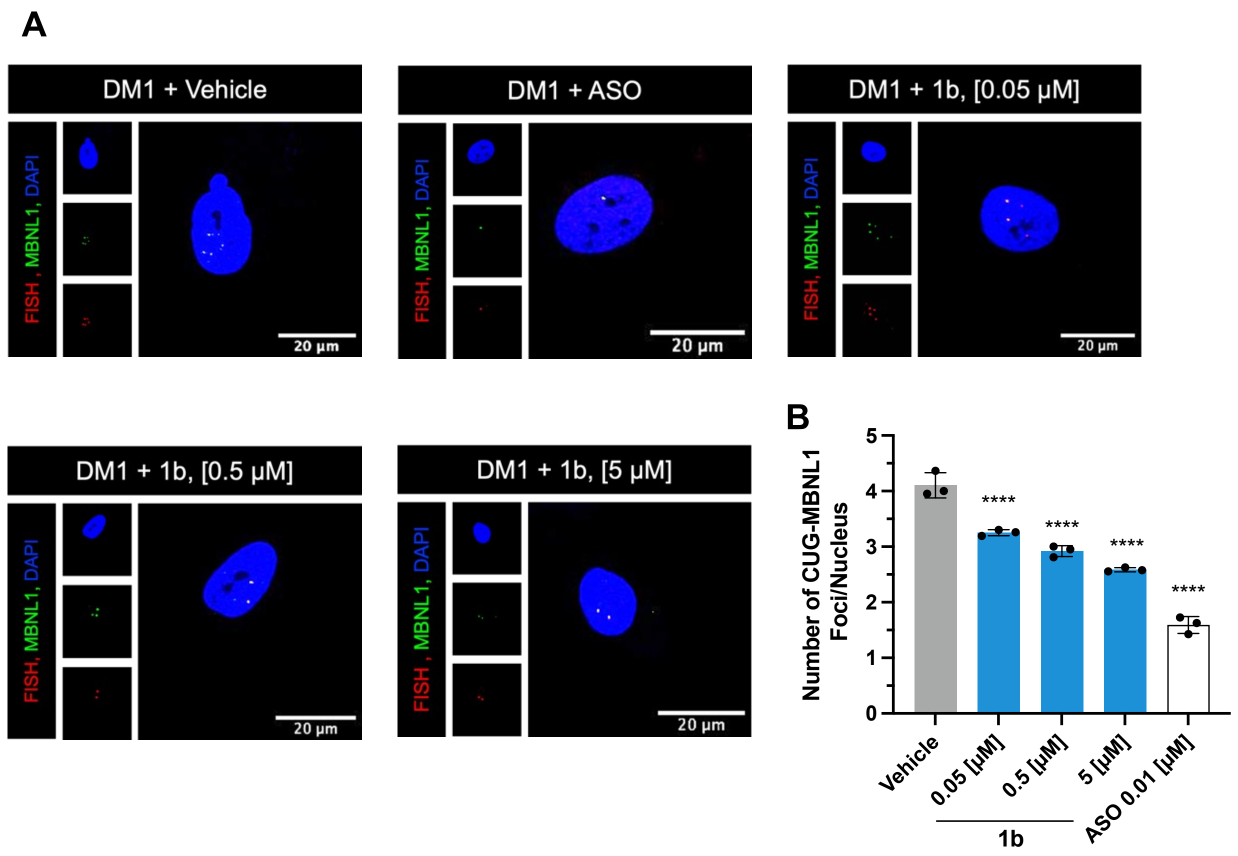

Figure S13. Effect of 1b on the number of r(CUG)^{exp}–MBNL1 foci in DM1 myotubes. A) Representative images of r(CUG)^{exp}–MBNL1 foci imaged by RNA fluorescence in situ hybridization (FISH) and anti-MBNL1 immunostaining treated with 10 nM ASO or 0.05, 0.5 and 5 μM of **1b** compared to vehicle (0.1% (v/v) DMSO). B) Quantification of r(CUG)^{exp} foci in the nuclei of treated and untreated DM1 myotubes ($n = 3$, with 40 nuclei quantified/replicate). ****, $p < 0.0001$ as determined by a One-way ANOVA with multiple comparisons. Data are reported as the mean ± SD.

A

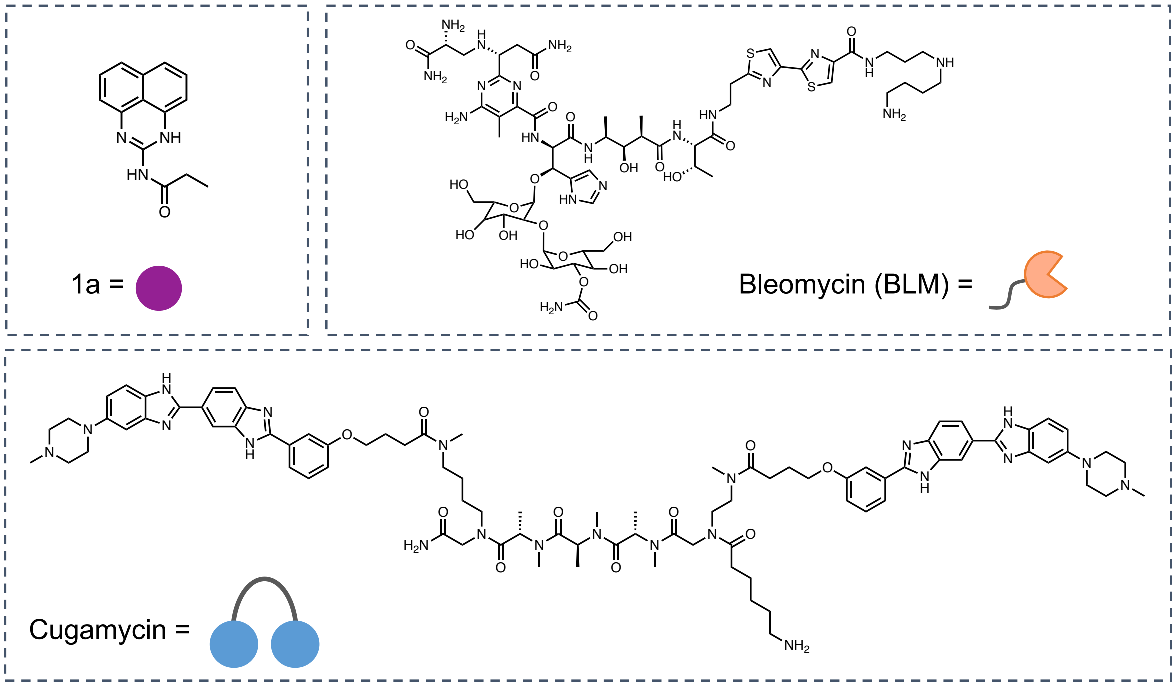

B

Compound	MW	Splicing	Cleavage	QED
**1a**	239.28	NA	NA	0.799
**1b**	1933.11	5 μM	5 μM	NA
Cugamycin	1627.03	10 μM	NA	0.018
BLM, Cugamycin	3093.59	4 μM	4 μM	NA

Figure S14. Comparison of activity and physiochemical properties of compound 1a and previously reported Cugamycin. A) Chemical structures of **1a**, bleomycin A5 (BLM), and Cugamycin.[2] B) Evaluation compounds molecular weight (g/mol), concentration at which ~50% rescue is observed MBNL1 splicing and *DMPK* cleavage in DM1 myotubes, and computationally determined quantitative estimation of drug likeness (QED).

**Table S1: Transcripts enriched by 1 in Chem-CLIP-Seq of DM1 myotubes**			
**#**	**Genes**	**Full name**	**Log_2(Fold of enrichment)**
1	*COL6A1*	Collagen Type VI Alpha 1 Chain	0.86
2	*DBN1*	Drebrin 1	0.89
**3**	***DMPK***	**Dystrophia Myotonica Protein Kinase**	**0.91**
4	*JUN*	Jun Proto-Oncogene, AP-1 Transcription Factor Subunit	0.98
5	*PLEC*	Plectin	1.02
6	*ARHGDIA*	Rho GDP Dissociation Inhibitor Alpha	1.05
7	*TLE5*	TLE Family Member 5, Transcriptional Modulator	1.10
8	*PPDPF*	Pancreatic Progenitor Cell Differentiation and Proliferation Factor	1.16
9	*WDR26*	WD Repeat Domain 26	1.80
10	*H1-2*	H1.2 Linker Histone, Cluster Member	1.31
11	*RTN4*	Reticulon 4	1.43
12	*BCAR1*	BCAR1 Scaffold Protein, Cas Family Member	1.92

Table S2: Transcripts enriched by 1 in Chem-CLIP-Seq of WT myotubes			
**#**	**Genes**	**Full name**	**Log2**
1	*CASTOR2*	Cytosolic Arginine Sensor for MTORC1 Subunit 2	0.84
2	*SLC7A5*	Solute Carrier Family 7 Member 5	0.89
3	*SNHG8*	Small Nucleolar RNA Host Gene 8	1.05
4	*CCN1*	Cellular Communication Network Factor 1	1.17
5	*SNORD3D*	Small Nucleolar RNA, C/D Box 3D	1.27

Table S3: Primers used for PCR amplification, both qPCR and end-point PCR		
**Primers**	**Forward Sequence (5'→3')**	**Reverse Sequence (5'→3')**
CASK (RT-qPCR)	TTGAAATCGTAAAGCGAGCTGA	CAGTAGCGTAGAGCTTCCAGTA
DMPK (RT-qPCR)	CGTGCAAGCGCCCAG	CTCCACCAACTTACTGTTTCATCCT
GAPDH (RT-qPCR)	AAGGTGAAGGTCGGAGTCAA	AATGAAGGGGTCATTGATGG
HeLa_CUG (RT-qPCR)	CGATCTCTGCCTGCTTACTC	GTCGGAGGACGAGGTCAATAAA
HeLa_GAPDH (RT-qPCR)	GAAGGTGAAGGTCGGAGT	GAAGATGGTGATGGGATTTC
LRP8 (RT-qPCR)	GCCAAGGATTGCGAAAAGGAC	GTGGTCTAAGCAGTCATCGTC
MAP3K4 (RT-qPCR)	CAATAAGCCTTACCTCAGCCTTG	GTTAAGCCAGAAACCAGACGTA
MAP4K4_ex22a (RT-PCR)	CCTCATCCAGTGAGGAGTCG	ATCACAGGAAAATCCCACCA
MBNL1_ex5 (RT-qPCR)	CTCAGTCGGCTGTCAAATCA	AGAGCAGGCCTCTTTGGTAA
MBNL1_ex5 (RT-PCR)	GCTGCCCAATACCAGGTCAAC	TGGTGGGAGAAATGCTGTATGC
SCUBE2 (RT-qPCR)	CCCACCTCCTACAAGTGCTC	TGCAACGATAATTGCCTGGAAT
SORCS2 (RT-qPCR)	CACGTCGTTCGTGCTCAAG	CGTCCCGAAATCTGATGACCG

Probes for TaqMan qPCR in HeLa cellular model of DM1 [3]	
**Probe**	**Sequence (5'→3')**
Probe 1 (recognizes WT allele)	/56FAM/AGAGCAGCG/ZEN/CAAGTGAGGAGG/3IABkFQ/
Probe 2 (recognizes mutant allele)	/5HEX/TGACGCAGC/ZEN/CACGTGAAGGTC/3IABkFQ/

**Table S4: Atom names, type and charges used for parametrization of compound 1a**		
**ATOM NAME**	**ATOM TYPE**	**ATOM CHARGES**
C	c3	-0.089100
C1	c3	-0.149400
C2	c	0.674100
O	o	-0.593100
N	ns	-0.571400
C3	cc	0.689400
N1	nd	-0.782000
C4	ca	0.444600
C5	ca	-0.159300
C6	ca	0.074700
N2	na	-0.403900
C7	ca	-0.218000
C8	ca	-0.078000
C9	ca	-0.168000
C10	ca	0.018000
C11	ca	-0.162000
C12	ca	-0.091000
C13	ca	-0.220300
H	hc	0.051033
H1	hc	0.051033
H2	hc	0.051033
H3	hc	0.070700
H4	hc	0.070700
H5	hn	0.347500
H6	hn	0.328700
H7	ha	0.138000
H8	ha	0.131000
H9	ha	0.135000
H10	ha	0.134000
H11	ha	0.130000
H12	ha	0.145000

Table S5: RISM calculated binding free energies for seven most populated clusters

Cluster #	ΔG^{o}_{37} (kcal/mol)
1	-10.36
2	-12.42
3	-10.41
4	-8.75
5	-12.06
6	-10.63
7	-12.49

Supplementary Methods:

General Methods. RNAs and Cy5-labeled RNAs, the latter purified by HPLC by the vendor, were purchased from Dharmacon (GE Healthcare). For unlabeled RNAs, deprotection of the 2'-ACE protecting group and subsequent desalting using PD-10 columns (GE Healthcare) were performed according to the vendor's recommended procedure. RNA concentration was determined by its absorbance at 260 nm, measured with a Beckman Coulter DU 800 UV/vis spectrophotometer, and the extinction coefficient provided by the manufacturer. Antisense oligonucleotide was purchased from Qiagen LLC. The sequence of the CAG gapmer, complementary to the r(CUG) repeats, used in this study is +A+G+CA*G*C*A*G*C*A*G*C*A*+G+C+A where locked-nucleic acid (LNA) modifications are indicated by a "+" and phosphorothioate modifications are indicated by a "*".

5'-^{32}P Labeling of r(CUG)$_{10}$. An equivalent of 1 nmole of RNA was radiolabeled with [γ-^{32}P]ATP (PerkinElmer) using T4 polynucleotide kinase (New England Biolabs) at 37 ˚C for 45 min and purified by using a denaturing 15 % (v/v) polyacrylamide gel. The RNA was imaged by UV shadowing, excised from the gel, and tumbled in 300 mM NaCl for 3 h at 4 ˚C. Glycogen (20 µg, (RNA grade; Invitrogen) was added to the solution, and the RNA was precipitated with ethanol (1 mL) for 15 min at -80 ˚C and centrifuged to pellet the RNA, which was dissolved in 40 µL of Nanopure water.

***In vitro* Chem-CLIP.** *In vitro* Chem-CLIP was performed as previously described.[4] Briefly, radiolabeled r(CUG)$_{10}$ (~2000 CPM/sample) was folded in 20 µL of 20 mM Hepes, pH 7.5, by heating at 95 ˚C for 30 s followed by snap-cooling on ice for 5

min. Compound at the appropriate concentration was then added to the RNA samples and incubated for 15 min at room temperature, followed by irradiation with UV light (365 nm) for 15 min using a UVP Crosslinker (UV Stratalinker 2400). After UV crosslinking, a freshly prepared "click mixture" composed of $CuSO_4$ (1 µL, 10 mM), THPTA (0.6 µL, 50 mM, Sigma-Aldrich, #760952-88-3), PEG_3 biotin azide (1.0 µL, 10 mM, Click Chemistry Tools, #AZ104) and sodium ascorbate (0.6 µL, 250 mM, pH 7.0) was added to each well, and the samples were incubated at 37 ˚C for 3 h. Next, 15 µL of streptavidin magnetic beads (slurry; Dynabeads MyOne Streptavidin C1 beads; Thermo Scientific, #65001) were added to each well, and the samples were incubated for an additional 15 min at room temperature. Unreacted (not cross-linked) RNA in the supernatant was removed using a magnetic separation rack. The beads were washed three times with 1× Wash Buffer (10 mM Tris-HCl pH 7.0, 1 mM EDTA, 4 M NaCl, and 0.2% (v/v) Tween-20. Radioactive signal associated with the beads and the supernatant from the washes was measured by liquid scintillation counting.

***In vitro* RNA Cleavage by 1b.** *In vitro* cleavage was completed as previously described.[5] Briefly, 3 µL of *in vitro* transcribed 5'-^{32}P labeled r(CUG)$_{10}$ (600K CPM) was diluted with 200 µL of 5 mM NaH_2PO_4, pH 7.4 and heated to 95 °C for 30 s followed by snap-cooling on ice for 5 min. Compound **1b** was added at varying concentrations (10, 5, 2.5, 1.25, 0.6 and 0.3 µM), followed by addition of an equimolar amount of freshly prepared $(NH_4)_2Fe(SO_4)_2·6H_2O$ in 5 mM NaH_2PO_4, pH 7.4. The solutions were incubated for 30 min at 37 °C and then supplemented with additional equimolar aliquots of $(NH_4)_2Fe(SO_4)_2·6H_2O$ and supplemented again after 30 more min (60 min post first addition). The samples were incubated for a total of 24 h at 37 °C. Reactions were stopped

by adding an equal volume of 2× Loading Buffer (8 M urea, 20 mM EDTA, pH 7.5, 0.05% (w/v) bromophenol blue and 0.05% (w/v) xylene cyanol).

T1 and hydrolysis ladders were prepared as follows: RNase T1 (3 units/µL final concentration, ThermoFisher Scientific) was added to 1 µL of radiolabeled RNA in 10 µL of 1× T1 buffer (20 mM sodium citrate, pH 5.0, 1 mM EDTA, and 7 M urea), and the sample was incubated at room temperature for 20 min. The reaction was then stopped by adding an equal volume of 2× Loading Buffer. A hydrolysis ladder was prepared by mixing 1 µL of radiolabeled RNA with 10 µL of 1× Alkaline Hydrolysis Buffer (50 mM $NaHCO_3$, pH 9.2, and 1 mM EDTA) and heating at 95 °C for 5 min. The reaction was stopped by adding an equal volume of 2× Loading Buffer.

All samples were analyzed using a denaturing 15 % (v/v) polyacrylamide gel run at 70 W for 3 h in 1× TBE buffer. Gels were exposed to a phosphorimaging screen overnight at -20 ˚C and then imaged using a Typhoon 9410 variable mode imager. The amount of cleaved RNA was quantified using ImageLab (BioRad) and normalized to the percent cleaved when nucleic acid was treated with Fe^{2+} only (3 replicates for all samples).

Binding Affinity Measurements by Microscale Thermophoresis (MST). [6] MST measurements were performed on a Monolith NT.115 system (NanoTemper Technologies) with Cy5-labeled $r(CUG)_{12}$ (5'-Cy5-GCG$(CUG)_{12}$CGC; Dharmacon) or Cy5-labeled base pair control (BP) (5'-Cy5-GCG$(CUG)_5(CAG)_7$CGC; Dharmacon). RNA (10 nM) was prepared in 1× MST Buffer (8 mM Na_2HPO_4, pH 7.0, 185 mM NaCl, and 1 mM EDTA) and folded by heating at 95 ˚C for 60 s and cooling down on ice for 5 min. Then, 10 µL of nucleic acid was added to an equal volume of compound of interest at 2×

concentration prepared in 1× MST Buffer supplemented with 0.1% (v/v) Tween-20. Samples were incubated for 15 min at room temperature in the dark and then loaded into standard capillaries (NanoTemper Technologies). The following parameters were used to measure thermophoresis: 10% LED, 80% MST power, Laser-On time = 30 s and Laser-Off time = 5 s. Fluorescence was measured using excitation wavelengths of 605–645 nm and emission wavelengths of 680–685 nm. For each curve two independent experiments were performed, each with two technical replicate scans. The ΔF_{norm} for each concentration in the two technical replicates (scans) were averaged and then plotted as a function of compound concentration. The resulting curve was fit to Equation 1 (Prism GraphPad) to afford the IC_{50}.

$$IC_{50} = d + \frac{a-d}{[1+\left(\frac{x}{c}\right)^b]} \qquad \text{(Eq. 1)}$$

where a is the theoretical response at zero concentration; b is the slope factor; c is the inflection point; d is the theoretical response at infinite concentration, and x is the concentration of small molecule. The reported IC_{50} is the average from curve fitting, and the error is the standard deviation of the IC_{50}s.

Stoichiometry measurement by MST. As previously described, MST measurements were performed on a Monolith NT.115 system (NanoTemperTechnologies) with Cy5-labeled $r(CUG)_{12}$ and unlabeled $r(CUG)_{12}$. An RNA concentration of 25 times the measured IC_{50} of **1a** (220 nM) was selected for this MST experiment, as recommended by the manufacturer's protocol. A 2× mixture of Cy5-labeled and unlabeled RNA (0.05 µM Cy5-labeled and 5.45 µM unlabeled RNA) was prepared in 1× MST buffer and folded by heating at 95 °C for 60 s and snap-cooling on ice for 5 min. Compound **1a**, prepared

in 1× MST Buffer containing 0.1% Tween-20 (v/v) at varying concentrations, was added to wells of non-binding black 384-well plates (Greiner, #784900). The folded RNA and compound were then mixed 1:1 (v/v). Samples were incubated for 20 min at room temperature in the dark and then loaded into premium capillaries (NanoTemper Technologies). The following parameters were used: 1 % LED, 80 % MST power, Laser-On time = 30 s, Laser-Off time = 5 s. Fluorescence was detected using excitation wavelengths of 605–645 nm and emission wavelengths of 680–685 nm. The resulting data were analyzed to afford ΔF_{norm}, which was plotted as a function of compound concentration. The concentration where saturation occurred was determined by the intersection of two linear regressions, and the stoichiometry was calculated by dividing the saturation concentration by the concentration of RNA used in the experiment.

Affinity Measurements by To-Pro-1 Dye Displacement. To measure the affinity of To-Pro-1 for the 5'-(GACAG<u>CUG</u>CUGUC)$_2$-3' duplex harboring a single 5'C<u>U</u>G/3'G<u>U</u>C, the RNA (500 nM) was folded by heating at 95°C for 2 min 1 × Assay Buffer (8 mM NaH_2PO_4, pH 7.0, 200 mM NaCl, and 1 mM EDTA), followed by slowly cooling to room temperature on the bench top. Once cooled, TO-Pro-1 and BSA were added to final concentrations of 100 nM and 40 μg/mL, respectively. Serial dilutions of 1:1 were made using 1× Assay Buffer supplemented with 100 nM TO-Pro-1 and 40 μg/mL BSA with the final sample containing no RNA. The samples were then incubated at room temperature for 15 min. Two independent experiments with three technical replicates each were measured for each sample in a 384-well plate. Fluorescence was measured by using a Tecan plate reader with the following parameters: Excitation/Emission

wavelengths: 485/520nm; Bandwidth for Excitation/Emission: 5/10 nm; Gain: 100. The resulting curve of change in fluorescence as a function of RNA concentration was fit to Equation 2 (Specific binding with Hill slope in GraphPad Prism), a one site binding model, to afford the K_d.

$$y = \frac{B_{max} * X^h}{({K_d}^h + X^h)} \qquad \text{(Eq. 2)}$$

where B_{max} is the maximum specific binding; K_d is the concentration required to achieve a half-maximum binding at equilibrium; and h is the Hill slope. Error is reported as standard deviation calculated from the resultant K_ds.

To measure the affinity of **1**, **1a**, and **1b** for 5'-(GACAG<u>CUG</u>CUGUC)$_2$-3' duplex harboring a single 5'C<u>U</u>G/3'G<u>U</u>C, the RNA (400 nM) was folded by heating at 95 °C for 2 min 1 × Assay Buffer, followed by slowly cooling to room temperature on the bench top. Once cooled, TO-Pro-1 and BSA were added to final concentrations of 100 nM and 40 µg/mL, respectively. The samples were then incubated at room temperature for 5 min. The compound of interest was then added to the samples at the indicated concentrations where the final concentration of DMSO is <1% (v/v). After incubating for an additional 15 min at room temperature, the samples were aliquoted into a 384-well plate (Greiner #784076) in three technical replicates of 10 µL each. Controls wells included 1 × Assay Buffer supplemented with To-Pro-1 and BSA alone (minimum signal) and RNA in 1 × Assay Buffer supplemented with To-Pro-1, BSA alone, and vehicle (maximum signal). Two independent experiments were performed, with three technical triplicates measured per experiment. Fluorescence was measured by using a Tecan plate reader with the following parameters: Excitation/Emission wavelengths: 485/520nm; Bandwidth for

Excitation/Emission: 5/10 nm; and Gain: 100. The change in fluorescence as a function of compound concentration was fit to a competitive curve fit (Equations 3 & 4), to afford the K_d.

$$y = Bottom + \left(\frac{Top - Bottom}{1 + 10^{(X - LogEC_{50})}}\right) \qquad \text{(Eq. 3)}$$

$$LogEC_{50} = log\left(10^{logK_i}\left(\frac{1 + [\text{To-Pro-1}]}{K_{d,\text{ To-Pro-1}}}\right)\right) \qquad \text{(Eq. 4)}$$

where EC_{50} is the concentration of compound (**1**, **1a**, or **1b**) that displaces half of To-Pro-1 as determined by the baseline (Bottom) and maximum response (Top); K_i is the molar equilibrium dissociation constant of **1**, **1a**, or **1b**; [To-Pro-1] is the concentration of To-Pro-1 (100 nM); $K_{d,\text{To-Pro-1}}$ is the equilibrium dissociation constant of To-Pro-1 and the RNA duplex (31 ± 2 nM). Error is calculated as the standard deviation from the resultant K_ds of the two independent experiments.

NMR Spectroscopy.[7] NMR spectra for WaterLOGSY (water-ligand observed via gradient spectroscopy) and 1D imino and aromatic proton experiments were acquired on a Bruker Advance III 600 MHz spectrometer equipped with a cryoprobe. Duplex RNA, r(5'-GACAGCUGCUGUC-3') was purchased from Dharmacon (GE Healthcare) and deprotected per manufacturers protocol before desalting with a PD-10 desalting column (GE Healthcare). RNA stocks were diluted with NMR Buffer (10 mM Na_2HPO_4/NaH_2PO_4, pH 6.0, 0.05 mM EDTA). NMR samples were refolded at 95 °C for 5 minutes and slow cooled to room temperature to favor duplex formation.

WaterLOGSY experiments were carried out on r(CUG) repeat mimic duplex mixed with **1a** at 25°C (298°K). Samples for WaterLOGSY experiments were dissolved in 5%

D_2O (Cambridge Isotope Labs) and 95% H_2O and contained 300 μM compound. RNA was then added to final concentrations of 3 μM and 15 μM, affording final ratios of RNA/compound of 20 and 100, respectively. The spectra were phased to give negative signals for negative NOEs with water.

1D ^{1}H-NMR spectra of imino protons in the r(CUG) duplex (100 μM) were recorded at 5 ˚C (278 ˚K). After acquiring the spectra of the RNA alone, **1a** was then titrated into the sample to final concentrations of 50 μM, 100 μM, 150 μM and 200 μM, affording final compound/RNA molar ratios of 0.0, 0.5, 1.0, 1.5 and 2.0.

Spectra of the aromatic RNA protons were recorded at 25 ˚C using 100 μM RNA prepared in 99.9% D_2O (Cambridge Isotope Labs). In these experiments, **1a** was titrated into the sample to final concentrations of 50 μM, 100 μM, 150 μM, and 200 μM, affording final compound/RNA molar ratios of 0.0, 0.5, 1.0 1.5, and 2.0.

1D spectra for WaterLOGSY as well as imino and aromatic proton experiments were processed using TopSpin 4.1.1 (Bruker).

Cell Culture. FDM1 (1300 CUG repeats) conditional MyoD-fibroblast cells and wild-type conditional MyoD-fibroblast cells [8] (gifts from D. Furling; Centre de Recherche en Myologie (UPMC/Inserm/CNRS), Institut de Myologie, Paris France) were grown in growth medium composed of 1× DMEM (Corning, #15-017-CV), 10% (v/v) FBS (Gibco, #10437-028), 1% (v/v) Antibiotic-Antimycotic Solution (Corning, #30-004-CI) and 2 mM L-alanyl-L-glutamine (Glutagro; Corning, #20-015-CI). After reaching ~90% confluency, conditional MyoD-fibroblast cells were differentiated into myotubes for 48 h using a differentiation medium composed of 1× DMEM, 1% (v/v) Antibiotic-Antimycotic Solution, 0.1 mg/mL transferrin human (Sigma, #T8158), 0.01 mg/mL insulin (Sigma,

#I0516), and 2 μg/mL doxycycline (Fisher Bioreagents, #10592-13-9). For compound treatment, cells were plated into 6-well dishes containing 2 mL of differentiation medium per well, and treated with compound diluted in DMSO (0.1% final) and cultured for 48 h. The ASO was purchased from Qiagen LLC with the following sequence: +A+G+CA*G*C*A*G*C*A*G*C*A*+G+C+A where "+" indicates a locked nucleic acid (LNA) modification and "*" indicates a phosphorothioate backbone. ASOs were transfected into 6-well dishes containing 2 mL of differentiation medium per well using Lipofectamine RNAiMax (Thermo Fisher) per manufacturer's protocol.

DM1 HeLa cells expressing WT (0 r(CUG) repeats) and mutant [r(CUG)$_{480}$] alleles [3] were cultured in 1× DMEM, 10% (v/v) FBS, 1% (v/v) Antibiotic-Antimycotic Solution, and 2 mM L-alanyl-L-glutamine (Glutagro). Treatment of HeLa cells was performed in growth medium for 48 h at 37 ˚C / 5% CO_2.

Selectivity assessment toward RNA, DNA and Proteins. DM1 cells were grown differentiated and treated in 100 mm dishes for 24 h as described above. After this 24 h differentiation period, 5 μM of **1** (0.1% (v/v) DMSO) was added to cells, and the cells were incubated for an additional 24 h in differentiation medium (48 h total time in differentiation medium: 24 h compound treatment). Cells were irradiated with UV light using a UVP Crosslinker (UV Stratalinker 2400) for 10 min in ice-cold 1× DPBS (Corning). Total RNA was then harvested using a Quick-RNA Mini-Prep (Zymo Research) per the vendor's protocol with DNase and proteinase treatment. Total DNA was harvested using a Quick-DNA Mini-Prep (Zymo Research) per the vendor's protocol with RNase and

proteinase treatment. Total protein was harvested using Mammalian Protein Extraction Reagent (M-PER, Thermo Fisher Scientific, #78501) following the vendor's recommendation with RNase and DNase treatment. Protein (25 μg), DNA (5 μg) and RNA (5 μg) were then supplemented with a freshly prepared "click mixture", composed of TAMRA azide (1 μL, 10 mM, Click Chemistry Tools, #AZ109; 1 μL, 10 mM $CuSO_4$; 1 μL, 50 mM THPTA; 1 μL, 250 mM sodium ascorbate pH 7.0) and incubated for 3 h at 37 °C. RNA and DNA samples were purified by ethanol precipitation and were respectively resolved on a 1% (w/v) and 1.5% (w/v) agarose gel in 1× TBE buffer for 1h at 110V. Protein samples were resolved on a 10% SDS-polyacrylamide gel for 1h at 120V. All gels were first imaged with TAMRA channel by using a Typhoon 9500 variable mode imager. Total proteins were imaged by Coomassie Brilliant Blue staining (Bio-Rad) and, total DNA and RNA were visualized by SYBR green staining.

RT-qPCR Analysis of *DMPK* Abundance in DM1 and WT Myotubes. Cells were grown, differentiated, and treated in 6-well plates as described above. After 48 h of compound treatment, total RNA was harvested using a Zymo Research Quick-RNA Mini Prep Kit per the manufacturer's recommended protocol. Approximately 500 ng of total RNA was reverse transcribed with a qScript cDNA synthesis kit in 20 μL total reaction volume (Quanta BioSciences) per the vendor's recommended protocol. Next, 2 μL of the RT reaction was subjected to qPCR (35 μL total volume) for each pair of primers (Table S3, 570 nM) using SYBR Green Master Mix (Thermo Fisher Scientific). The qPCR reaction was then aliquoted into three technical replicates (10 μL) and analyzed by a QuantStudio 5, 384-well Block Real-Time PCR System (Applied Biosystems). Relative

abundance of each transcript was determined by normalizing to the housekeeping gene (*GAPDH*) using the $2^{-\Delta\Delta Ct}$ method [9].

In cellulis **Chem-CLIP**. MyoD-fibroblast were differentiated in 100 mm plates for 24 h as described above. After this 24 h differentiation period, 5 µM of **1** (0.1% (v/v) DMSO) was added to cells, and the cells were incubated for an additional 24 h in differentiation medium (48 h total time in differentiation medium; 24 h compound treatment). Cells were irradiated with UV light using a UVP Crosslinker (UV Stratalinker 2400) for 10 min in ice-cold 1× DPBS (Corning). Total RNA was then harvested using a Quick-RNA Mini-Prep (Zymo Research) per the manufacturer's protocol with DNase I treatment. Pull-down of cross-linked RNAs was completed by incubating 15 µg of total RNA with 200 µL of Disulfide Agarose Azide beads (Click Chemistry Tools, #1038) and 90 µL of freshly prepared "click mixture" (10 mM $CuSO_4$; 50 mM THPTA; 250 mM sodium ascorbate pH 7.0, 1:1:1) for 2 h at 37 ˚C. The beads were then washed six times with 1× High Salt Wash Buffer (10 mM Tris-HCl, pH 7.0; 1 mM EDTA, 4 M NaCl, and 0.01% (v/v) Tween-20). The bound RNA was released by adding 200 µL of a mixture of 100 mM TCEP and 300 mM K_2CO_3 (pH 11.0). The solution was incubated for 30 min at 37˚C and then quenched by adding 200 µL of 200 mM iodoacetamide to each sample. The sample was then incubated for an additional 30 min at 37 ˚C, after which the supernatant was collected after centrifugation. The RNA was concentrated *in vacuo* to approximately 100 µL followed by addition of 1.8× volume of CleanXP beads (Beckman Coulter), and RNA was cleaned up according to the manufacturer's protocol. RT-qPCR

was completed as described above. Enrichment was calculated according to the following equation:

$$\text{Enrichment} = 2^\wedge(\Delta C_{t\ \text{After Pull-down}} - \Delta C_{t\ \text{Before Pull-down}})$$

where ΔCt is the difference between the cycle threshold of target gene and the reference gene (GAPDH).

Assessment of target engagement of 1 transcriptome-wide by Chem-CLIP-Seq. DM1 fibroblasts were cultured, differentiated into myotubes, and treated as described above in 100 mm dishes. Cells were washed with 1× DPBS and irradiated with UV light in ice-cold buffer for 15 min. Total RNA was extracted using a Zymo Research Quick-RNA Mini Prep Kit per the manufacturer's recommended protocol with DNase I treatment. Approximately 16 µg of total RNA was captured onto Disulfide Agarose Azide beads (Click Chemistry Tools, #1038) pre-washed with 25 mM Na^+ Hepes, pH 7.0, purified, and eluted as described above. The quality of the RNA after pull-down was assessed by an Agilent 2100 Bioanalyzer Nano chip, affording fragments in the range of 400-1000 nucleotides (Figure S6). Total RNA for samples before and after pull-down was fragmented using an RNA fragmentation module (New England Biolabs), according to the manufacturer's protocol, to obtain RNA samples of 100 – 150 nucleotides.

Fragmentation and RNA length was verified by an Agilent 2100 Bioanalyzer Nano chip, and RNA concentration was quantification by Qubit 2.0 Fluorometer (Invitrogen). Ribosomal RNA was removed from the input sample (200 µg) using NEBNext rRNA depletion module (New England Biolabs) per the manufacturer's recommendations. A sequencing library was generated using NEBNext Ultra II Directional RNA kit (New England Biolabs) per manufacturer's protocols. Briefly, RNA samples were reverse

transcribed with random hexamer primers to generate first strand cDNA, followed by second strand synthesis with dUTP. The obtained cDNA was end repaired, 3' ends – adenylated, followed by adaptor ligation. The second strand was degraded to preserve the RNA strand information using USER enzyme (Uracil-specific excision reagent). The final library was generated by PCR amplification of the cDNA with barcoded Illumina-compatible primers. Samples were loaded onto the NextSeq 500 v2.5 flow cell and sequenced with 2 x 40 bp paired-end chemistry.

As previously described,[1] STAR [10] was used to align all .fastq files to the human genome (Hg38). Then, enriched genes were identified by processing the triplicate of the output (after pull-down) vs the triplicate of the input (before pull-down) ".bam" files of their respective treatment condition with Genrich (v0.6.1, available at https://github.com/jsh58/Genrich) for peak calling ($-\log_{10}(p) > 10$ and False Discovery Rate = 1%). A minimum read count of 10, enrichment (input/output>1) required in all biological replicates, a minimum area under curve (AUC) of 200, fragment length of 400-1000 nucleotides (in accord with the fragment lengths observed after pull-down, as assessed by bioanalyzer), if multiple regions of enrichment were identified for the same transcript, the total reads of each fragment were summed for that transcript, and a minimum Log_2 fold enrichment of 0.8 were applied to filter and remove low-confidence enrichment. Enriched genes identified in both **1**-treated and **14**-treated (control) samples were considered as unselective targets by the diazirine probe and not the binding module. Fold enrichment of RNA fragments identified by Genrich as well as the relative abundance of *DMPK* near the $r(CUG)^{exp}$ was calculated using Samtools (v 1.15.1, https: ://github.com/samtools) [11] that quantifies the number of reads, and RNA seq tracks in *DMPK* were visualized by IGV browser.[12]

The fold enrichment for each biological replicate is calculated as follows. The value reported is the average fold enrichment from three biological replicates:

$$Fold\ of\ enrichment\ for\ each\ biological\ replicate$$
$$= \frac{(\textit{number of reads after pull-down} \ / \ \textit{total number of reads after pull-down})}{(\textit{number of reads before pull-down} \ / \ \textit{total number of reads before pull-down})}$$

In the case where more than one region within a transcript is enriched, fold enrichment was calculated as follows. The value reported is the average fold enrichment from three biological replicates:

$$Fold\ \ enrichment\ for\ each\ biological\ replicate$$
$$= \frac{(\Sigma\ \textit{reads for each region with the fragment after pull-down}) / \ \textit{total number of reads after pull-down})}{(\Sigma\ \textit{reads for each region with the fragment before pull-down}) / \ \textit{total number of reads before pull-down})}$$

Assessment of Allele Selectivity of 1b in DM1 HeLa Cell Model [3]. DM1 HeLa cells expressing WT (0 r(CUG) repeats) and mutant [r(CUG)$_{480}$] alleles were plated into 6-well plates in growth medium (see Cell Culture method above) and grown to ~80% confluency. Compound of interest was added at the indicated concentrations with a final concentration of 0.1% (v/v) DMSO, and the cells were treated for 48 h. Total RNA was harvested using a Zymo Research Quick-RNA Mini Prep Kit per the manufacturer's recommended protocol including the DNase I treatment. Approximately 1000 ng of total RNA was used for reverse transcription (RT) using qScript cDNA synthesis kit (20 µL total reaction volume, Quanta BioSciences) per the manufacturer's procedure. Next, 2 µL of the RT reaction was subjected to qPCR (35 µL total volume) with the corresponding probe (250 nM), forward and reverse (900 nM) primers (Table S3) using SYBR Green

Master Mix (Thermo Fisher Scientific). The qPCR reaction was then aliquoted into three technical replicates (10 μL) and analyzed by a QuantStudio 5, 384-well Block Real-Time PCR System (Applied Biosystems). Relative $r(CUG)_{480}$ transcript abundance was normalized to $r(CUG)_0$ using the $2^{-\Delta\Delta Ct}$ method[9].

RNA Sequencing. Myotubes were differentiated and treated in 6-well plates for 48 h as described above.[2] RNA-Seq libraries were prepared with total RNA using NEB Ultra II Kit with ribosomal RNA depletion and libraries were sequenced in the NextSeq 500 v2 using paired end, 2×75 kits. The raw .fastq files were aligned to the human genome (Hg38) using STAR.[10] Gene expression changes were estimated with Featurecounts [13] and Deseq2.[14] As previously reported,[15] splicing Ψ values were estimated using the version 2 build of hg19 MISO [16]. Splicing events significantly different between DM1 and WT myotubes were determined with a monotonicity test [15], in which minimum ΔΨ was set to 0.1 and minimum Z-value was set to 1.8. To perform downstream analyses, custom Python scripts were written. Composite scores were generated from splicing events where $|\Delta\Psi| > 0.1$, bayes factor > 5 and fisher exact $p < 0.05$ for 7 or more of the 9 pairwise sample comparisons between WT and DM1 myotubes to ensure consistency across replicates.

$r(CUG)^{exp}$–MBNL1 Foci Imaging. RNA-FISH nuclear foci imaging was completed as previously described.[2] Cells were grown in 96-well glass bottom plate (#P96-1.5H-N, Cellvis) covered with Matrigel, differentiated, and treated as described above. After 48 h treatment, cells were washed with 1× DPBS and fixed with 4% (w/v)

paraformaldehyde in 1× DPBS for 10 min at 37 °C. Cells were washed five times with 1× DPBS at 37 °C for 2 min each and permeabilized with 100 µL of 1× DPBS containing 0.1% (v/v) Triton X-100 for 5 min at 37 °C. Cells were then incubated with 100 µL of 30% (v/v) formamide in 2× SSC (saline-sodium citrate) Buffer for 10 min at room temperature and then incubated with 100 µL of the FISH probe (TYE563-2'OMe-$(CAG)_6$, 1 ng/µL, IDT) at 37 °C overnight. Cells were washed again 100 µL of 30% (v/v) formamide in 2× SSC for 30 min at 37 °C and then with 100 µL of 2× SSC Buffer at 37 °C for an additional 30 min.

MBNL1 immunostaining was completed using 1:100 dilution of anti-MBNL1 antibody (#MABE70, MilliporeSigma) in 2× SSC Buffer and incubation at 37 °C for 1 h. Cells were washed three times with 100 µL of 1× DPBS containing 0.1% (v/v) Triton X-100 for 5 min at 37 °C and stained with 1:200 dilution of goat anti-mouse IgG-DyLight 488 conjugate (#A21121, Thermo Scientific) in 2× SSC Buffer at 37 °C for 1 h. After washing three times with 1× DPBS containing 0.1% (v/v) Triton X-100 and twice with 1× DPBS for 5 min at 37 °C, nuclei were stained with DAPI (1 µg/mL, Sigma Aldrich) in 1× DPBS for 5 min at 37 °C. Cells were imaged in 1× DPBS using an Olympus FluoView 1000 confocal microscope at 100× magnification. The number of nuclear foci positive for $r(CUG)^{exp}$ and MBNL1 staining was counted in 40 nuclei/replicate (120 total nuclei counted over three replicates).

***γ*-H2AX Foci Immunostaining.** DNA damage caused by small molecules and the small molecule-bleomycin A5 conjugate was measured by *γ*-H2AX immunofluorescence as previously described.[2] Briefly, cells were grown in 96-well glass bottom plate (#P96-1.5H-N, Cellvis) covered with Matrigel, differentiated, and treated as

described above. After 48h treatment, cells were washed with 1× DPBS and fixed with 4% (w/v) paraformaldehyde in 1× DPBS for 10 min at 37 ˚C. Cells were then washed five times with 1× DPBS for 2 min at 37 ˚C each, and permeabilized with 1× DPBS containing 0.1% (v/v) Triton X-100 for 5 min at 37 ˚C. Cells were washed with 2× SCC Buffer for 30 min at 37 ˚C and then incubated with a 1:500 dilution of anti-γH2AX (#ab26350, Abcam) in 2× SSC Buffer at 37˚C for 1 h. Cells were washed three times with 1× DPBS containing 0.1% (v/v) Triton X-100 for 5 min each at 37˚C, followed by incubation with 1:200 dilution of goat anti-mouse IgG-DyLight 488 conjugate (#A21121, Thermo Scientific) in 2× SSC Buffer at 37 ˚C for 1 h. After washing three times with 1× DPBS containing 0.1% (v/v) Triton X-100 and twice with 1× DPBS for 5 min at 37 ˚C, nuclei were stained with DAPI (1 µg/mL, Sigma Aldrich) in 1× DPBS for 5 min at 37 ˚C. Cells were imaged in 1× DPBS using an Olympus FluoView 1000 confocal microscope at 100× magnification. The number of γ-H2AX foci were counted in 40 nuclei/replicate (120 total nuclei counted over three replicates).

Computational methods

Docking. The NMR identified r(CUG) with the sequence 5'-GACAGCUGAUGUC-3'/5'-GACAGCUGCUGUC-3' (in house model) with a U/U internal loop was used for docking purposes. Prior to grid calculations, polar hydrogen atoms and Gasteiger charges were added to the receptor (RNA) using AutoDock Tools 1.5.6 and MG Tools of AutoDock Vina [17] and saved in pdbqt format. The 3D structure of **1a** was created with OBabel [18] from SMILES input file and geometry optimized with general AMBER force field (GAFF) [19] in 5000 cycles prior to further processing for docking. Polar hydrogen atoms and Gasteiger charges [20] were added to the small molecules as described above for the RNA. The Grid file was then generated from ligand and receptor pdbqt files, applying the prepare_gpf4.py script; autogrid4 and prepare_dpf4.py were used to prepare the docking parameter file. AUTODOCK-GPU [21] was then used to dock the ligand against the receptor.

MD Simulations, Clustering, and Free Energy Calculations. To further investigate the binding mode of **1a**, a combination of MD simulations, cluster analysis, and free energy calculations was used, including explicit water molecules and salt ions.

*Parametrization of **1a**.* GAFF was used to assign the bonds, angles, torsions, improper torsions, and Lennard-Jones parameters using the Antechamber and Parmchk programs.[19, 22] In order to extract the charges, **1a** was geometry optimized at the quantum-mechanical (QM) HF/6-31G* level using Gaussian 09 [23] consistent with the AMBER force fields. Then atomic charges were determined by restrained electrostatic potential (RESP) charge fitting.[24] RED (RESP ESP charge Derive) program was used to generate the final charges.[25] Full description of atom types and charges are represented in Table S5.

MD simulations. Two docked poses obtained from docking studies were used as initial structures for MD simulations. Simulations were carried out with the AMBER 16 [26] simulation package using the PARM99 force field [27] with revised χ [28] and α/γ [29] torsional parameters. Each system was first neutralized with Na^+ ions [30] and then solvated with TIP3P water molecules [31] in a truncated octahedral box with periodic boundary conditions extended to 10 Å using the LEAP module of AMBER 16.

The structures were minimized with the sander module each in two steps. Positional restraints (10 kcal mol^{-1} $Å^{-2}$) were applied on the RNA+ligand complex in the first step of minimization with 5000 steps of steepest-descent algorithm. A second round of minimization with 5000 steps of conjugate-gradient algorithm with no restraints was then completed. Minimization was followed by an equilibration protocol first in constant volume with restraints on the RNA molecule (10 kcal mol^{-1} $Å^{-2}$) and gradually increasing the temperature up to 300 K for several nanoseconds using the Langevin thermostat. A second round of equilibration was performed at constant pressure with constant temperature at 300 K and pressure coupling of 1.0 ps^{-1},[32] gradually removing the constraints on the solute.

After minimization and equilibration, MD simulation under constant pressure (NPT) with a 2 fs time step was performed for each system with isotropic positional scaling. The reference pressure was set to 1 atm with a pressure relaxation time of 2 ps. SHAKE [33] was turned on for constraining bonds involving hydrogen atoms. An atom-based long-range cutoff of 10.0 Å was used in the production runs. The reference temperature was set to 300 K. The Particle Mesh Ewald (PME) method was used to handle the electrostatics [34] and the Langevin thermostat [35] was applied with a coupling

constant γ = 1.0 ps^{-1}. Simulations were performed using the PMEMD.CUDA implementation of AMBER 16.

Each system was simulated for 1 µsec. Trajectories obtained from MD simulations were combined and then clustered using average linkage algorithm implemented in CPPTRAJ module of AmberTools. The 7 most populated clusters were used for free energy calculations.

RISM calculations. Binding free energies of 7 clusters were calculated using the Reference Interaction Site Model (RISM) [36] approach implemented in CPPTRAJ (MMPBSA.py.MPI). The Kovalenko–Hirata (KH) closure [37] was utilized for the RISM calculations. Table S6 shows the free energies calculated for the seven most populated clusters with a population of over 500 conformations.

Model building of r(CUG)$_{12}$. After identifying the most stable bound state with the lowest binding energy, the 1×1 U/U internal loop was excised from the model. A series of rotation and aligning commands using the 3DNA (script 1) [38] was used to generate an RNA model with five binding sites. The Xleap module of Amber was then used to minimize the energy of the model construct and the O3'-P bonds as 3DNA overlong the phosphodiester bond (Figure S5C). A 600 ns MD simulation in explicit water was performed on the duplex structure, which was stable over the course of the simulation. Then the loop structure from the pdb database, 2oj7, was used to create the loop model. The same process of combining 3DNA with Xleap was used to create and energy minimize the hairpin model (Figure S5D).

Script 1. 3DNA script to create an RNA model with multiple copies of the CUG motif.

```
x3dna-dssr tasks -i=model.pdb --frame-pair=last -o=model1-ref-last.pdb
x3dna-dssr fiber --seq=GG --rna-ds -o=conn.pdb
```

```
x3dna-dssr tasks -i=conn.pdb --frame-pair=first --remove-pair -o=ref-conn.pdb

x3dna-dssr tasks --merge-file='model1-ref-last.pdb ref-conn.pdb' -o=temp1.pdb

x3dna-dssr tasks -i=temp1.pdb --frame-pair=last --remove-pair -o=temp2.pdb

x3dna-dssr tasks -i=model.pdb --frame-pair=first -o=model1-ref-first.pdb

x3dna-dssr tasks --merge-file='temp2.pdb model1-ref-first.pdb' -o=duplicate-model.pdb
```

Synthetic Methods

Abbreviations: $CDCl_3$, chloroform-d; CD_3OD, methanol-d_4; DIPEA, N,N-diisopropylethylamine; DCM, dichloromethane; DMF, *N*,*N*-dimethylformamide; DMSO, dimethyl sulfoxide; EDC, *N*-ethyl-*N*'-(3- dimethylaminopropyl)carbodiimide hydrochloride; EDTA, ethylenediaminetetraacetic acid; Et_3N, triethylamine; EtOAc, ethyl acetate; EtOH, ethanol; HCl, hydrochloric acid H_2O, water; HATU, hexafluorophosphate azabenzotriazole tetramethyl uronium; HOAt, 1-hydroxy-7-azabenzotriazole; HOBt, 1-hydroxybenzotriazole; HPLC, high performance liquid chromatography; LC-MS, liquid chromatography-mass spectrometry; LDA, lithium diisopropylamide; PEG, polyethylene glycol; MALDI, matrix-assisted laser desorption/ionization; MeOH, methanol; NaCl, sodium chloride; Na_2SO_4, sodium sulfate; NaH_2PO_4, sodium phosphate monobasic; $NaHCO_3$, sodium bicarbonate; NMR, nuclear magnetic resonance; SiO_2, silica; TFA, trifluoroacetic acid; TLC, thin layer chromatography.

General. All reagents and solvents used for chemical synthesis were purchased from commercial suppliers and were used without further purification unless mentioned otherwise. Reactions were monitored with an Agilent 1260 Infinity LC system coupled to an Agilent 6230 TOF (HR-ESI) equipped with a Poroshell 120 EC-C18 column (Agilent, 50 mm × 4.6 mm, 2.7 µm) or by TLC. Products were purified by Isolera One flash chromatography system (Biotage) using pre-packed silica irregular 40-60 µm 60A column (Claricep Flash, Agela Technologies) or by HPLC (Waters 2489 pump and 1525 detector) using a SunFire Prep C18 OBD 5 µm column (19 × 150 mm) with a flow rate of

5 mL/min and typically a gradient from 0% to 100% solvent B (100% MeOH + 0.1% (v/v) TFA) in solvent A (H_2O + 0.1% (v/v) TFA) over 60 min. Compound purity was analyzed by HPLC using a SunFire C18 3.5 µm column (4.6 × 150 mm) with the flow rate of 1 mL/min and a gradient from 0% to 100% solvent B (100% MeOH + 0.1% (v/v) TFA) in solvent A (H_2O + 0.1% (v/v) TFA) over 60 min. NMR spectra for compound characterization were measured by a 400 UltraShieldTM (Bruker) (400 MHz for ^1H and 100 MHz for ^{13}C) or an AscendTM 600 (Bruker) (600 MHz for ^1H and 150 MHz for ^{13}C). Chemical shifts are expressed in ppm relative to trimethylsilane (TMS) for ^1H and residual solvent for ^{13}C as internal standards. Coupling constants (J values) are reported in Hz. Mass spectra were recorded on a 4800 Plus MALDI TOF/TOF Analyzer (Applied Biosystems) with α-cyano-4-hydroxycinnamic acid matrix

Peptide coupling general procedure for aromatic amines

In a 4 mL brown vial containing 3-(3-(but-3-yn-1-yl)-3H-diazirin-3-yl)propanoic acid (1.5 eq.) in DMF (0.5 mL), amine derivative (1.0 eq.), HATU (1.5 eq.), HOAt (1.5 eq.) and DIPEA (3.0 eq.) were added. The reaction mixture was stirred at 40˚C for 4 h to overnight. DMF was evaporated and the remaining crude product was dissolved in DCM and washed with water (3 × 1 mL). The organic layer was dried *in vacuo* and purified by either flash chromatography or HPLC.

Peptide coupling general procedure for aliphatic amines

In a 4 mL brown vial containing 3-(3-(but-3-yn-1-yl)-3H-diazirin-3-yl)propanoic acid (1.5 eq.) in DCM (0.5 mL), amine derivative (1.0 eq.), EDC (1.5 eq.), HOBt (1.5 eq.) and DIPEA (3.0 eq.) were added. The reaction mixture was stirred at room temperature

for 4 h to overnight. The crude mixture was washed with water (3 × 1 mL). The organic layer was dried *in vacuo* and purified by either flash chromatography or HPLC.

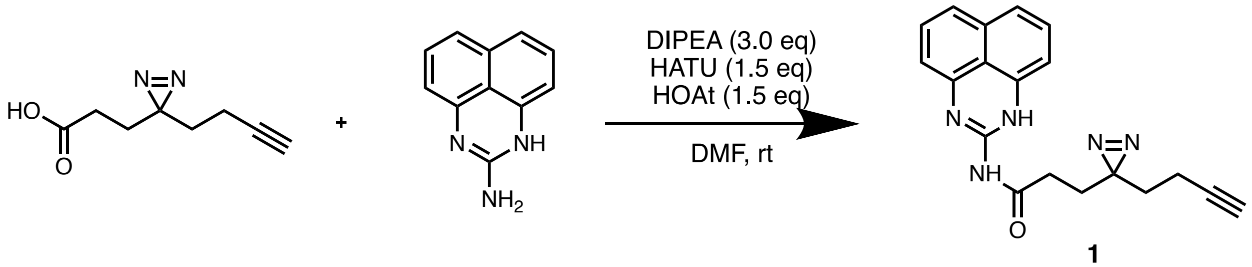

1

Scheme S1. Synthesis of compound **1**

Compound 1. 3-(3-(but-3-yn-1-yl)-3*H*-diazirin-3-yl)-*N*-(1*H*-perimidin-2-yl)propanamide.

Peptide coupling general procedure for aromatic amines. Green-yellow oil (Yield: 80 %). ^{1}H NMR (400 MHz, CD_3OD) δ (ppm) 7.13(t, J=4 Hz, 2H), 7.07(d, J=4 Hz, 2H), 6.48(d, J=4 Hz, 2H), 2.28(t, J=1.6 Hz, 1H), 2.24(t, J=4 Hz, 2H), 2.05(td, J=1.6-4 Hz, 2H), 1.82(t, J=5 Hz, 2H), 1.64(t, J=5 Hz, 2H); ^{13}C NMR (100 MHz, CD_3OD) δ (ppm) 178.90, 150.76, 136.69, 129.22, 121.47, 120.38, 108.66, 83.62, 70.34, 34.44, 32.56, 29.02, 28.82, 13.85; HR-MS: Calculated for $[C_{19}H_{18}N_5O_1]^+$, 332.1511; found 332.1508.

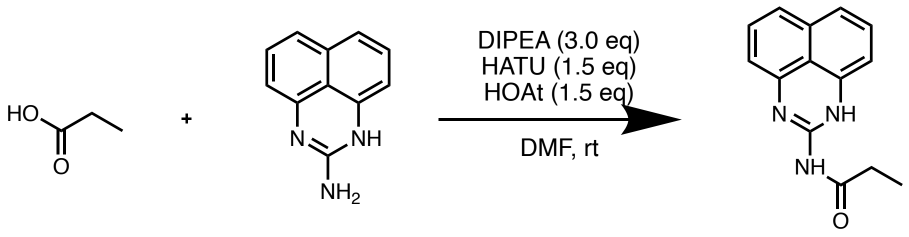

1a

Scheme S2. Synthesis of compound **1a**

Compound 1a. *N*-(1*H*-perimidin-2-yl)propionamide

Peptide coupling general procedure for aromatic amines. Yellow oil (Yield: 100 %). ^{1}H NMR (400 MHz, DMSO-d_6) δ (ppm) 7.27-7.18(m, 4H), 6.75(dd, J=1.6-4 Hz, 2H), 2.47(q, J=8 Hz, 2H), 1.08(t, J=8 Hz, 3H); ^{13}C NMR (100 MHz, DMSO-d_6) δ (ppm) 176.18, 147.81, 134.45, 128.37, 119.92, 119.41, 108.02, 29.48, 8.50. HR-MS: Calculated for $[C_{14}H_{14}N_3O]^+$, 240.1137; found 240.0959.

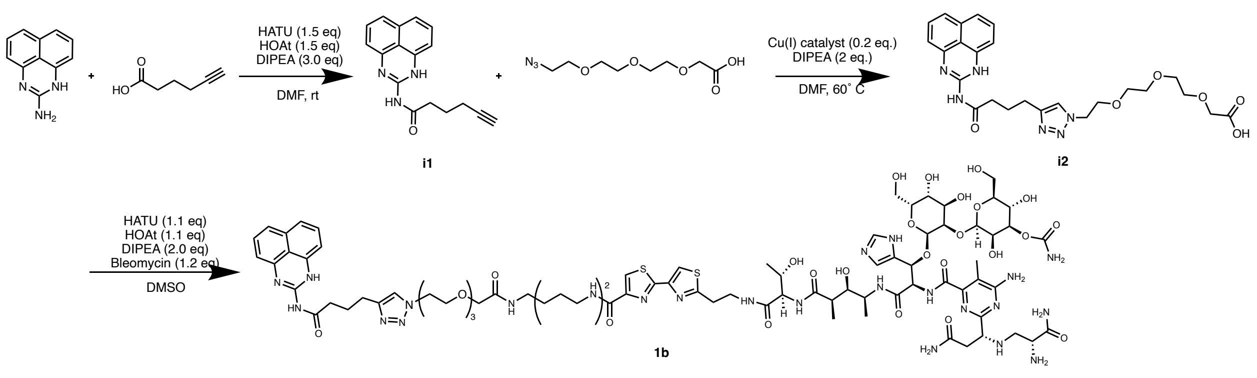

Scheme S3. Synthesis of compound **1b**

Synthesis of i1. In a 4 mL glass vial containing 5-hexynoic acid (11.2 mg, 0.1 mmol, 1.0 eq) in DMF (0.5 mL), 1H-perimidin-2-amine hydrobromide hydrate (28.2 mg, 0.1 mmol, 1.0 eq), HATU (45.6 mg, 0.120 mmol, 1.5 eq), HOAt (16.3 mg, 0.120 mmol, 1.5 eq) and DIPEA (51.0 µL, 0.3 mmol, 3.0 eq.) were added. The reaction mixture was stirred at room temperature for 4 h to overnight. DMF was evaporated, and the remaining crude product was dissolved in DCM and washed with water (3 × 1 mL). The organic layer was dried *in vacuo* and purified via flash chromatography (20 mg, 70 % yield). ^{1}H NMR (400 MHz, $CDCl_3$) δ (ppm) 7.20-7.10(m, 4H), 6.48 (d, J=8 Hz, 2H), 2.50(t, J=8 Hz, 2H), 2.21(td, J=1.6-4 Hz, 2H), 1.92-1.82(m, 3H); ^{13}C NMR (100 MHz, $CDCl_3$) δ (ppm) 177.11, 149.55, 135.47, 128.33, 121.03, 119.91, 82.95, 69.60, 36.18, 23.62, 17.85.

Synthesis of i2. In a 4 mL glass vial containing the **i1** (4.1 mg, 0.015 mmol, 1.0 eq) and 11-azido-3,6,9-trioxaundecanoic acid (3.8 mg, 0.016 mmol, 1.1 eq) in DMF (0.5 mL), (1.8 mg, 0.003 mmol, 0.2 eq), Cu(I) catalyst (0.2 eq.) and DIPEA (5.0 µL, 0.03 mmol, 2.0 eq) were added, and the reaction was stirred at 60 °C for 2 h. The reaction mixture was diluted with MeOH and purified by HPLC (18 mg, 65 % yield). ^1H NMR (400 MHz, CD_3OD) δ (ppm) 7.99(s,1H), 7.41-7.26(m, 4H), 6.86(d, J=8 Hz, 2H), 4.60-4.52(m, 2H), 4.12(s, 2H), 3.89(s, 2H), 3.65-3.57(m, 8H), 2.83(s, 2H), 2.66(s, 2H), 2.10(s, 2H); ^{13}C NMR (100 MHz, CD_3OD) δ (ppm) 177.00, 149.88, 136.05, 133.43, 129.43, 129.28, 123.31, 122.41, 119.91, 109.56, 107.92, 71.73, 71.54, 71.47, 71.39, 70.33, 51.48, 36.85, 25.01.

Synthesis of 1b. In a 4 mL glass vial, **i2** (1.5 mg, 0.003 mmol, 1.0 eq) was activated with HATU (1.2 mg, 0.003 mmol, 1.1 eq), HOAt (0.4 mg, 0.003 mmol, 1.1 eq), DIPEA (1 µL, 0.006 mmol, 2.0 eq) in DMF (0.5 mL) for 30 min at room temperature. Then, copper-coordinated bleomycin (5.3 mg, 0.004 mmol, 1.2 eq) was added, and the reaction was stirred at room temperature overnight. The reaction was then HPLC purified by first using 0.1 mM EDTA in water (pH 6.3) for 15 min followed by 100% water for 15 min and then a 15-50% gradient of MeOH/water + 0.1% (v/v) TFA over 1 h (10 % yield). ^1H NMR (600 MHz, CD_3OD) δ (ppm) 8.91(m, 2H), 8.21(m, 1H), 8.09(s, 1H), 7.89(s, 1H), 7.39(m, 2H), 7.32(m, 2H), 6.89(dd, J=1-7 Hz, 2H), 5.48(m, 2H), 5.21(m, 5H), 4.81(m, under solvent peak, 1H), 4.53(m, 4H), 4.32(d, J=4 Hz, 1H), 4.18-3.95(m, 8H), 3.90(t, J=5 Hz, 3H), 3.85-3.58(m, 15H), 3.54(t, J=6 Hz, 3H), 3.47(dd, J=7-12 Hz, 1H), 3.26(m, 3H), 3.15-2.99(m, 5H), 2.91(dd, J=8-16 Hz, 1H), 2.83(t, J=7 Hz, 2H), 2.67(t, J=7 Hz, 2H), 2.58(m, 1H), 2.28(m, 3H), 2.05(m, 4H), 1.65(m, 4H), 1.36-1.09(m, 9H); ^{13}C NMR (150 MHz, CD_3OD) δ (ppm) 177.09, 172.97, 170.83, 164.37, 164.19, 161.19, 160.93, 160.67, 160.42,

151.13, 149.96, 149.39, 147.81, 135.97, 132.77, 129.45, 125.60, 124.47, 123.55, 119.80, 119.67, 117.89, 115.98, 114.08, 109.55, 75.70, 71.95, 71.35, 71.21, 70.26, 51.46, 49.57, 46.42, 40.18, 38.92, 37.08, 36.79, 33.56, 27.81, 27.52, 25.22, 24.96, 24.53, 20.25, 15.34, 14.25, 11.88. MS (m/z): calculated $C_{82}H_{117}N_{25}O_{26}S_2$ $[M+1]^+$: 1932.81, found: 1932.9; $[M+23]^+$: 1954.79, found: 1954.8.

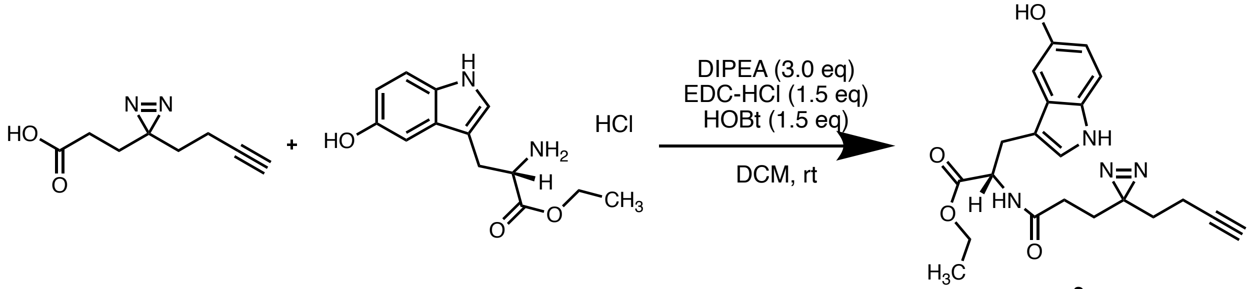

2

Scheme S4. Synthesis of compound **2**

Compound F2. ethyl (*S*)-2-(3-(3-(but-3-yn-1-yl)-3*H*-diazirin-3-yl)propanamido)-3-(5-hydroxy-1*H*-indol-3-yl)propanoate

Peptide coupling general procedure for aliphatic amines. Brown oil (Yield: 100 %). ^{1}H NMR (400 MHz, DMSO-*d₆*) δ (ppm) 10.51(s, 1H), 8.60(s, 1H), 8.27(d, J=8 Hz, 1H), 7.10(d, J=8 Hz, 1H), 7.01(s, 1H), 6.76(s, 1H), 6.57(d, J=8 Hz, 1H), 4.41(q, J= 8 Hz, 1H), 4.00(td, J=4-8 Hz, 2H), 3.00(dd, J=8-16 Hz, 1H), 2.89(dd, J=8-12 Hz, 1H), 2.82-2.76(m, 1H), 1.98-1.89(m, 4H), 1.60-1.47(m, 4H), 1.07(t, J=8 Hz, 3H); ^{13}C NMR (150 MHz, DMSO-d6): δ= 172.05, 170.96, 150.37, 130.70, 127.80, 124.21, 111.80, 111.36, 108.46, 101.99, 83.26, 71.78, 60.44, 53.10, 31.34, 30.77, 29.20, 28.26, 28.15, 13.99, 12.70. HR-MS: Calculated for $[C_{21}H_{25}N_4O_4]^+$, 397.1876; found 397.1879.

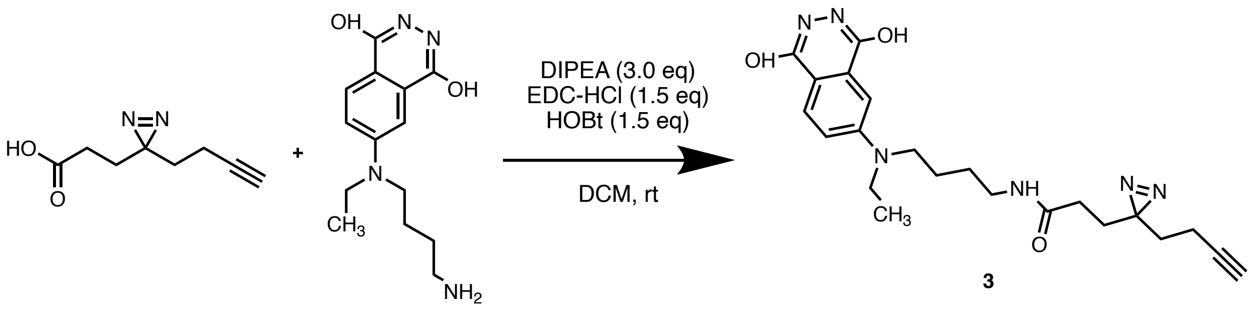

Scheme S5. Synthesis of compound **3**

Compound 3. 3-(3-(but-3-yn-1-yl)-3*H*-diazirin-3-yl)-*N*-(4-((1,4-dihydroxyphthalazin-6-yl)(ethyl)amino)butyl)propanamide

Peptide coupling general procedure for aliphatic amines. Dark green oil (Yield: 42 %). 1H NMR (400 MHz, DMSO-d_6) δ (ppm) 11.13(s, 2H), 7.88(t, J=4 Hz, 1H), 7.82(d, J=8 Hz, 1H), 7.16(dd, J=4-8 Hz, 1H), 7.01(s, 1H), 3.47(dd, J=4-12 Hz, 2H), 3.39(m, 2H), 3.07(dd, J=4-12 Hz, 2H), 2.81(t, J=4 Hz, 1H), 1.97(td, J=4-8 Hz, 2H), 1.88(dd, J=1.6-8 Hz, 2H), 1.63(dd, J=1.6-8 Hz, 2H), 1.54(t, J=8 Hz, 3H), 1.50-1.42(m, 2H), 1.13(t, J=4 Hz, 2H), 0.88-0.84(m, 3H); ^{13}C NMR (150 MHz, DMSO-d6): δ= 170.66, 158.44, 158.20, 150.38, 128.70, 127.10, 116.68, 115.12, 103.27, 83.25, 71.79, 49.39, 44.52, 40.43, 38.30, 31.51, 29.58, 28.34, 28.19, 26.63, 24.28, 12.72, 11.90; HR-MS: Calculated for $[C_{22}H_{29}N_6O_3]^+$, 425.2301; found 425.2305.

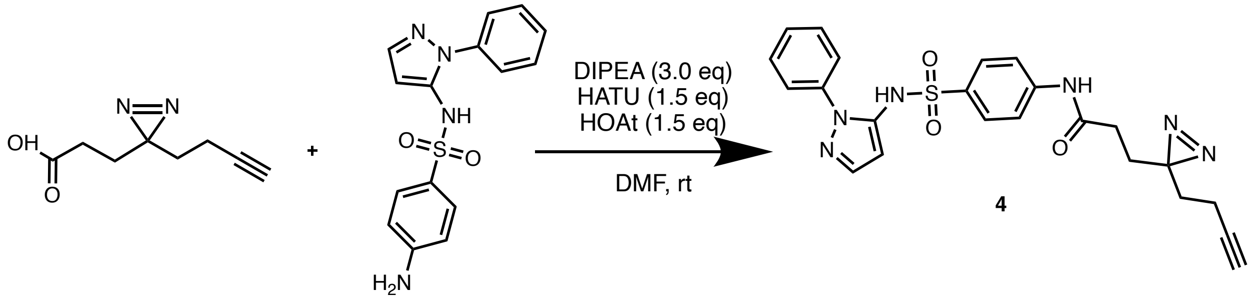

Scheme S6. Synthesis of compound **4**

Compound 4. 3-(3-(but-3-yn-1-yl)-3*H*-diazirin-3-yl)-*N*-(4-(*N*-(1-phenyl-1*H*-pyrazol-5-yl)sulfamoyl)phenyl)propanamide

Peptide coupling general procedure for aromatic amines. Clear yellow oil (Yield: 100 %). ^{1}H NMR (400 MHz, CD_3OD) δ (ppm) 7.53-7.59(m, 2H), 7.54-7.49(m, 3H), 7.40-7.34(m, 3H), 7.23-7.19(m, 2H), 6.06(d, J=4 Hz, 1H), 2.23-2.18(m, 3H), 1.84(t, J=8 Hz, 2H), 1.71(t, J=8 Hz, 1H), 1.66-1.54(m, 4H); ^{13}C NMR (150 MHz, DMSO-d_6): δ= 173.45, 170.72, 143.33, 139.72, 138.34, 128.89, 128.16, 127.64, 124.51, 118.87, 112.62, 83.39, 72.00, 31.59, 31.48, 28.32, 27.99, 12.76. HR-MS: Calculated for $[C_{23}H_{23}N_6O_3S]^+$, 463.1552; found 463.1548.

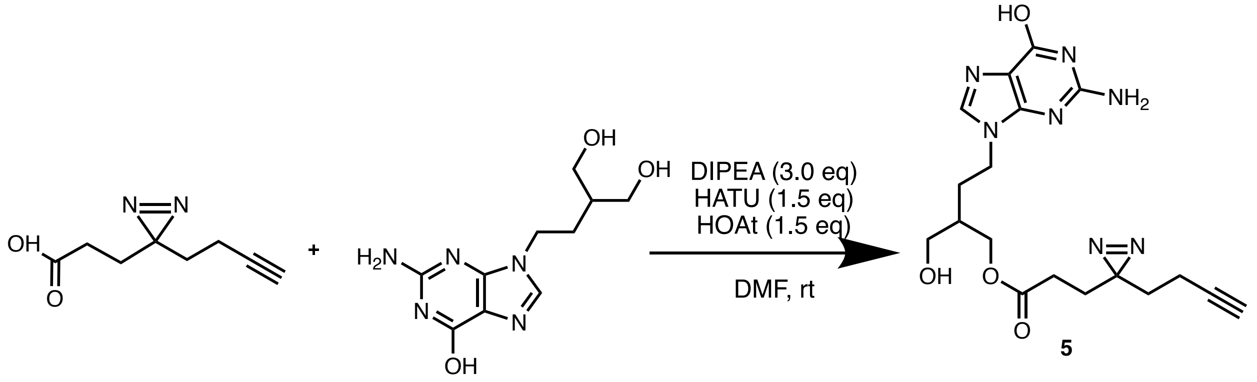

Scheme S7. Synthesis of compound **5**

Compound 5. 4-(2-amino-6-hydroxy-9*H*-purin-9-yl)-2-(hydroxymethyl)butyl 3-(3-(but-3-yn-1-yl)-3*H*-diazirin-3-yl)propanoate

Peptide coupling general procedure for aromatic amines. Yellow oil (Yield: 30 %). ^{1}H NMR (400 MHz, CD_3OD) δ (ppm) 7.65-7.60(m, 1H), 4.38-4.14(m, 4H), 3.67-3.55(m, 2H), 2.27(t, J=4 Hz, 1H), 2.22-2.11(m, 2H), 2.06-1.98(m, 2H), 1.78(td, J=4-8 Hz, 2H), 1.61(t, J=8 Hz, 2H), 1.41-1.37(m, 3H); ^{13}C NMR (150 MHz, DMSO-d6): δ= 171.91, 156.98, 153.43, 151.24, 137.54, 116.75, 83, 71.78, 64.29, 63.53, 37.53, 31.32, 29.07, 27.91, 27.40, 22.16, 18.94, 12.66. HR-MS: Calculated for $[C_{18}H_{24}N_7O_4]^+$, 402.1890; found 402.1885.

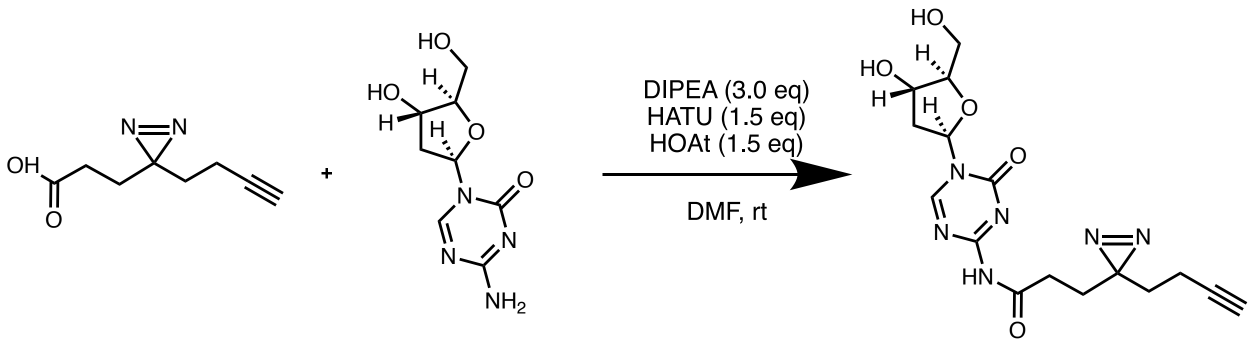

6

Scheme S8. Synthesis of compound **6**

Compound 6. 3-(3-(but-3-yn-1-yl)-3*H*-diazirin-3-yl)-*N*-(5-((2*R*,4*S*,5*R*)-4-hydroxy-5-(hydroxymethyl)tetrahydrofuran-2-yl)-4-oxo-4,5-dihydro-1,3,5-triazin-2-yl)propanamide

Peptide coupling general procedure for aromatic amines. Yellow oil (Yield: 27 %). ^{1}H NMR (400 MHz, CD_3OD) δ (ppm) 8.38(s, 1H), 6.09(t, J=8 Hz, 1H), 4.41-4.32 (m, 2H), 4.14(q, J=4 Hz, 1H), 3.82(t, J=4 Hz, 1H), 2.32-2.24(m, 2H), 2.25-2.18(m, 2H), 2.05-2.00(m, 2H), 1.84-1.75(m, 2H), 1.66-1.58(m, 3H); ^{13}C NMR (150 MHz, DMSO-d_6): δ= 172.15, 171.95, 166.33, 156.55, 85.99, 84.60, 83.87, 72.43, 70.65, 61.68, 38.20, 31.70, 28.40, 28.20, 27.80, 13.06. HR-MS: Calculated for $[C_{16}H_{21}N_6O_5]^+$, 377.1573; found 377.1570.

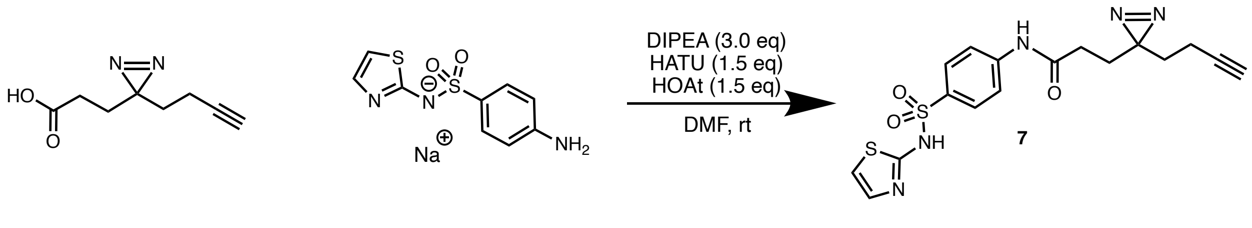

7

Scheme S9. Synthesis of compound 7

Compound 7. 3-(3-(but-3-yn-1-yl)-3*H*-diazirin-3-yl)-*N*-(4-(*N*-(thiazol-2-yl)sulfamoyl)phenyl)propanamide

Peptide coupling general procedure for aromatic amines. Yellow oil (Yield: 22 %). ^{1}H NMR (400 MHz, CD_3OD) δ (ppm) 7.81(d, J=8 Hz, 2H), 7.69(d, J=8 Hz, 2H), 7.08(d, J=8 Hz, 1H), 6.70(d, J=8 Hz, 1H), 2.22(t, J=8 Hz, 2H), 2.11(t, J=8 Hz, 2H), 1.81(t, J=8 Hz, 1H), 1.75(t, J=8 Hz, 2H), 1.63 (t, d, J=8 Hz, 2H); ^{13}C NMR (150 MHz, DMSO-d6): δ= 173.39, 170.36, 142.33, 136.33, 127.00, 118.62, 112.53, 108.20, 83.19, 71.69, 31.53, 31.48, 30.57, 27.99, 12.69. HR-MS: Calculated for $[C_{17}H_{18}N_5O_3S_2]^+$, 404.0851; found 404.0855.

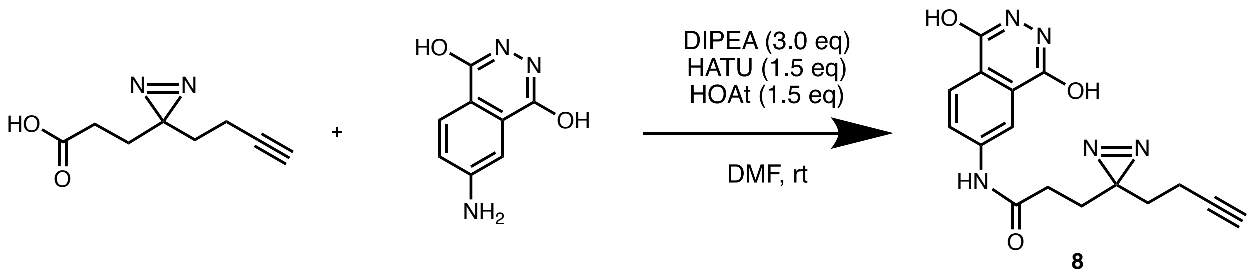

Scheme S10. Synthesis of compound **8**

Compound 8. 3-(3-(but-3-yn-1-yl)-3*H*-diazirin-3-yl)-*N*-(1,4-dihydroxyphthalazin-6-yl)propanamide

Peptide coupling general procedure for aromatic amines. Clear yellow oil (Yield: 25 %). ^{1}H NMR (400 MHz, CD_3OD) δ (ppm) 8.45(d, J=4 Hz, 1H), 8.14(d, J=8 Hz, 1H), 8.03(dd, J=4-8 Hz, 1H), 2.31-2.26(m, 3H), 2.06(td, J=1.8-8 Hz, 2H), 1.89(t, J=8 Hz, 2H), 1.67(t, J=8 Hz, 2H). ^{13}C NMR (150 MHz, DMSO-d6): δ= 170.62, 159.68, 153.03, 145.63, 130.74, 125.33, 120.40, 113.72, 106.80, 83.28, 71.90, 31.56, 31.38, 30.68, 28.30, 12.74. HR-MS: Calculated for $[C_{16}H_{16}N_5O_3]^+$, 326.1253; found 326.1249.

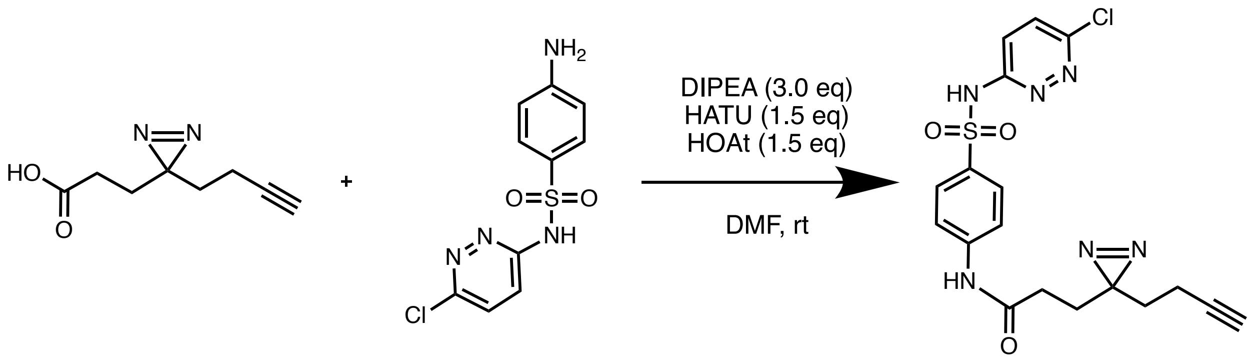

Scheme S11. Synthesis of compound **9**

Compound 9. 3-(3-(but-3-yn-1-yl)-3*H*-diazirin-3-yl)-*N*-(4-(*N*-(6-chloropyridazin-3-yl)sulfamoyl)phenyl)propanamide

Peptide coupling general procedure for aromatic amines. Yellow oil (Yield: 79 %). ^{1}H NMR (600 MHz, CD_3OD) δ (ppm) 7.89(d, J=9 Hz, 2H), 7.73(d, J=9 Hz, 2H), 7.70-7.61(m, 2H), 2.26(t, J=3 Hz, 1H), 2.22(t, J=7 Hz, 2H), 2.04(td, J=3-8 Hz, 2H), 1.84(t, J=8 Hz, 2H), 1.63(t, J=7 Hz, 2H); ^{13}C NMR (150 MHz, CD_3OD): δ= 172.99, 155.74, 144.51, 129.46, 120.41, 83.57, 70.33, 33.44, 31.84, 29.29, 28.83, 13.82. HR-MS: Calculated for $[C_{18}H_{18}ClN_6O_3S]^+$, 433.0850; found 433.0764.

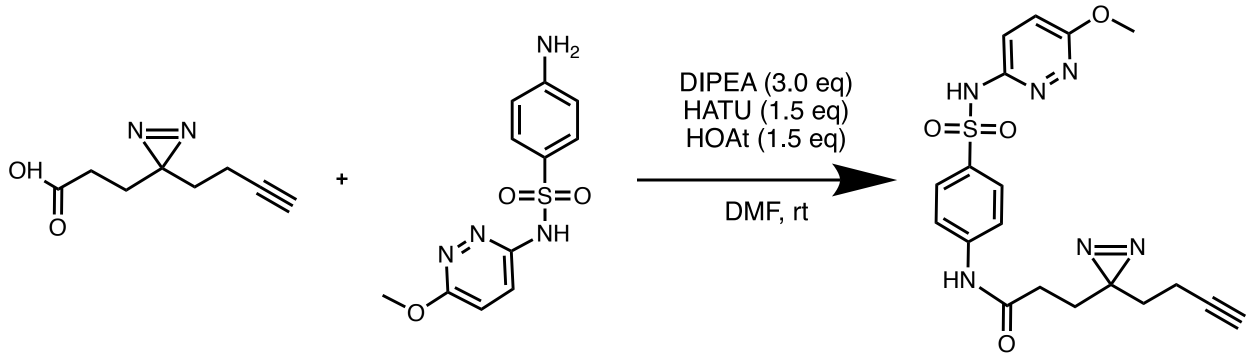

Scheme S12. Synthesis of compound **10**

Compound 10. 3-(3-(but-3-yn-1-yl)-3*H*-diazirin-3-yl)-*N*-(4-(*N*-(6-methoxypyridazin-3-yl)sulfamoyl)phenyl)propanamide

Peptide coupling general procedure for aromatic amines. Yellow oil (Yield: 53 %). ^{1}H NMR (400 MHz, CD_3OD) δ (ppm) 7.85(dt, J=3-9 Hz, 2H), 7.71(m, 3H), 7.25(d, J=9 Hz, 1H), 3.91(s, 3H), 2.26(t, J=3 Hz, 1H), 2.22(dd, J=8-9 Hz, 2H), 2.04(td, J=3-8 Hz, 2H), 1.84(dd, J=7-8 Hz, 2H), 1.64(t, J=7 Hz, 2H); ^{13}C NMR (150 MHz, CD_3OD): δ= 172.93, 160.44, 154.19, 143.72, 138.10, 128.95, 128.67, 126.84, 120.21, 83.58, 70.33, 55.35, 33.45, 31.84, 29.34, 28.84, 13.83. HR-MS: Calculated for $[C_{19}H_{21}N_6O_4S]^+$, 429.1345; found 429.1048.

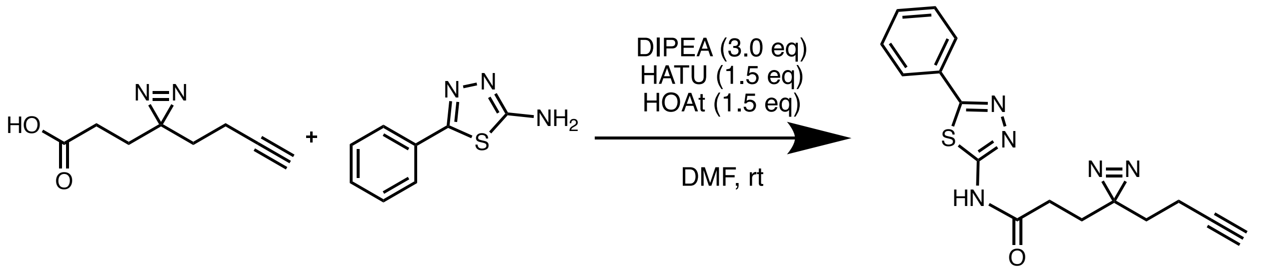

11

Scheme S13. Synthesis of compound **11**

Compound 11. 3-(3-(but-3-yn-1-yl)-3*H*-diazirin-3-yl)-*N*-(5-phenyl-1,3,4-thiadiazol-2-yl)propanamide

Peptide coupling general procedure for aromatic amines. Clear yellow oil (Yield: 55 %). ^{1}H NMR (400 MHz, $CDCl_3$) δ (ppm) 8.02-7.97(m, 2H), 7.54-7.46(m, 3H), 2.69(t, J=8 Hz, 2H), 2.07-2.05(m, 2H), 2.03-1.99(m, 2H), 1.96(t, J=4 Hz, 1H), 1.77(t, J=8 Hz, 2H); ^{13}C NMR (150 MHz, DMSO-d6): δ= 172.15, 171.95, 166.33, 156.55, 85.99, 84.60, 83.87, 72.43, 70.65, 61.68, 38.20, 31.70, 28.40, 28.20, 27.80, 13.06. HR-MS: Calculated for $[C_{16}H_{16}N_5OS]^+$, 326.1076; found 326.1073.

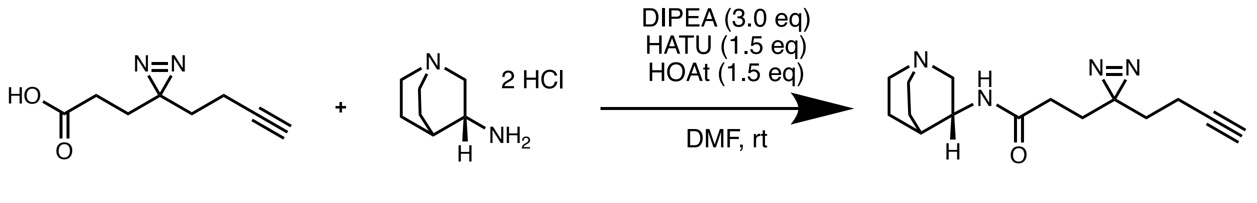

Scheme S14. Synthesis of compound **12**

Compound 12. (*R*)-3-(3-(but-3-yn-1-yl)-3*H*-diazirin-3-yl)-*N*-(quinuclidin-3-yl)propanamide

Peptide coupling general procedure for aliphatic amines. Colorless oil (Yield: 100 %). ^{1}H NMR (400 MHz, $CDCl_3$) δ (ppm) 7.76(d, J=8 Hz, 1H), 4.47(s, 2H), 4.38(dd, J=8-16 Hz, 1H), 3.62-3.52(m, 1H), 3.52-3.41(m, 2H), 3.31-3.11(m, 3H), 2.37(q, J=4 Hz, 1H), 2.30-2.19(m, 1H), 2.07-1.99(m, 6H), 1.85-1.80(m, 2H), 1.64(t, J=8 Hz, 2H); ^{13}C NMR (150 MHz, DMSO-d6): δ= 171.15, 83.24, 71.85, 53.62, 51.94, 45.71, 45.11, 43.79, 31.46, 29.41, 28.32, 27.99, 23.96, 21.43, 12.72. HR-MS: Calculated for $[C_{15}H_{23}N_4O]^+$, 275.1871; found 275.1874.

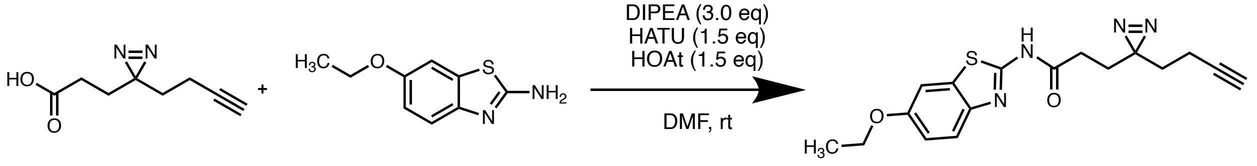

Scheme S15. Synthesis of compound **13**

Compound 13. 3-(3-(but-3-yn-1-yl)-3*H*-diazirin-3-yl)-*N*-(6-ethoxybenzo[*d*]thiazol-2-yl)propanamide

Peptide coupling general procedure for aromatic amines. Clear yellow oil (Yield: 73 %). ^{1}H NMR (400 MHz, $CDCl_3$) δ (ppm) 7.63(d, J=8 Hz, 1H), 7.28(d, J=4 Hz, 1H), 7.06(dd,

J=4-8 Hz, 1H), 4.09(q, J=8 Hz, 2H), 2.27(t, J=8 Hz, 2H), 2.04-1.91(m, 5H), 1.63(t, J=8 Hz, 2H), 1.45(t, J=8 Hz, 2H); ^{13}C NMR (150 MHz, DMSO-d6): δ= 170.66, 155.79, 155.39, 142.55, 132.77, 121.18, 115.30, 105.40, 83.24, 71.88, 63.66, 31.45, 29.44, 28.19, 27.39, 14.77, 12.72; HR-MS: Calculated for $[C_{17}H_{19}N_4O_2S]^+$, 343.1229; found 343.1232.

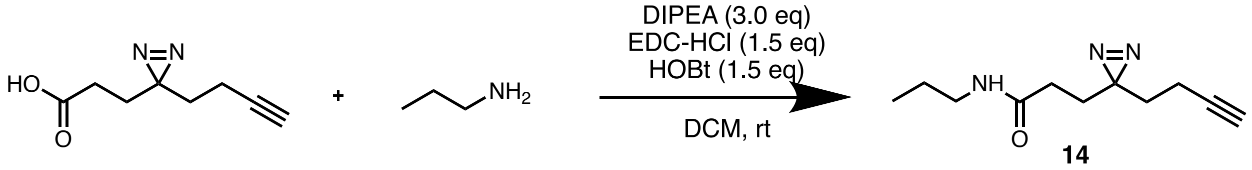

Scheme S16. Synthesis of compound **14**

Compound 14. 3-(3-(but-3-yn-1-yl)-3H-diazirin-3-yl)-N-propylpropanamide.

Peptide coupling general procedure for aliphatic amines. Yellow oil (Yield: 90%). ^{1}H NMR (400 MHz, $CDCl_3$) δ (ppm) 3.20(q, J=4 Hz, 2H), 2.01(td, J=1.6-4 Hz, 2H), 1.98(t, J=4 Hz, 1Hz), 1.94-1.89(m, 2H), 1.87-1.82(m, 2H), 1.64(t, J=8 Hz, 2H), 1.52(s, J=4 Hz, 2H), 0.91(t, J=4 Hz, 3H); ^{13}C NMR (100 MHz, $CDCl_3$) δ (ppm) 171.26, 82.85, 69.31, 41.52, 32.54, 30.53, 28.55, 28.02, 22.94, 13.42, 11.48. HR-MS: Calculated for $[C_{11}H_{18}N_3O]^+$, 208.1450; found 208.1392.

Compound characterization

Compound 1

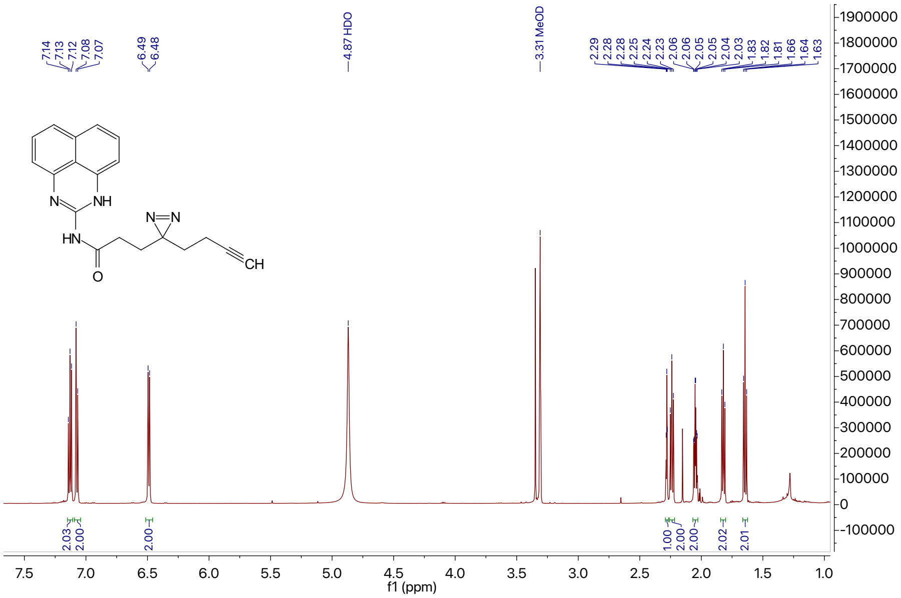

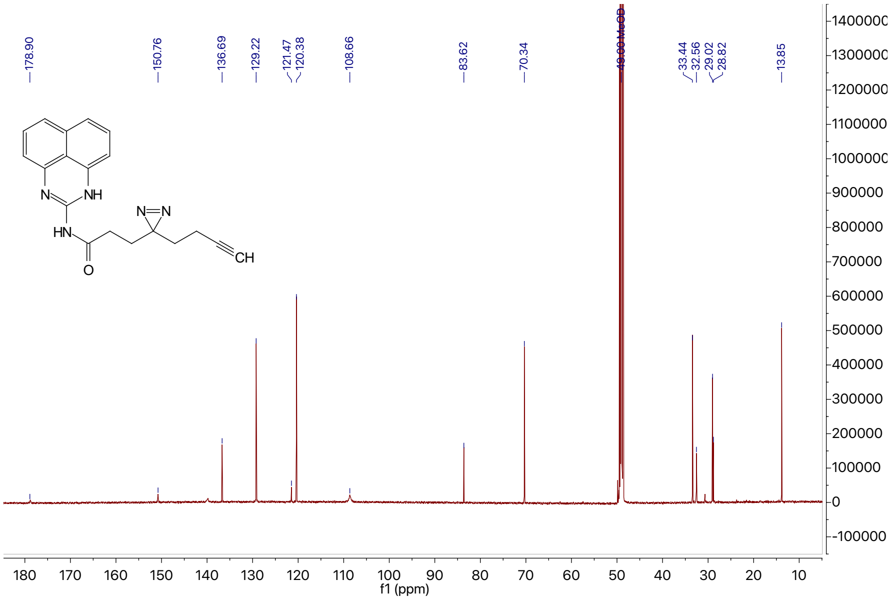

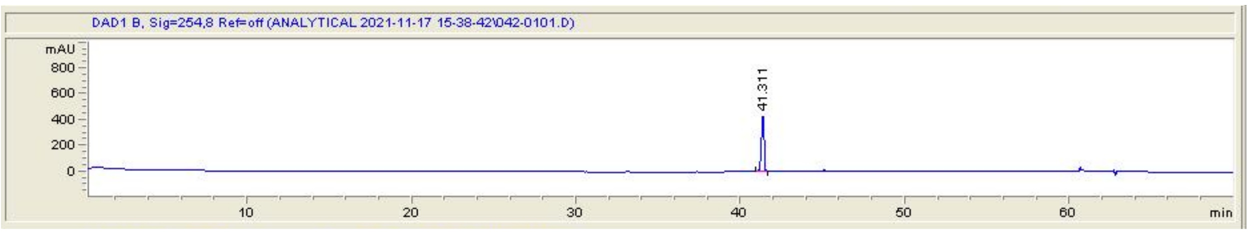

Compound 1a

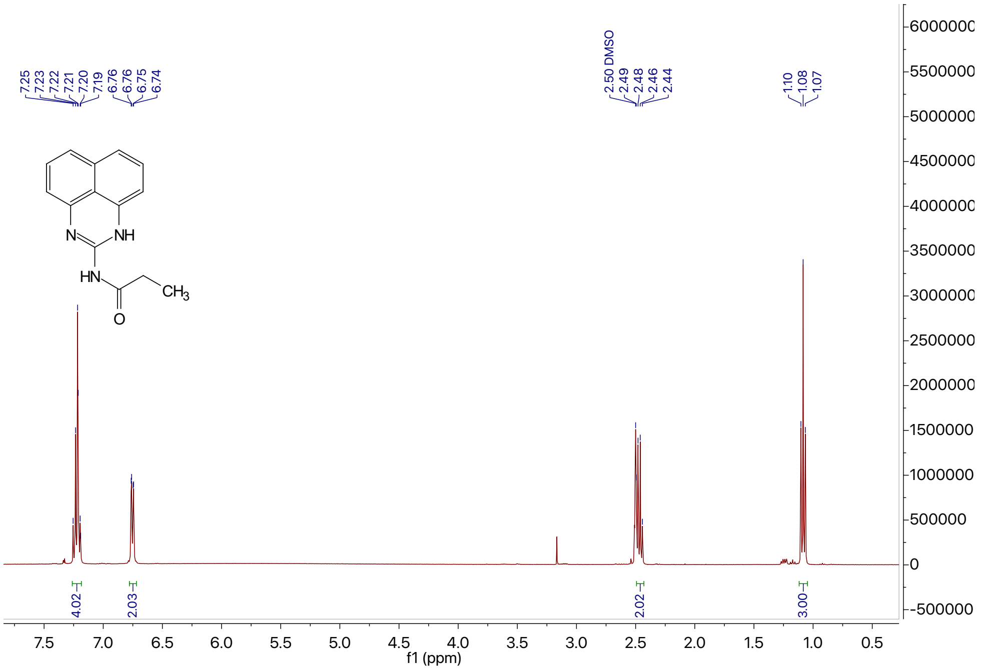
7.25
7.23
7.22
7.21
7.20
7.19
6.76
6.76
6.75
6.74
2.50 DMSO
2.49
2.48
2.46
2.44
1.10
1.08
1.07
N
NH
HN
CH3
O
4.02
2.03
2.02
3.00
f1 (ppm)

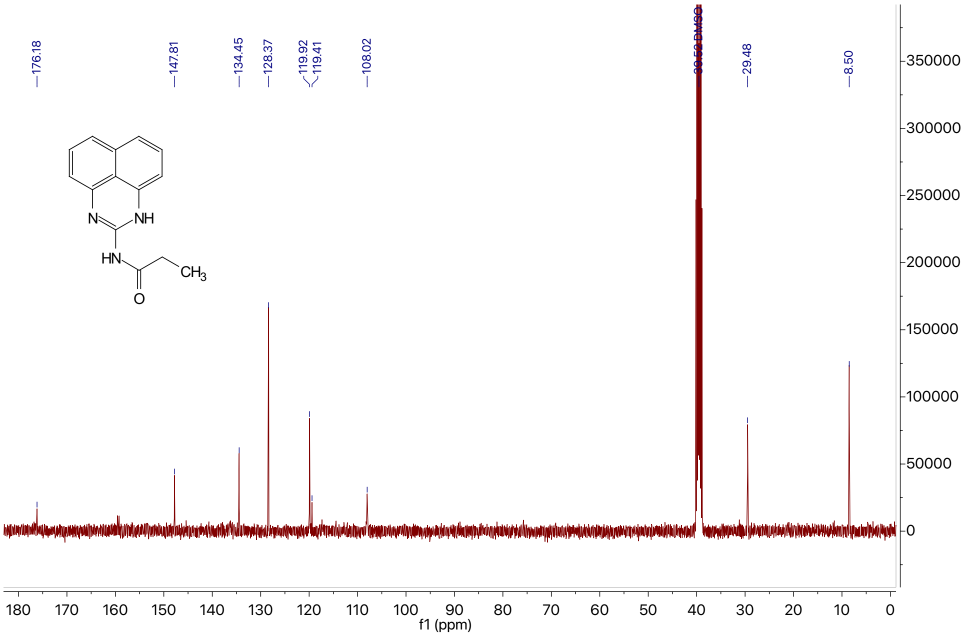
176.18
147.81
134.45
128.37
119.92
119.41
108.02
39.52 DMSO
29.48
8.50
N
NH
HN
CH3
O
f1 (ppm)

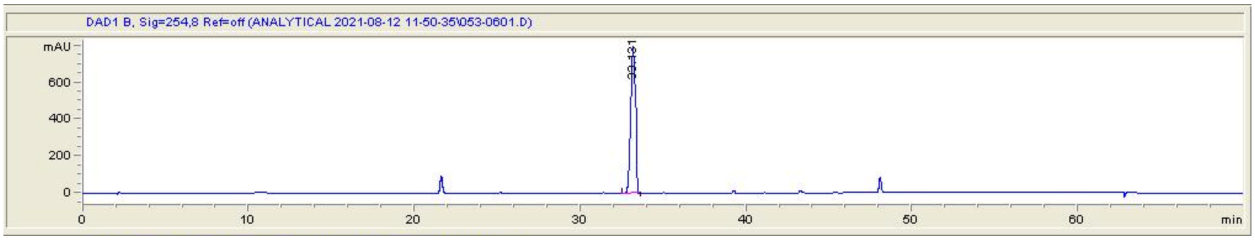
DAD1 B, Sig=254,8 Ref=off (ANALYTICAL 2021-08-12 11-50-35\053-0601.D)
mAU
33.131
min

Compound i1

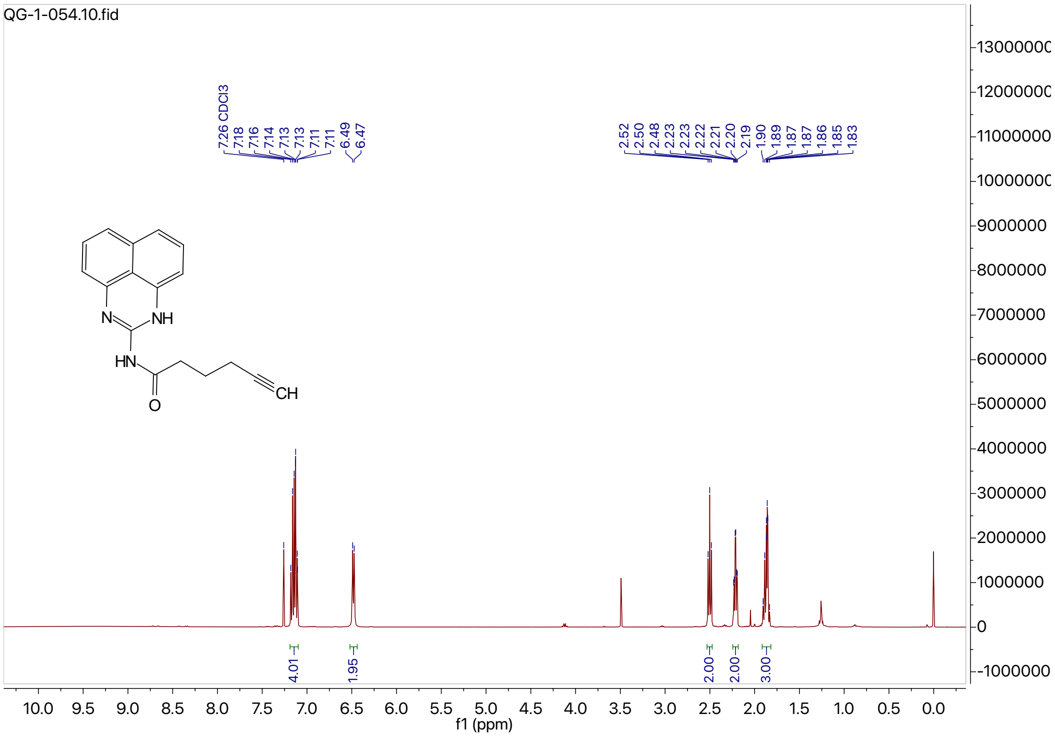
QG-1-054.10.fid
7.26 CDCl3
7.18
7.16
7.14
7.13
7.13
7.11
7.11
6.49
6.47
2.52
2.50
2.48
2.23
2.23
2.22
2.21
2.20
2.19
1.90
1.89
1.87
1.87
1.86
1.85
1.83
N
NH
HN
O
CH
4.01
1.95
2.00
2.00
3.00
f1 (ppm)

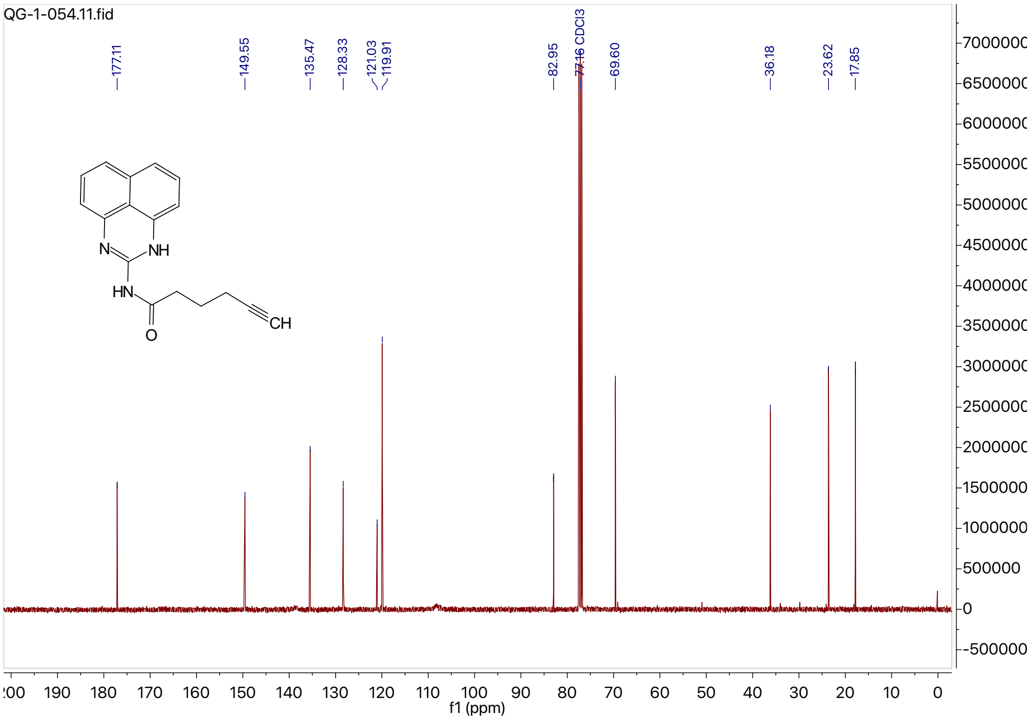
QG-1-054.11.fid
177.11
149.55
135.47
128.33
121.03
119.91
82.95
77.16 CDCl3
69.60
36.18
23.62
17.85
N
NH
HN
O
CH
f1 (ppm)

Compound i2

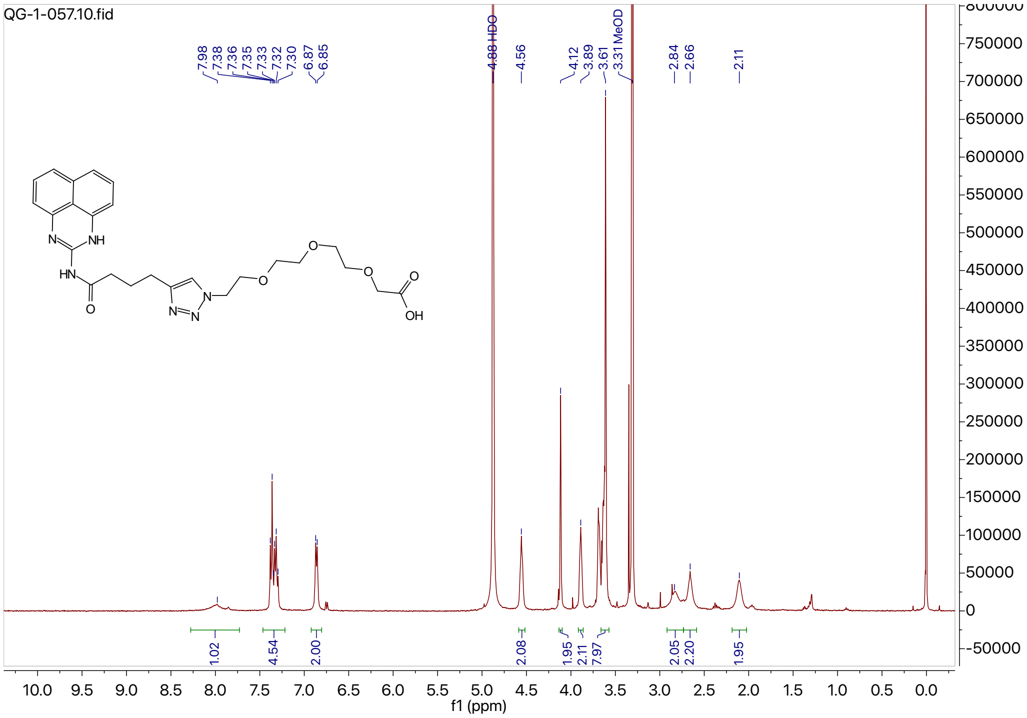
QG-1-057.10.fid
7.98
7.38
7.36
7.35
7.33
7.32
7.30
6.87
6.85
4.88 HDO
4.56
4.12
3.89
3.61
3.31 MeOD
2.84
2.66
2.11
1.02
4.54
2.00
2.08
1.95
2.11
7.97
2.05
2.20
1.95
f1 (ppm)

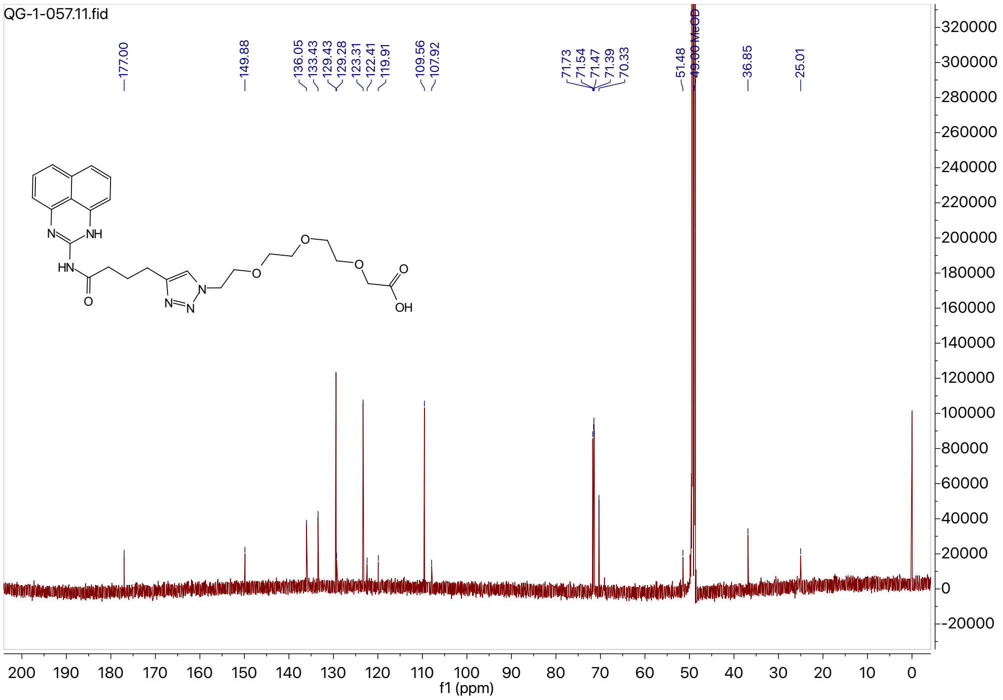
QG-1-057.11.fid
177.00
149.88
136.05
133.43
129.43
129.28
123.31
122.41
119.91
109.56
107.92
71.73
71.54
71.47
71.39
70.33
51.48
49.00 MeOD
36.85
25.01
f1 (ppm)

Compound 1b

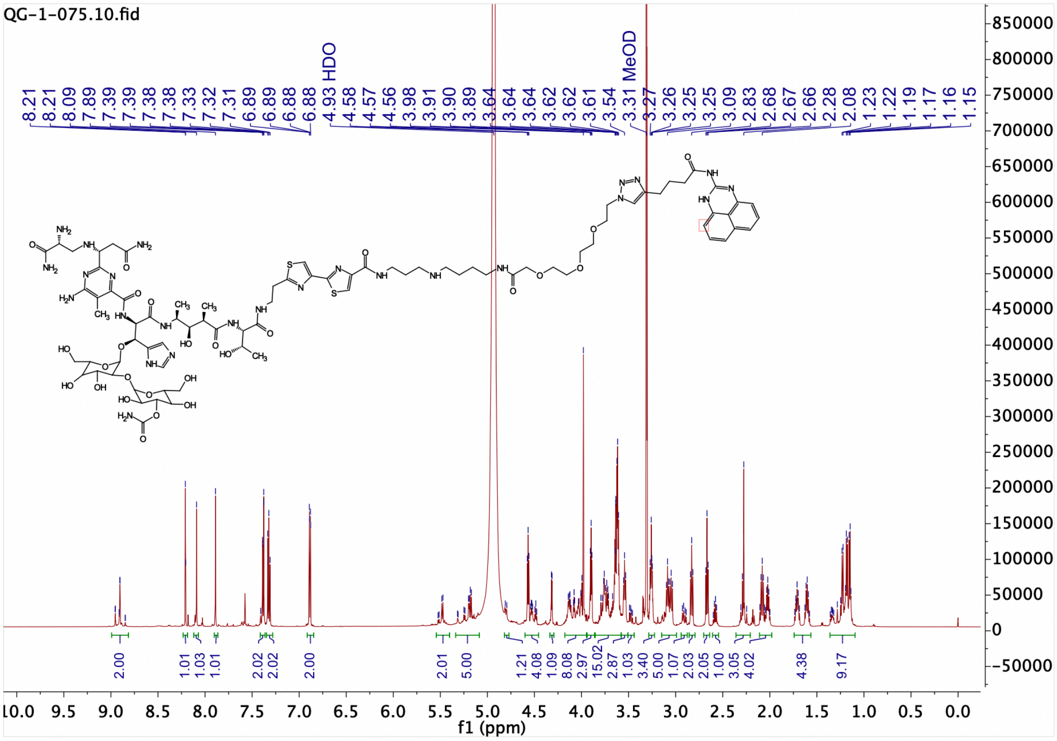

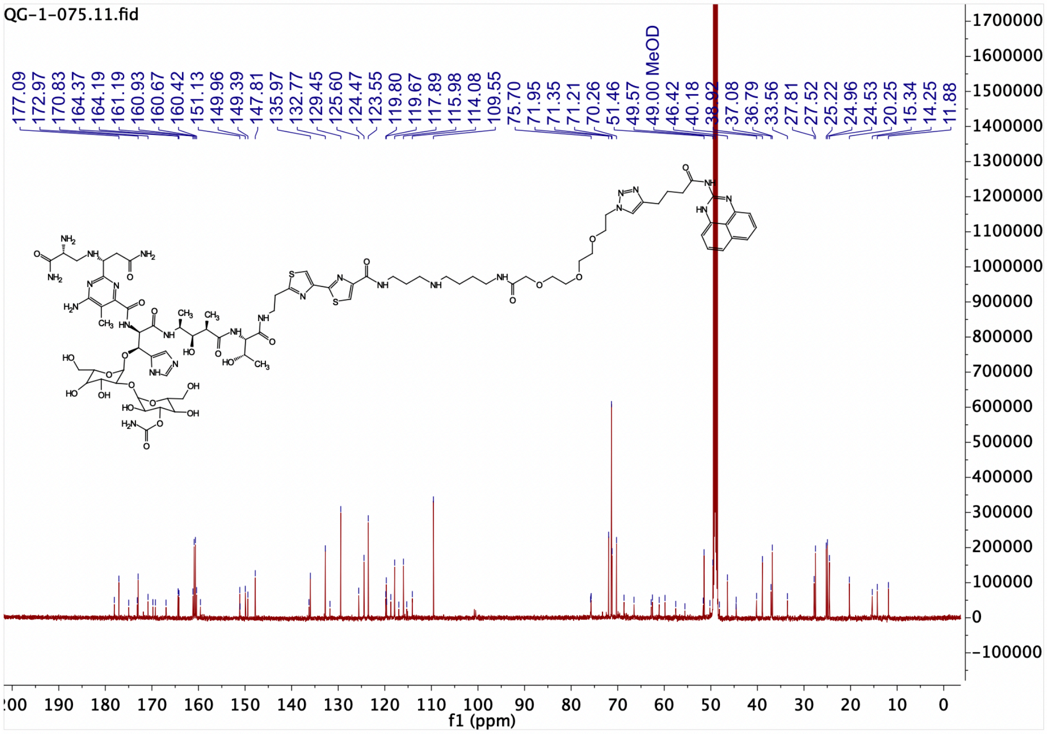

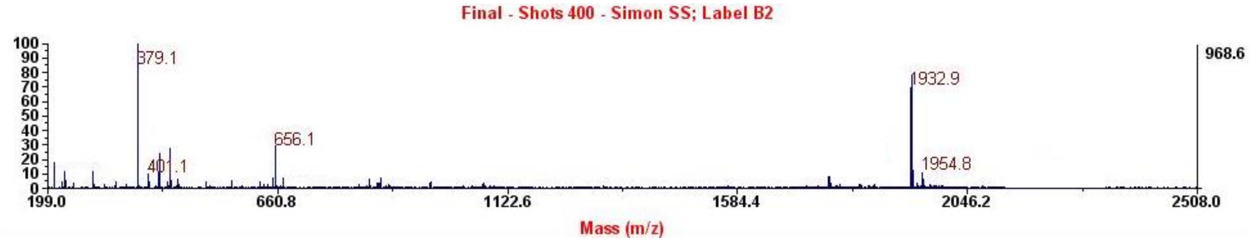
Final - Shots 400 - Simon SS; Label B2
379.1
401.1
656.1
1932.9
1954.8
968.6
Mass (m/z)

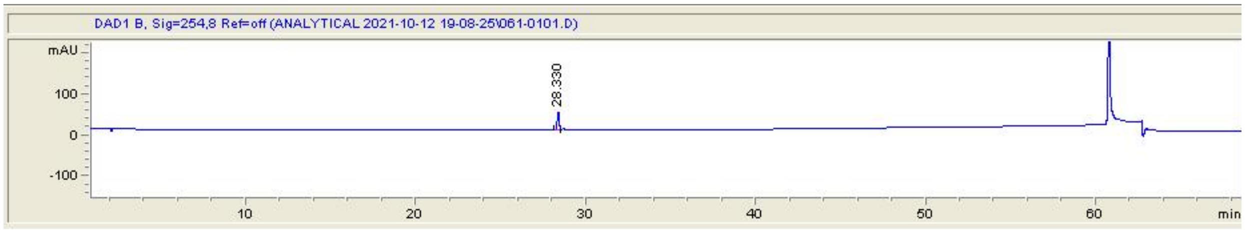
DAD1 B, Sig=254,8 Ref=off (ANALYTICAL 2021-10-12 19-08-25\061-0101.D)
mAU
28.330
min

Compound 2

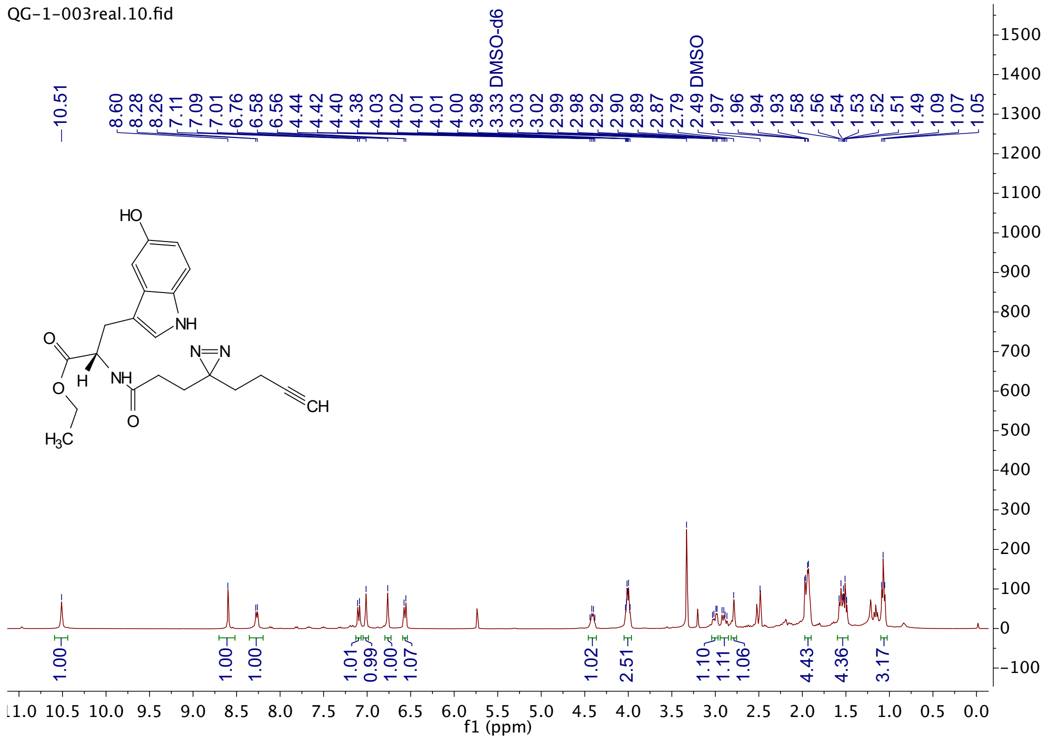
QG-1-003real.10.fid
10.51
8.60
8.28
8.26
7.11
7.09
7.01
6.76
6.58
6.56
4.44
4.42
4.40
4.38
4.03
4.02
4.01
4.01
4.00
3.98
3.33 DMSO-d6
3.03
3.02
2.99
2.98
2.92
2.90
2.89
2.87
2.79
2.49 DMSO
1.97
1.96
1.94
1.93
1.58
1.56
1.54
1.53
1.52
1.51
1.49
1.09
1.07
1.05
HO
NH
O
H
NH
N=N
O
O
CH
H_3C
1.00
1.00
1.00
1.01
0.99
1.00
1.07
1.02
2.51
1.10
1.11
1.06
4.43
4.36
3.17
f1 (ppm)

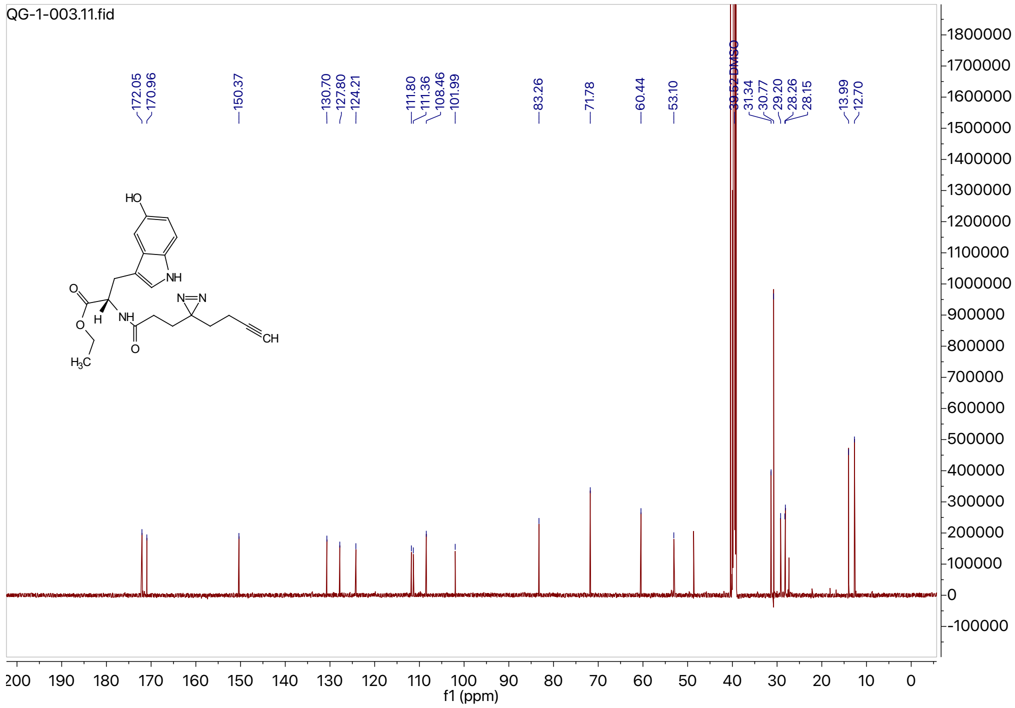
QG-1-003.11.fid
172.05
170.96
150.37
130.70
127.80
124.21
111.80
111.36
108.46
101.99
83.26
71.78
60.44
53.10
39.52 DMSO
31.34
30.77
29.20
28.26
28.15
13.99
12.70
HO
NH
O
H
NH
N=N
O
O
CH
H_3C
f1 (ppm)

Compound 3

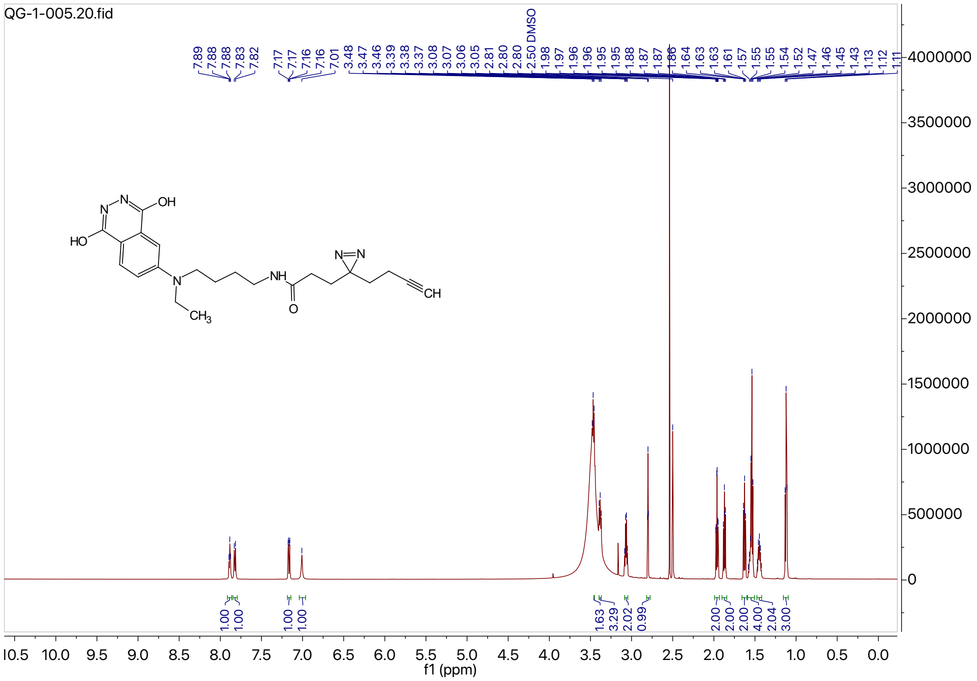

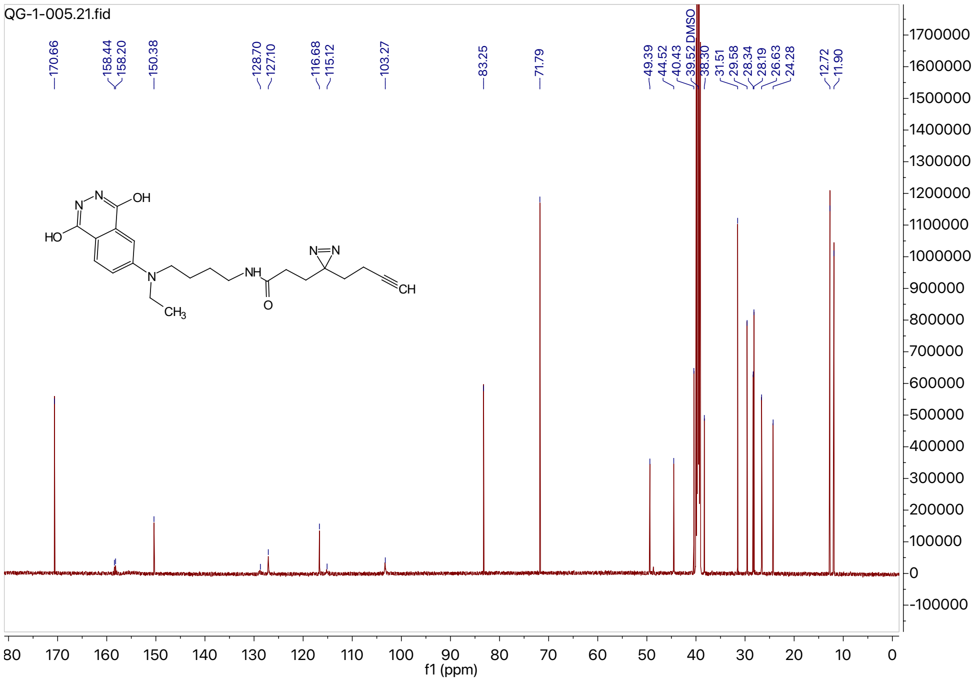

Compound 4

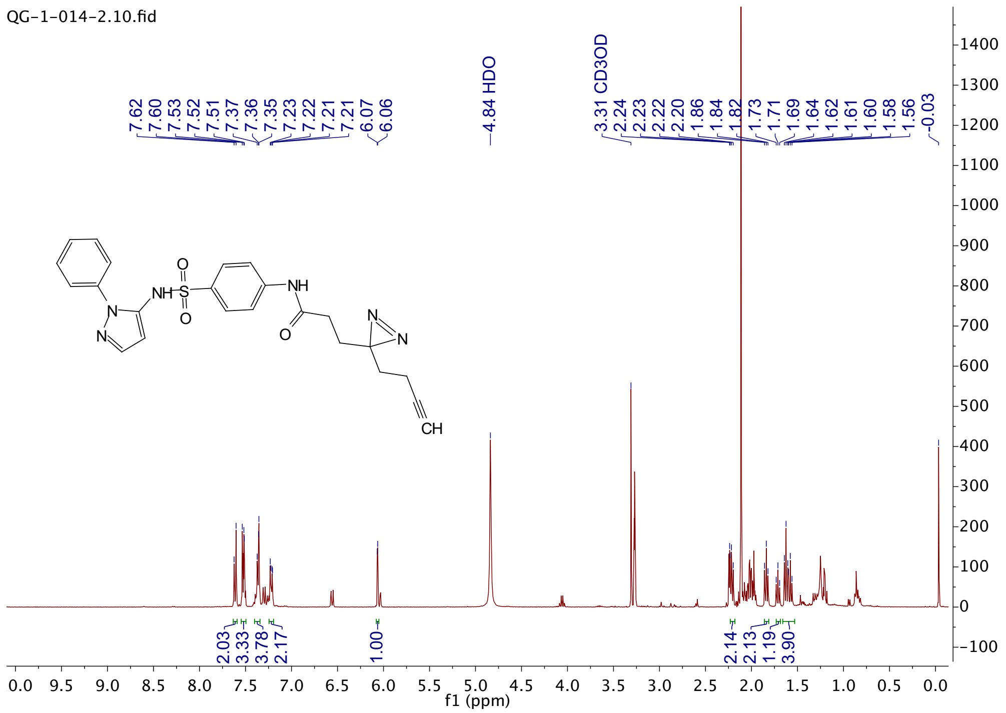

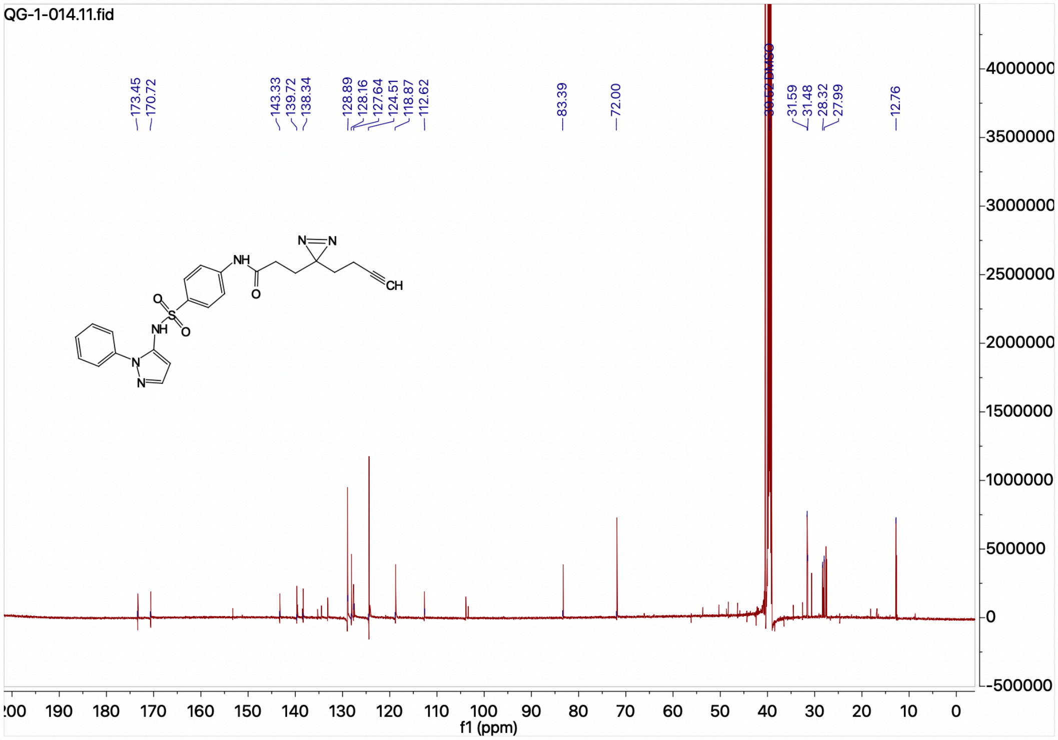

Compound 5

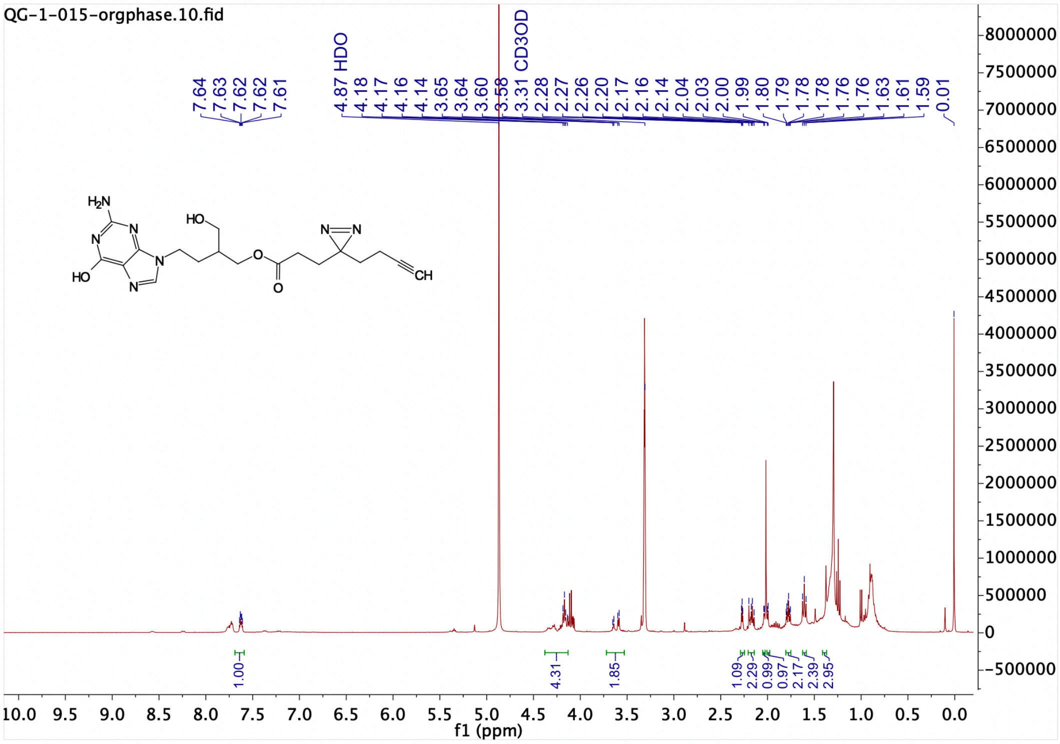

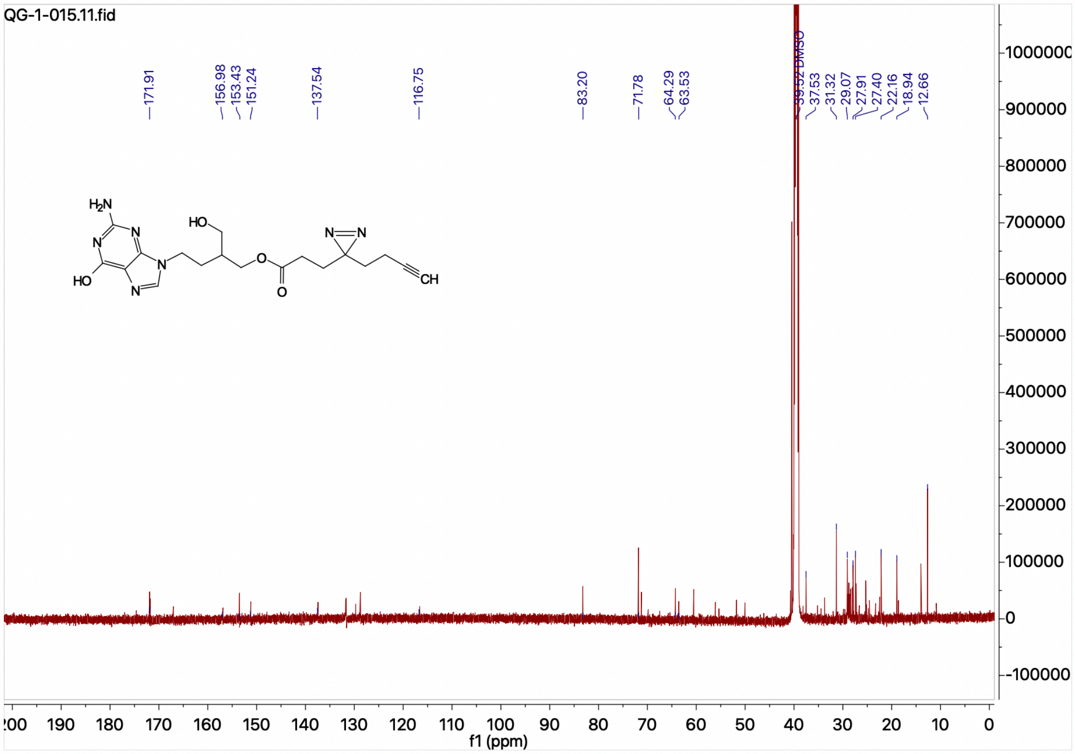

Compound 6

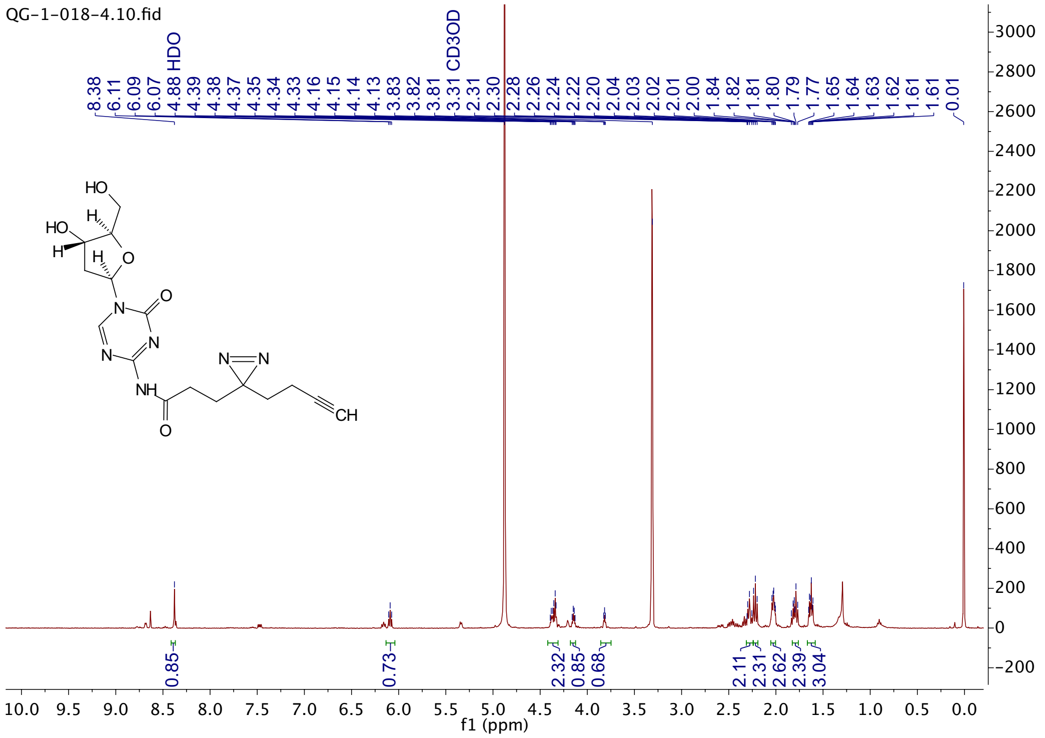
QG-1-018-4.10.fid
8.38
6.11
6.09
6.07
4.88 HDO
4.39
4.38
4.37
4.35
4.34
4.33
4.16
4.15
4.14
4.13
3.83
3.82
3.81
3.31 CD3OD
2.31
2.30
2.28
2.26
2.24
2.22
2.20
2.04
2.03
2.02
2.01
2.00
1.84
1.82
1.81
1.80
1.79
1.77
1.65
1.64
1.63
1.62
1.61
1.61
0.01
HO
HO
H
H
H
O
N
O
N
N
N
N
N
NH
O
CH
0.85
0.73
2.32
0.85
0.68
2.11
2.31
2.62
2.39
3.04
f1 (ppm)

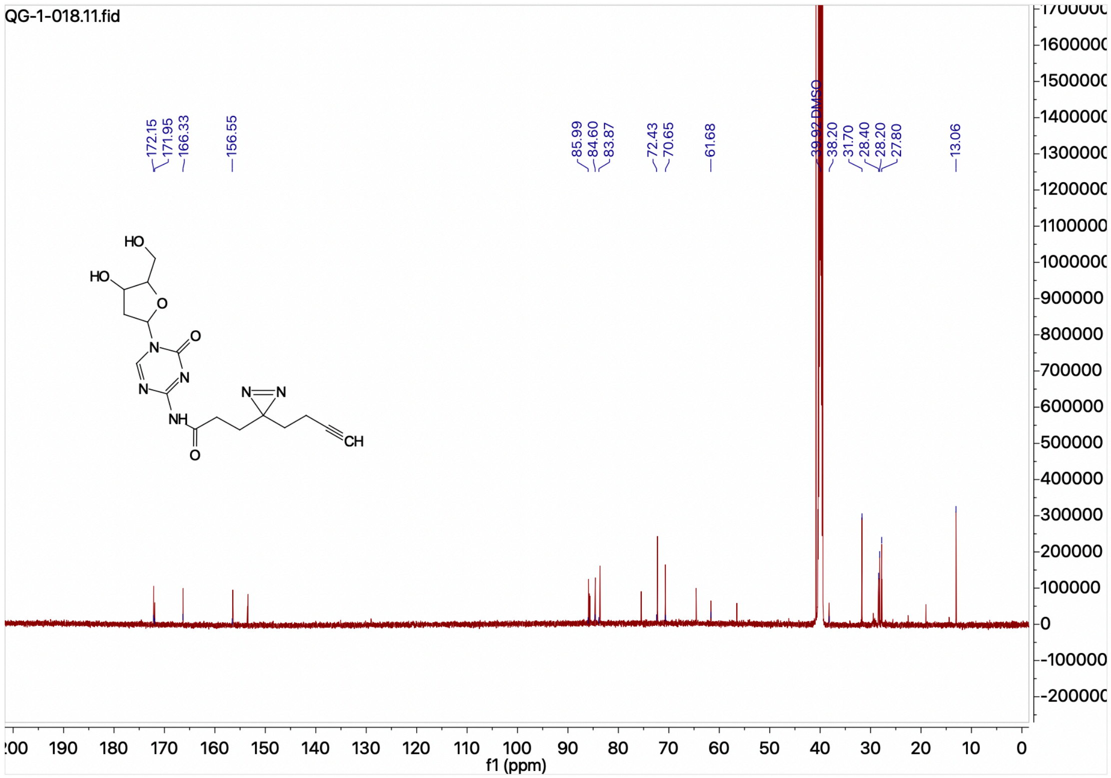
QG-1-018.11.fid
172.15
171.95
166.33
156.55
85.99
84.60
83.87
72.43
70.65
61.68
39.92 DMSO
38.20
31.70
28.40
28.20
27.80
13.06
HO
HO
O
N
O
N
N
N
N
NH
O
CH
f1 (ppm)

Compound 7

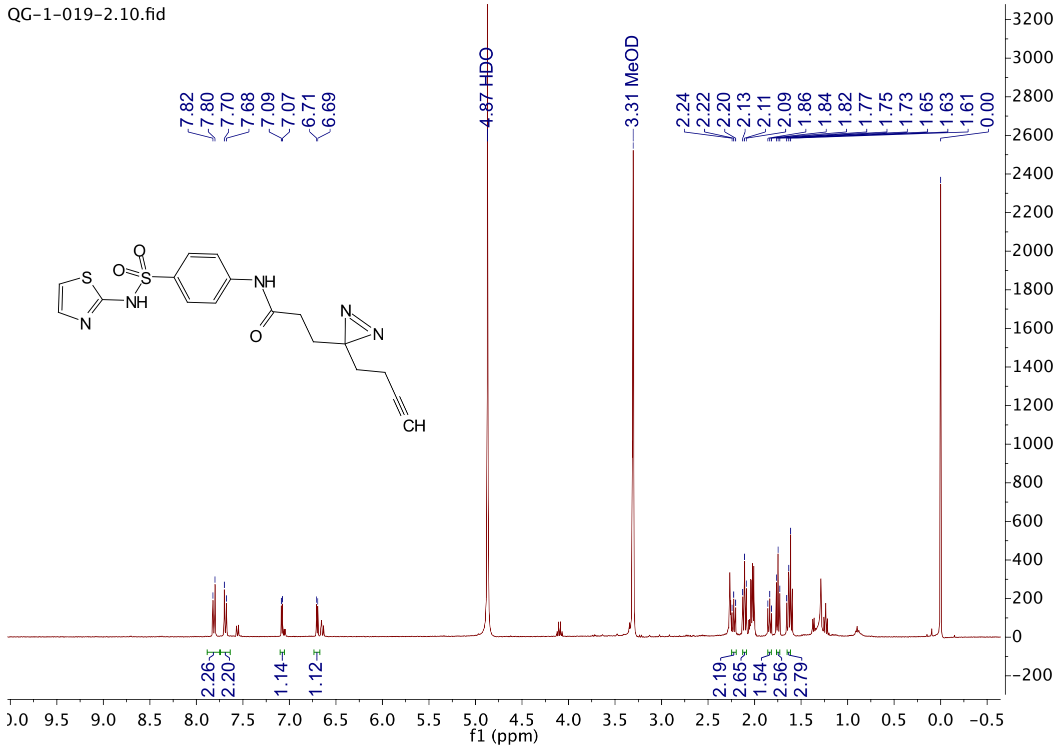
QG-1-019-2.10.fid
7.82
7.80
7.70
7.68
7.09
7.07
6.71
6.69
4.87 HDO
3.31 MeOD
2.24
2.22
2.20
2.13
2.11
2.09
1.86
1.84
1.82
1.77
1.75
1.73
1.65
1.63
1.61
0.00
2.26
2.20
1.14
1.12
2.19
2.65
1.54
2.56
2.79
f1 (ppm)

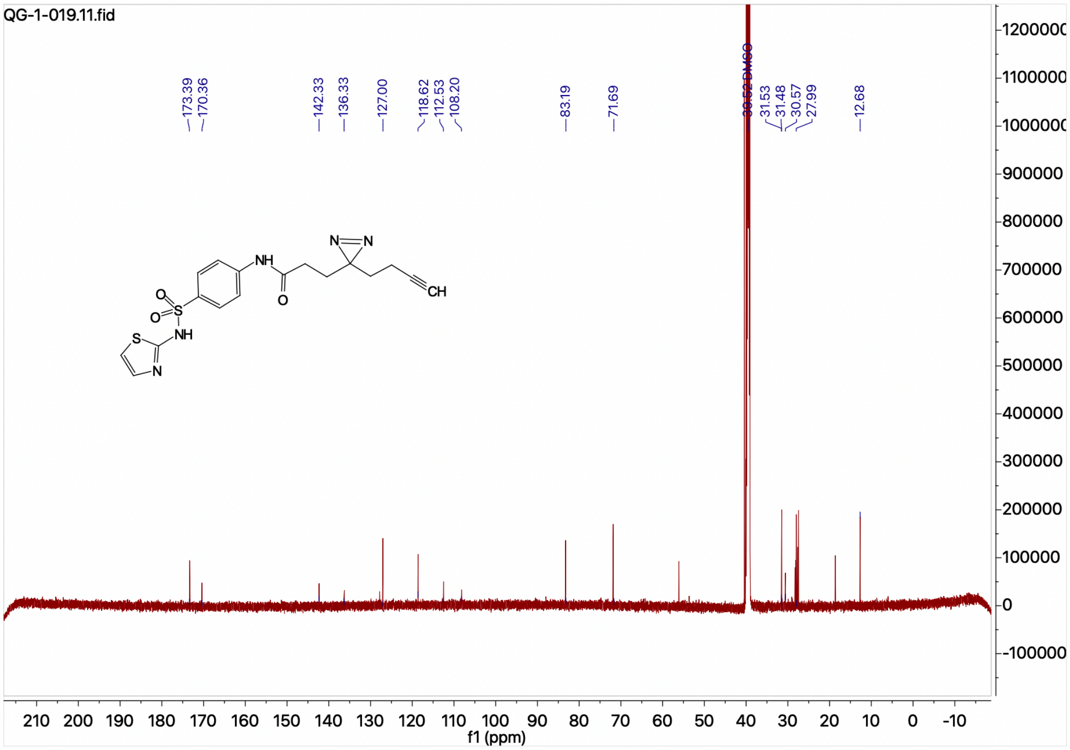
QG-1-019.11.fid
173.39
170.36
142.33
136.33
127.00
118.62
112.53
108.20
83.19
71.69
31.53
31.48
30.57
27.99
12.68
f1 (ppm)

Compound 8

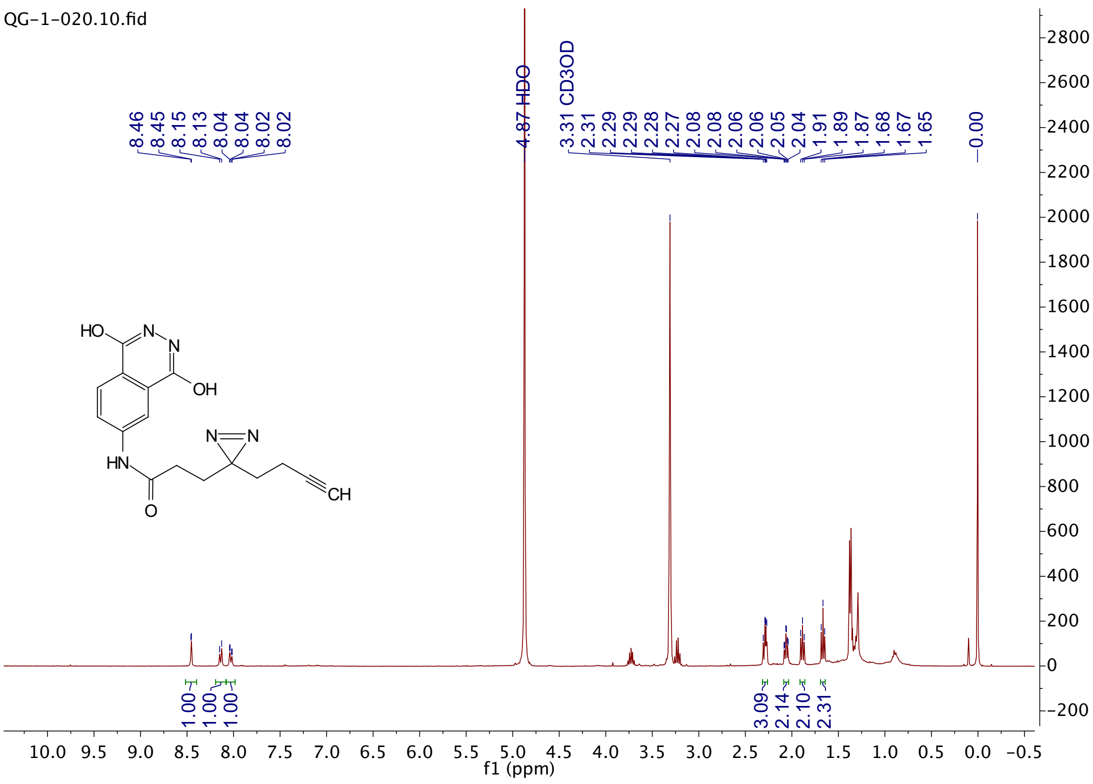
QG-1-020.10.fid
8.46
8.45
8.15
8.13
8.04
8.04
8.02
8.02
4.87 HDO
3.31 CD3OD
2.31
2.29
2.29
2.28
2.27
2.08
2.08
2.06
2.06
2.05
2.04
1.91
1.89
1.87
1.68
1.67
1.65
0.00
HO
N
N
OH
N
N
HN
O
CH
1.00
1.00
1.00
3.09
2.14
2.10
2.31
f1 (ppm)

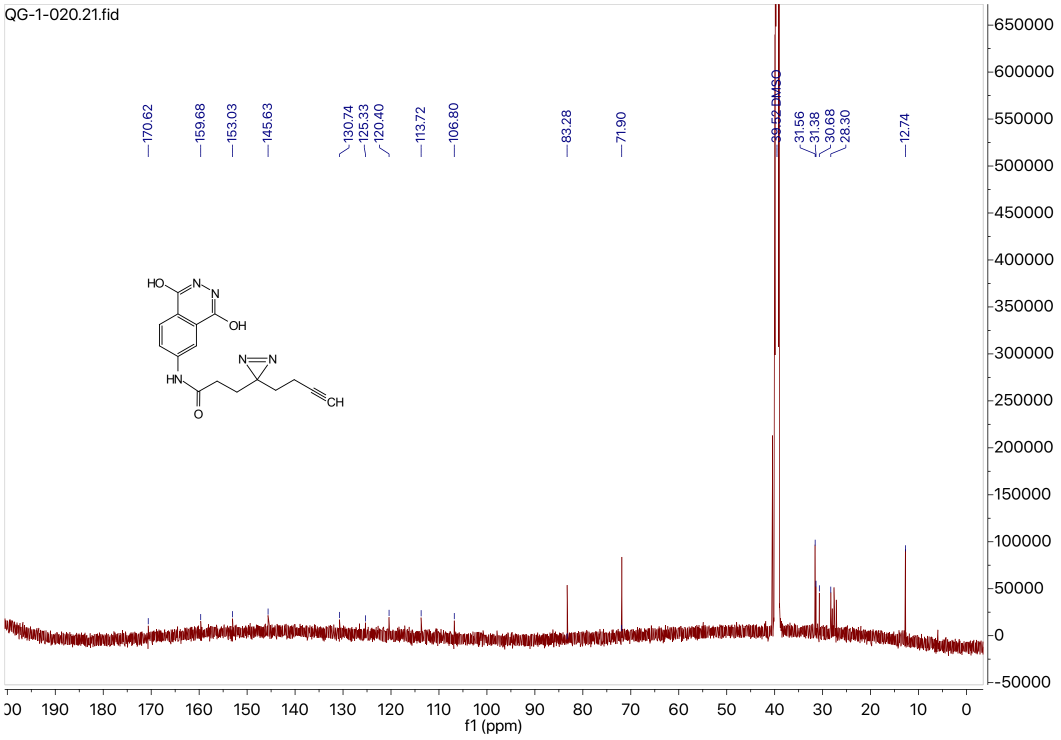
QG-1-020.21.fid
170.62
159.68
153.03
145.63
130.74
125.33
120.40
113.72
106.80
83.28
71.90
39.52 DMSO
31.56
31.38
30.68
28.30
12.74
HO
N
N
OH
N
N
HN
O
CH
f1 (ppm)

Compound 9

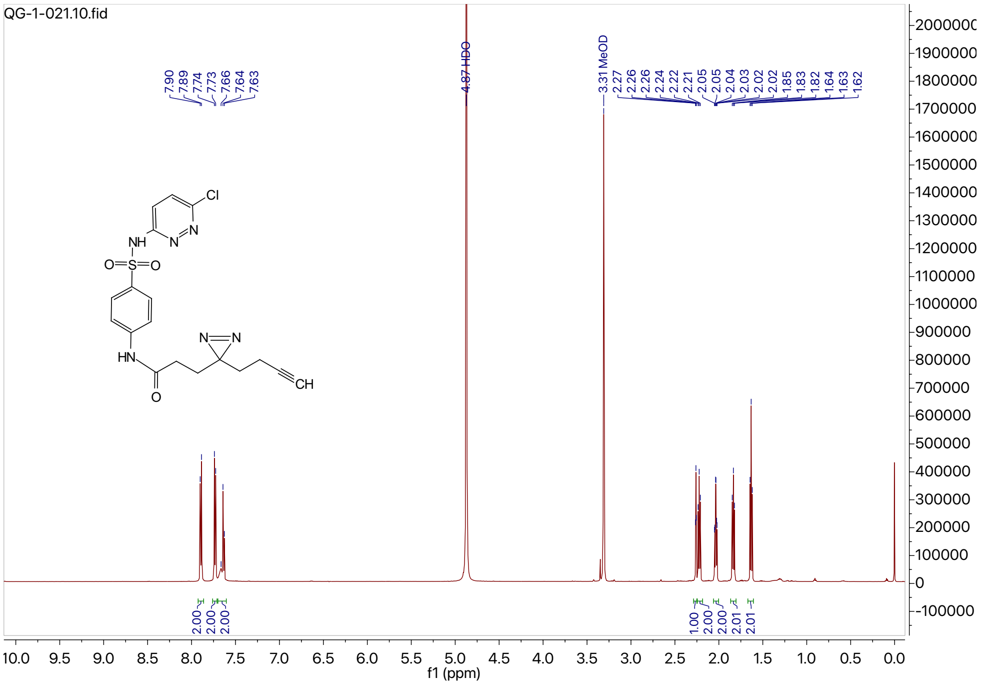
QG-1-021.10.fid
7.90
7.89
7.74
7.73
7.66
7.64
7.63
4.87 HDO
3.31 MeOD
2.27
2.26
2.26
2.24
2.22
2.21
2.05
2.05
2.04
2.03
2.02
2.02
1.85
1.83
1.82
1.64
1.63
1.62
Cl
N
N
NH
O=S=O
HN
N=N
CH
O
2.00
2.00
2.00
1.00
2.00
2.00
2.01
2.01
f1 (ppm)

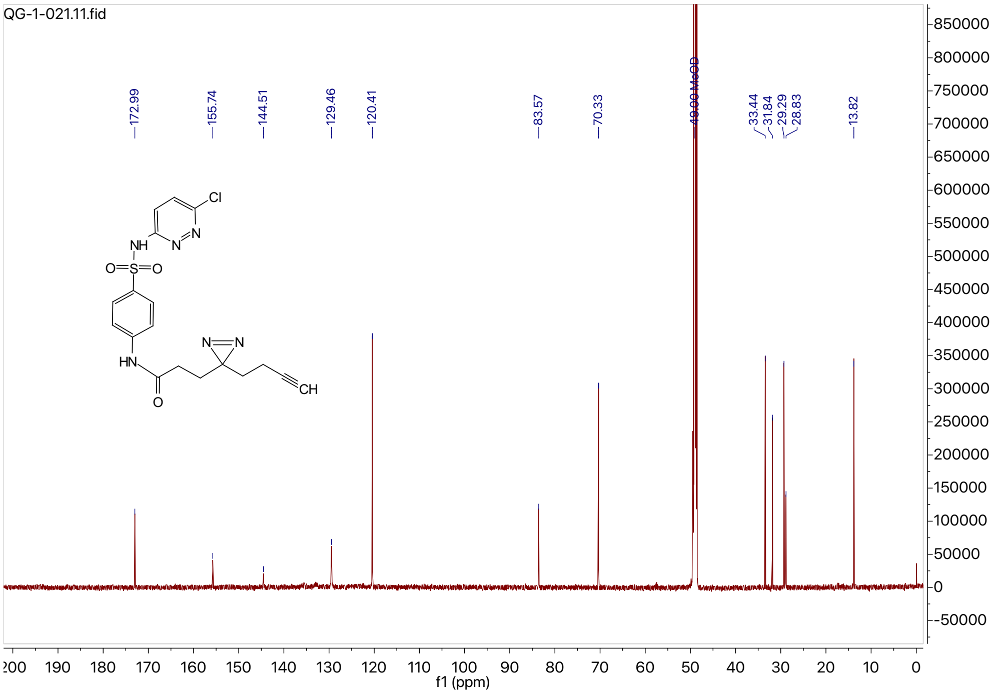
QG-1-021.11.fid
172.99
155.74
144.51
129.46
120.41
83.57
70.33
49.00 MeOD
33.44
31.84
29.29
28.83
13.82
Cl
N
N
NH
O=S=O
HN
N=N
CH
O
f1 (ppm)

Compound 10

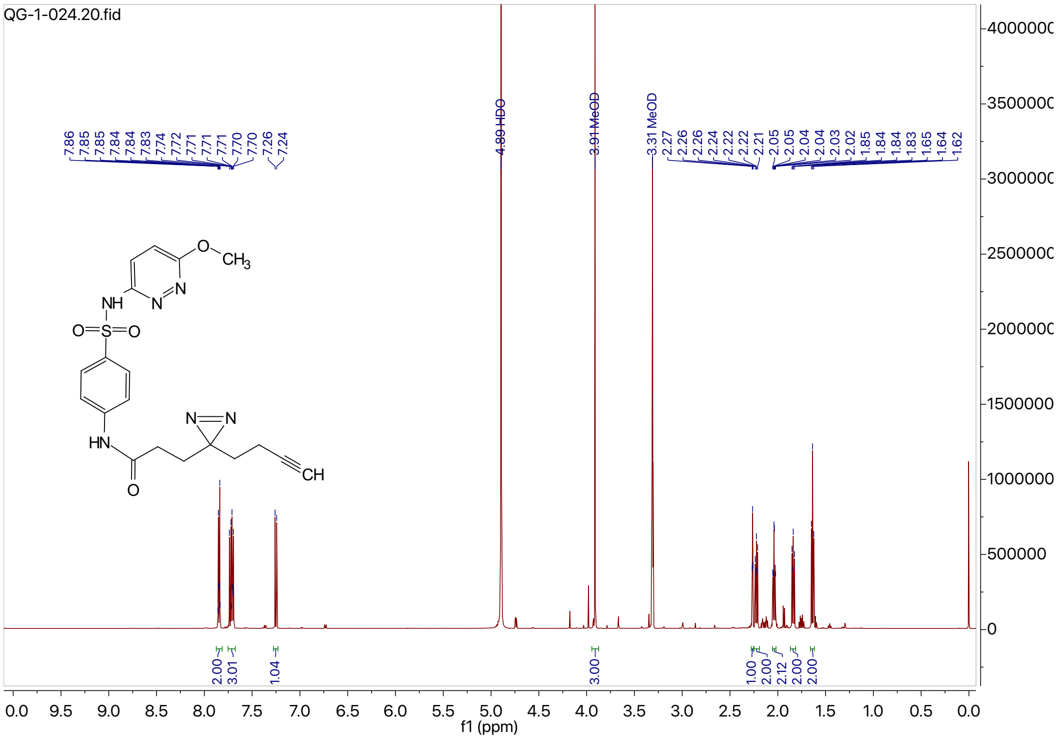

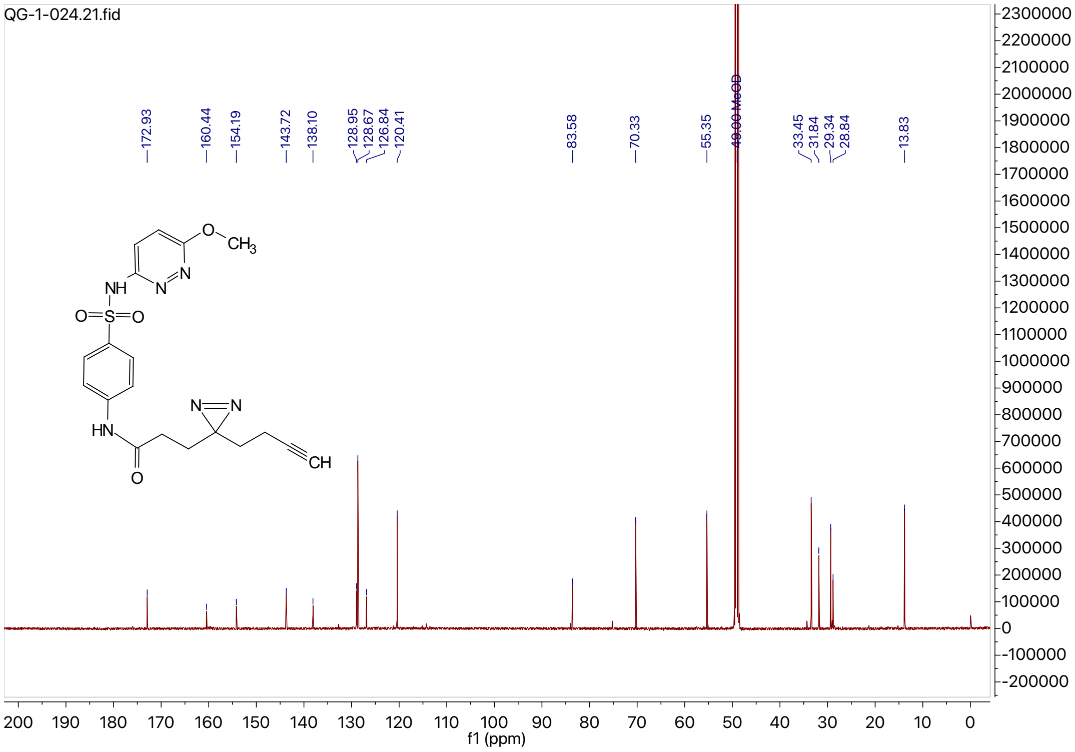

Compound 11

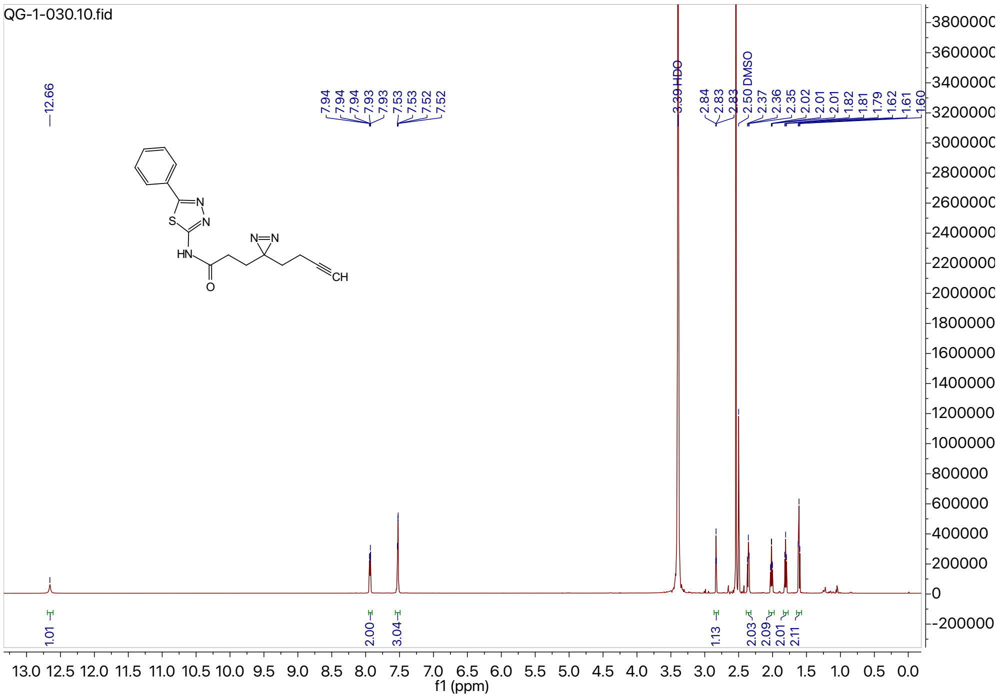
QG-1-030.10.fid
12.66
7.94
7.94
7.94
7.93
7.93
7.53
7.53
7.52
7.52
3.39 HDO
2.84
2.83
2.83
2.50 DMSO
2.37
2.36
2.35
2.02
2.01
2.01
1.82
1.81
1.79
1.62
1.61
1.60
HN
S
N
N
N=N
O
CH
1.01
2.00
3.04
1.13
2.03
2.09
2.01
2.11
f1 (ppm)

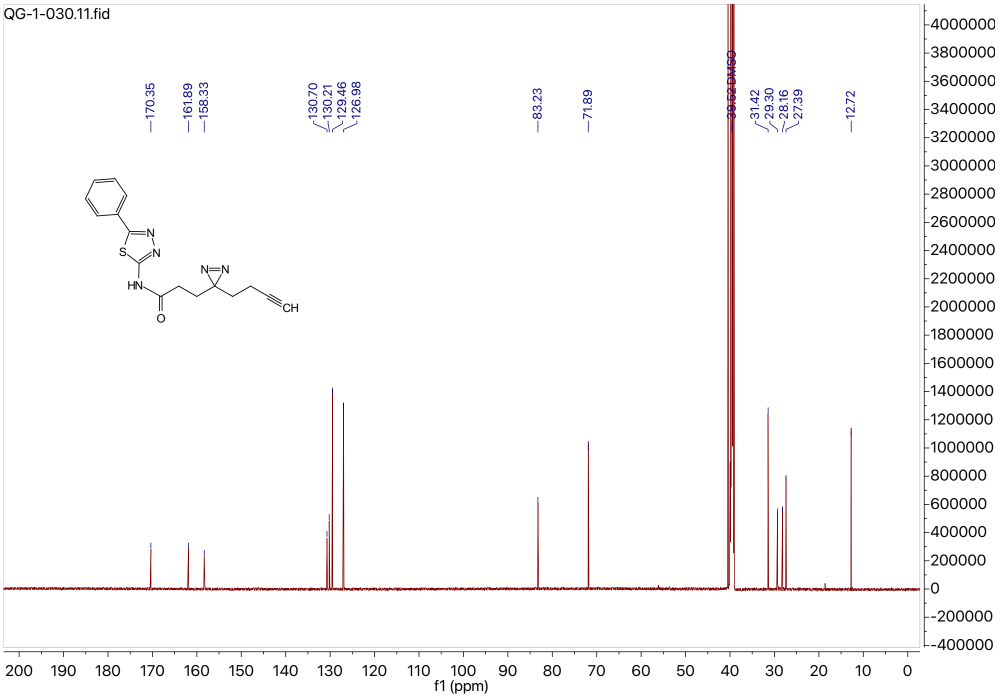
QG-1-030.11.fid
170.35
161.89
158.33
130.70
130.21
129.46
126.98
83.23
71.89
39.52 DMSO
31.42
29.30
28.16
27.39
12.72
HN
S
N
N
N=N
O
CH
f1 (ppm)

Compound 12

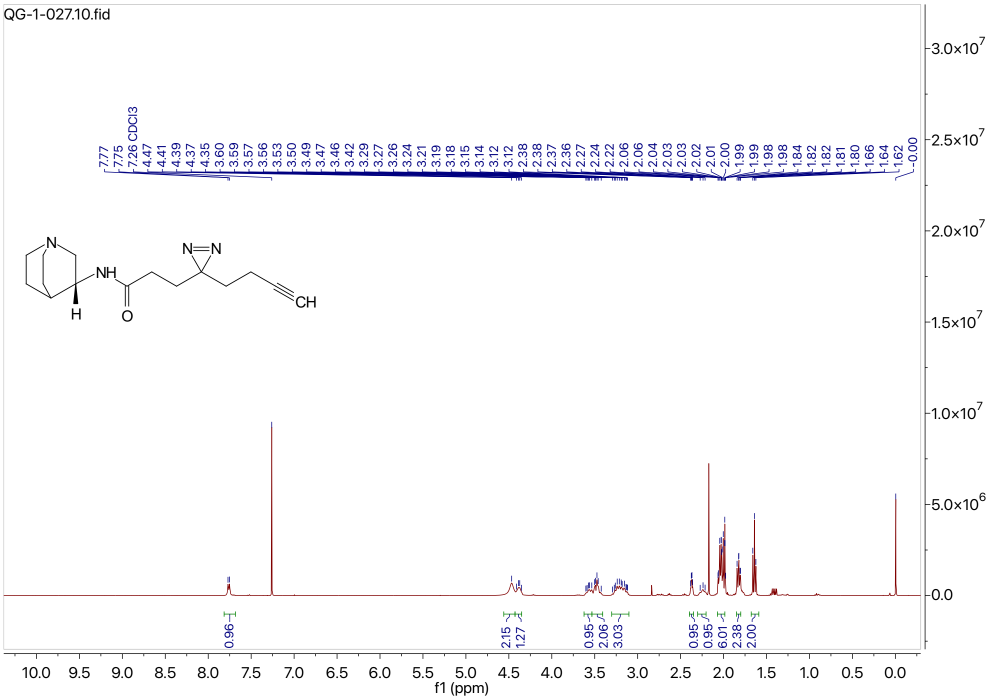
QG-1-027.10.fid
7.77
7.75
7.26 CDCl3
4.47
4.41
4.39
4.37
4.35
3.60
3.59
3.57
3.56
3.53
3.50
3.49
3.47
3.46
3.42
3.29
3.27
3.26
3.24
3.21
3.19
3.18
3.15
3.14
3.12
3.12
2.38
2.38
2.37
2.36
2.27
2.24
2.22
2.06
2.06
2.04
2.03
2.03
2.02
2.01
2.00
1.99
1.99
1.98
1.98
1.84
1.82
1.82
1.81
1.80
1.66
1.64
1.62
-0.00
0.96
2.15
1.27
0.95
2.06
3.03
0.95
0.95
6.01
2.38
2.00
f1 (ppm)

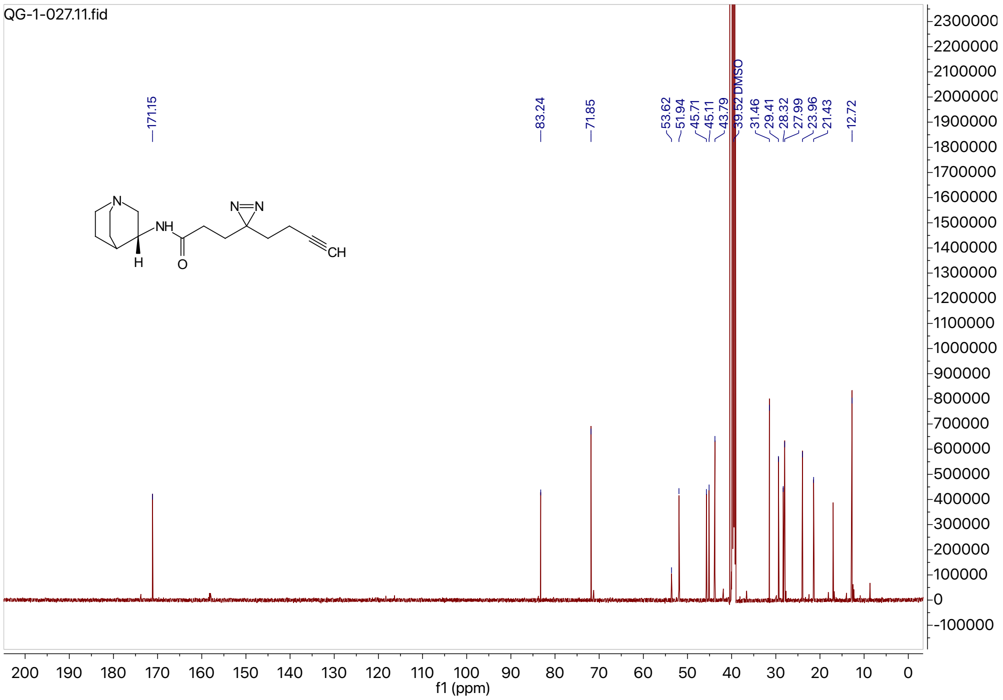
QG-1-027.11.fid
171.15
83.24
71.85
53.62
51.94
45.71
45.11
43.79
39.52 DMSO
31.46
29.41
28.32
27.99
23.96
21.43
12.72
f1 (ppm)

Compound 13

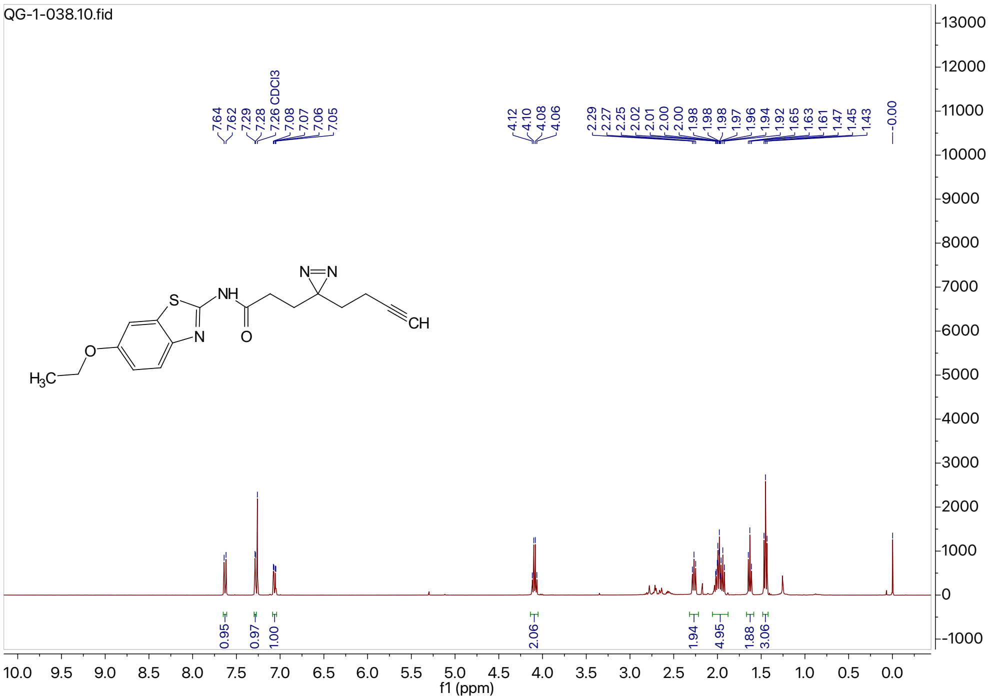
QG-1-038.10.fid
7.64
7.62
7.29
7.28
7.26 CDCl3
7.08
7.07
7.06
7.05
4.12
4.10
4.08
4.06
2.29
2.27
2.25
2.02
2.01
2.00
2.00
1.98
1.98
1.98
1.97
1.96
1.94
1.92
1.65
1.63
1.61
1.47
1.45
1.43
0.00
0.95
0.97
1.00
2.06
1.94
4.95
1.88
3.06
f1 (ppm)

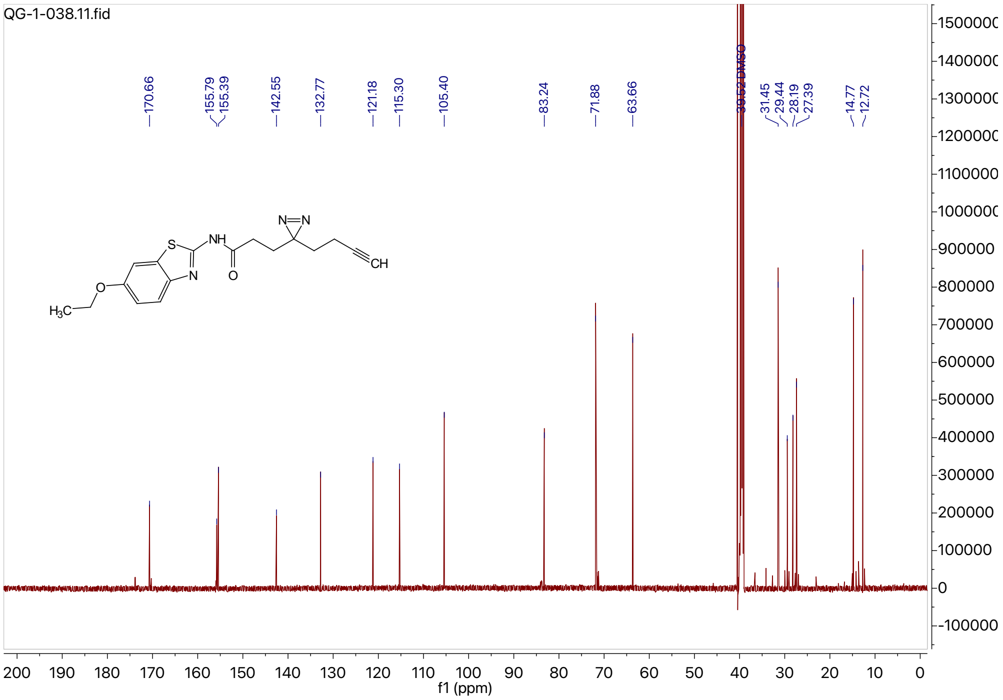
QG-1-038.11.fid
170.66
155.79
155.39
142.55
132.77
121.18
115.30
105.40
83.24
71.88
63.66
39.52 DMSO
31.45
29.44
28.19
27.39
14.77
12.72
f1 (ppm)

Compound 14

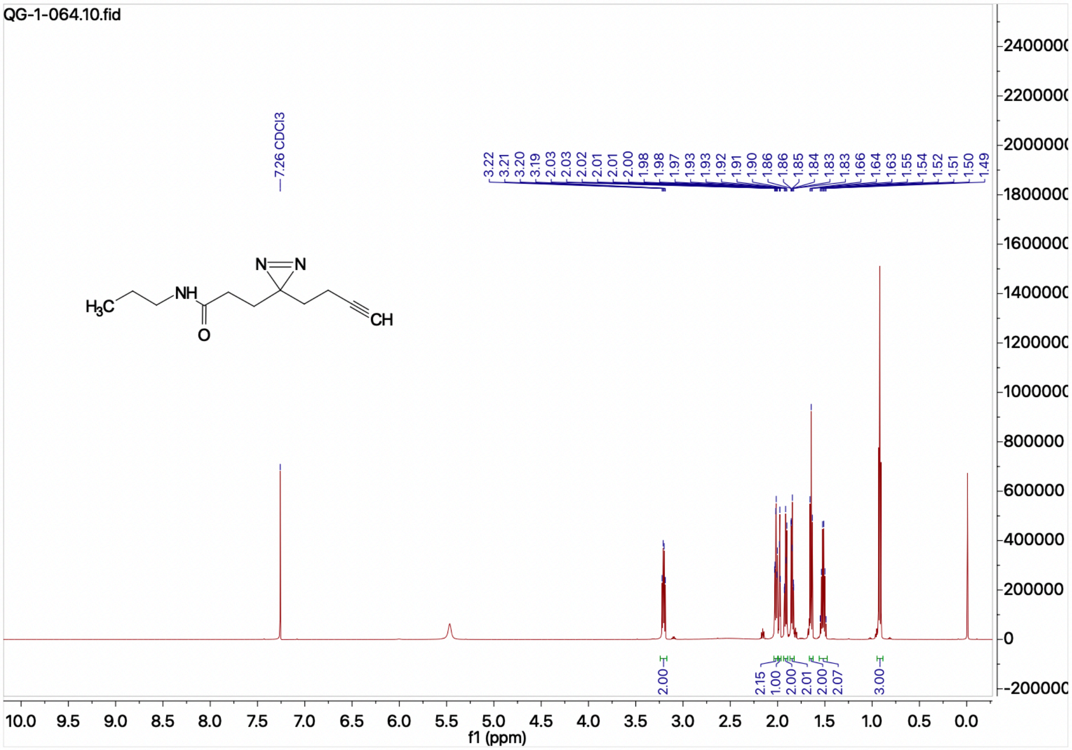

QG-1-064.10.fid
7.26 CDCl3
3.22
3.21
3.20
3.19
2.03
2.03
2.02
2.01
2.01
2.00
1.98
1.98
1.97
1.93
1.93
1.92
1.91
1.90
1.86
1.86
1.85
1.84
1.83
1.83
1.66
1.64
1.63
1.55
1.54
1.52
1.51
1.50
1.49
2.00
2.15
1.00
2.00
2.01
2.00
2.07
3.00
f1 (ppm)

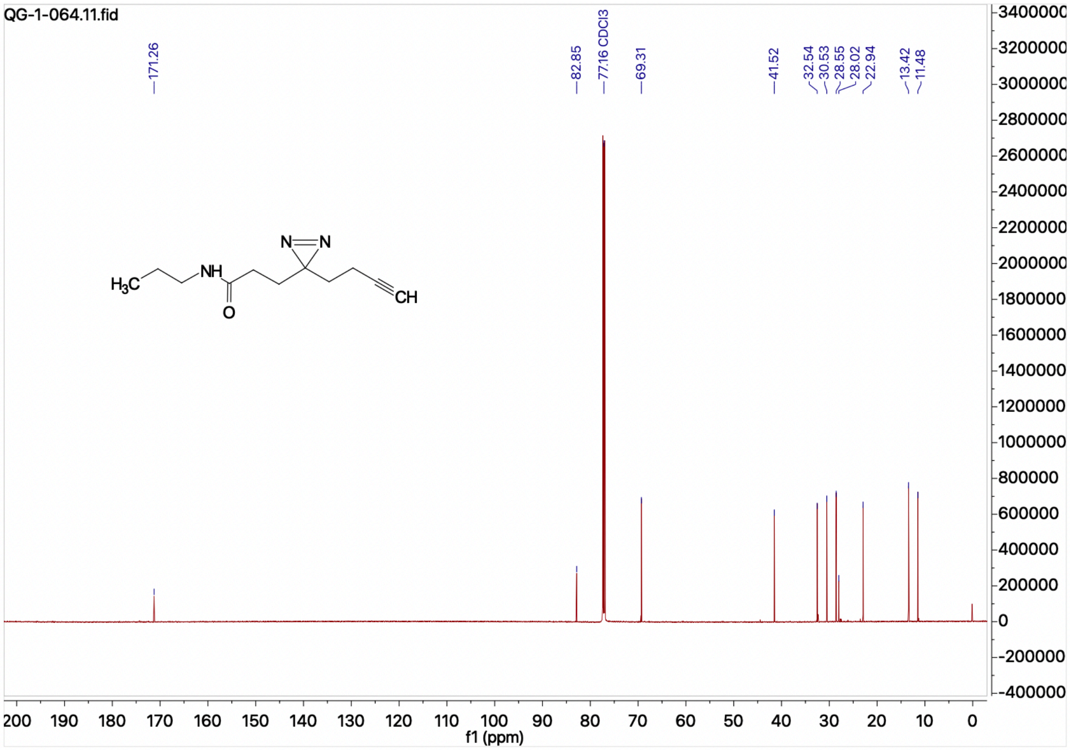

QG-1-064.11.fid
171.26
82.85
77.16 CDCl3
69.31
41.52
32.54
30.53
28.55
28.02
22.94
13.42
11.48
f1 (ppm)

References

1. Tong, Y.; Gibaut, Q. M. R.; Rouse, W.; Childs-Disney, J. L.; Suresh, B. M.; Abegg, D.; Choudhary, S.; Akahori, Y.; Adibekian, A.; Moss, W. N.; Disney, M. D., Transcriptome-wide mapping of small-molecule RNA-binding sites in cells informs an isoform-specific degrader of QSOX1 mRNA. *J. Am. Chem. Soc.* **2022,** *144* (26), 11620–11625.

2. Angelbello, A. J.; Rzuczek, S. G.; McKee, K. K.; Chen, J. L.; Olafson, H.; Cameron, M. D.; Moss, W. N.; Wang, E. T.; Disney, M. D., Precise small-molecule cleavage of an r(CUG) repeat expansion in a myotonic dystrophy mouse model. *Proc. Natl. Acad. Sci. U. S. A.* **2019,** *116* (16), 7799-7804.

3. Reddy, K.; Jenquin, J. R.; McConnell, O. L.; Cleary, J. D.; Richardson, J. I.; Pinto, B. S.; Haerle, M. C.; Delgado, E.; Planco, L.; Nakamori, M.; Wang, E. T.; Berglund, J. A., A CTG repeat-selective chemical screen identifies microtubule inhibitors as selective modulators of toxic CUG RNA levels. *Proc. Natl. Acad. Sci. U. S. A.* **2019,** *116* (42), 20991-21000.

4. Suresh, B. M.; Li, W.; Zhang, P.; Wang, K. W.; Yildirim, I.; Parker, C. G.; Disney, M. D., A general fragment-based approach to identify and optimize bioactive ligands targeting RNA. *Proc. Natl. Acad. Sci. U. S. A.* **2020,** *117* (52), 33197-33203.

5. Angelbello, A. J.; DeFeo, M. E.; Glinkerman, C. M.; Boger, D. L.; Disney, M. D., Precise targeted cleavage of a r(CUG) repeat expansion in cells by using a small-molecule-deglycobleomycin conjugate. *ACS Chem. Biol.* **2020,** *15* (4), 849-855.

6. Li, Y.; Disney, M. D., Precise small molecule degradation of a noncoding RNA identifies cellular binding sites and modulates an oncogenic phenotype. *ACS Chem. Biol.* **2018,** *13* (11), 3065-3071.

7. Chen, J. L.; Zhang, P.; Abe, M.; Aikawa, H.; Zhang, L.; Frank, A. J.; Zembryski, T.; Hubbs, C.; Park, H.; Withka, J.; Steppan, C.; Rogers, L.; Cabral, S.; Pettersson, M.; Wager, T. T.; Fountain, M. A.; Rumbaugh, G.; Childs-Disney, J. L.; Disney, M. D., Design, optimization, and study of small molecules that target Tau pre-mRNA and affect splicing. *J. Am. Chem. Soc.* **2020,** *142* (19), 8706-8727.

8. Arandel, L.; Polay Espinoza, M.; Matloka, M.; Bazinet, A.; De Dea Diniz, D.; Naouar, N.; Rau, F.; Jollet, A.; Edom-Vovard, F.; Mamchaoui, K.; Tarnopolsky, M.;

Puymirat, J.; Battail, C.; Boland, A.; Deleuze, J. F.; Mouly, V.; Klein, A. F.; Furling, D., Immortalized human myotonic dystrophy muscle cell lines to assess therapeutic compounds. *Dis. Model. Mech.* **2017,** *10* (4), 487-497.

9. Schmittgen, T. D.; Livak, K. J., Analyzing real-time PCR data by the comparative C(T) method. *Nat. Protoc.* **2008,** *3* (6), 1101-1108.

10. Dobin, A.; Davis, C. A.; Schlesinger, F.; Drenkow, J.; Zaleski, C.; Jha, S.; Batut, P.; Chaisson, M.; Gingeras, T. R., STAR: ultrafast universal RNA-seq aligner. *Bioinformatics* **2013,** *29* (1), 15-21.

11. Li, H.; Handsaker, B.; Wysoker, A.; Fennell, T.; Ruan, J.; Homer, N.; Marth, G.; Abecasis, G.; Durbin, R.; Genome Project Data Processing, S., The sequence alignment/map format and SAMtools. *Bioinformatics* **2009,** *25* (16), 2078-2079.

12. Robinson, J. T.; Thorvaldsdottir, H.; Winckler, W.; Guttman, M.; Lander, E. S.; Getz, G.; Mesirov, J. P., Integrative genomics viewer. *Nat. Biotechnol.* **2011,** *29* (1), 24-26.

13. Liao, Y.; Smyth, G. K.; Shi, W., featureCounts: an efficient general purpose program for assigning sequence reads to genomic features. *Bioinformatics* **2014,** *30* (7), 923-930.

14. Love, M. I.; Huber, W.; Anders, S., Moderated estimation of fold change and dispersion for RNA-seq data with DESeq2. *Genome Biol.* **2014,** *15* (12), 550.

15. Wang, E. T.; Ward, A. J.; Cherone, J. M.; Giudice, J.; Wang, T. T.; Treacy, D. J.; Lambert, N. J.; Freese, P.; Saxena, T.; Cooper, T. A.; Burge, C. B., Antagonistic regulation of mRNA expression and splicing by CELF and MBNL proteins. *Genome Res.* **2015,** *25* (6), 858-871.

16. Katz, Y.; Wang, E. T.; Airoldi, E. M.; Burge, C. B., Analysis and design of RNA sequencing experiments for identifying isoform regulation. *Nat. Methods* **2010,** *7* (12), 1009-1015.

17. Trott, O.; Olson, A. J., AutoDock Vina: improving the speed and accuracy of docking with a new scoring function, efficient optimization, and multithreading. *J. Comput. Chem.* **2010,** *31* (2), 455-461.

18. O'Boyle, N. M.; Banck, M.; James, C. A.; Morley, C.; Vandermeersch, T.; Hutchison, G. R., Open Babel: An open chemical toolbox. *J. Cheminform.* **2011,** *3*, 33.

19. Wang, J.; Wolf, R. M.; Caldwell, J. W.; Kollman, P. A.; Case, D. A., Development and testing of a general amber force field. *J. Comput. Chem.* **2004,** *25* (9), 1157-1174.

20. Gasteiger, J.; Marsili, M., Iterative partial equalization of orbital electronegativity—a rapid access to atomic charges. *Tetrahedron* **1980,** *36* (22), 3219-3228.

21. Santos-Martins, D.; Solis-Vasquez, L.; Tillack, A. F.; Sanner, M. F.; Koch, A.; Forli, S., Accelerating autoDock4 with GPUs and gradient-based local search. *J. Chem. Theory Comput.* **2021,** *17* (2), 1060-1073.

22. Wang, J.; Wang, W.; Kollman, P. A.; Case, D. A., Automatic atom type and bond type perception in molecular mechanical calculations. *J. Mol. Graph. Model.* **2006,** *25* (2), 247-260.

23. Frisch, M. J.; Trucks, G. W.; Schlegel, H. B.; Scuseria, G. E.; Robb, M. A.; Cheeseman, J. R.; Scalmani, G.; Barone, V.; Mennucci, B.; Petersson, G. A., GAUSSIAN09. *Gaussian, Inc.* **2009,** *Wallingford.*

24. Hawkins, G. D.; Cramer, C. J.; Truhlar, D. G., Pairwise solute descreening of solute charges from a dielectric medium. *Chem. Phys. Lett.* **1995,** *246* (1-2), 122-129.

25. Dupradeau, F. Y.; Pigache, A.; Zaffran, T.; Savineau, C.; Lelong, R.; Grivel, N.; Lelong, D.; Rosanski, W.; Cieplak, P., The R.E.D. tools: advances in RESP and ESP charge derivation and force field library building. *Phys. Chem. Chem. Phys.* **2010,** *12* (28), 7821-7839.

26. Case, D. A.; Walker, R. C.; Cheatham, T.; Simmerling, C.; Roitberg, A.; Merz, K. M.; Luo, R.; Darden, T.; Wang, J.; Duke, R. E.; Roe, D. R.; LeGrand, S.; Swails, J.; Cerutti, D. S.; Monard, G.; Sagui, C.; Kaus, J.; Betz, R. M.; Madej, B.; Lin, C.; Mermelstein, D.; Li, P.; Onufriev, A.; Izadi, S.; Wolf, R. M.; Wu, X.; Götz, A. W.; Gohlke, H.; Homeyer, N.; Botello-Smith, W. M.; Xiao, L.; Luchko, T.; Giese, T.; Lee, T.; Nguyen, H. T.; Nguyen, H.; Janowski, P.; Omelyan, I.; Kovalenko, A.; Kollman, P. A., AMBER 16. *University of California* **2016,** *San Francisco.*

27. Cornell, W. D.; Cieplak, P.; Bayly, C. I.; Gould, I. R.; Merz, K. M.; Ferguson, D. M.; Spellmeyer, D. C.; Fox, T.; Caldwell, J. W.; Kollman, P. A., A second generation force field for the simulation of proteins, nucleic acids, and organic molecules. *J. Am. Chem. Soc.* **2002,** *117* (19), 5179-5197.

28. Yildirim, I.; Stern, H. A.; Kennedy, S. D.; Tubbs, J. D.; Turner, D. H., Reparameterization of RNA chi torsion parameters for the AMBER force field and comparison to NMR spectra for cytidine and uridine. *J. Chem. Theory Comput.* **2010,** *6* (5), 1520-1531.

29. Wales, D. J.; Yildirim, I., Improving computational predictions of single-stranded RNA tetramers with revised alpha/gamma torsional parameters for the amber force field. *J. Phys. Chem. B* **2017,** *121* (14), 2989-2999.

30. Joung, I. S.; Cheatham, T. E., 3rd, Determination of alkali and halide monovalent ion parameters for use in explicitly solvated biomolecular simulations. *J. Phys. Chem. B* **2008,** *112* (30), 9020-9041.

31. Jorgensen, W. L.; Chandrasekhar, J.; Madura, J. D.; Impey, R. W.; Klein, M. L., Comparison of simple potential functions for simulating liquid water. *Chem. Phys.* **1983,** *79* (2), 926-935.

32. Berendsen, H. J. C.; Postma, J. P. M.; van Gunsteren, W. F.; DiNola, A.; Haak, J. R., Molecular dynamics with coupling to an external bath. *Chem. Phys.* **1984,** *81* (8), 3684-3690.

33. Ryckaert, J.; Ciccotti, G.; Berendsen, H. J. C., Numerical integration of the cartesian equations of motion of a system with constraints: molecular dynamics of n-alkanes. *J. Comp. Phys.* **1977,** *23* (3), 327-341.

34. Essmann, U.; Perera, L.; Berkowitz, M. L.; Darden, T.; Lee, H.; Pedersen, L. G., A smooth particle mesh Ewald method. *Chem. Phys.* **1995,** *103* (19), 8577-8593.

35. Liu, J.; Li, D.; Liu, X., A simple and accurate algorithm for path integral molecular dynamics with the Langevin thermostat. *J. Chem. Phys.* **2016,** *145* (2), 024103.

36. Genheden, S.; Luchko, T.; Gusarov, S.; Kovalenko, A.; Ryde, U., An MM/3D-RISM approach for ligand binding affinities. *J. Phys. Chem. B* **2010,** *114* (25), 8505-8516.

37. Kovalenko, A.; Hirata, F., Self-consistent description of a metal–water interface by the Kohn–Sham density functional theory and the three-dimensional reference interaction site model. *Chem. Phys.* **1999,** *110* (20), 10095-10112.

38. Lu, X. J.; Olson, W. K., 3DNA: a versatile, integrated software system for the analysis, rebuilding and visualization of three-dimensional nucleic-acid structures. *Nat. Protoc.* **2008,** *3* (7), 1213-1227.
